# A Colorectal Cancer Susceptibility New Variant at 4q26 in the Spanish Population Identified by Genome-Wide Association Analysis

**DOI:** 10.1371/journal.pone.0101178

**Published:** 2014-06-30

**Authors:** Luis M. Real, Agustín Ruiz, Javier Gayán, Antonio González-Pérez, María E. Sáez, Reposo Ramírez-Lorca, Francisco J. Morón, Juan Velasco, Ruth Marginet-Flinch, Eva Musulén, José M. Carrasco, Concha Moreno-Rey, Enrique Vázquez, Manuel Chaves-Conde, Jose A. Moreno-Nogueira, Manuel Hidalgo-Pascual, Eduardo Ferrero-Herrero, Sergi Castellví-Bel, Antoni Castells, Ceres Fernandez-Rozadilla, Clara Ruiz-Ponte, Angel Carracedo, Beatriz González, Sergio Alonso, Manuel Perucho

**Affiliations:** 1 Department of Structural Genomics, Neocodex, Seville, Spain; 2 Infectious Diseases and Microbiology Unit, Hospital Nuestra Señora de Valme, Seville, Spain; 3 Institute of Biomedicine of Seville (IBIS), Seville, Spain; 4 Fundació ACE, Institut Català de Neurociències Aplicades, Barcelona, Spain; 5 Bioinfosol, Seville, Spain; 6 Andalusian Center for Bioinformatic Studies (CAEBI), Seville, Spain; 7 Institute of Predictive and Personalized Medicine of Cancer (IMPPC), Badalona, Barcelona, Spain; 8 Department of Pathology, Hospital Universitario Germans Trias i Pujol (HUGTP), Badalona, Barcelona, Spain; 9 Department of Oncology, Hospital Virgen del Rocío, Seville, Spain; 10 Colorectal Surgery Unit, Hospital 12 de Octubre, Madrid, Spain; 11 Department of Gastroenterology, Hospital Clínic, University of Barcelona, CIBEREHD, Institut d’Investigacions Biomèdiques August Pi i Sunyer (IDIBAPS), Barcelona, Spain; 12 Galician Public Foundation of Genomic Medicine, Centro de Investigación Biomédica en Red de Enfermedades Raras, Genomics Medicine Group, Hospital Clínico, Santiago de Compostela, A Coruña, Spain; 13 Sanford-Burnham Medical Research Institute (SBMRI), La Jolla, California United States of America; 14 Instituciò Catalana de Recerca i Estudis Avançats (ICREA), Barcelona, Spain; National Institute of Environmental Health Sciences, United States of America

## Abstract

**Background:**

Non-hereditary colorectal cancer (CRC) is a complex disorder resulting from the combination of genetic and non-genetic factors. Genome–wide association studies (GWAS) are useful for identifying such genetic susceptibility factors. However, the single loci so far associated with CRC only represent a fraction of the genetic risk for CRC development in the general population. Therefore, many other genetic risk variants alone and in combination must still remain to be discovered. The aim of this work was to search for genetic risk factors for CRC, by performing single-locus and two-locus GWAS in the Spanish population.

**Results:**

A total of 801 controls and 500 CRC cases were included in the discovery GWAS dataset. 77 single nucleotide polymorphisms (SNP)s from single-locus and 243 SNPs from two-locus association analyses were selected for replication in 423 additional CRC cases and 1382 controls. In the meta-analysis, one SNP, rs3987 at 4q26, reached GWAS significant p-value (p = 4.02×10^−8^), and one SNP pair, rs1100508 CG and rs8111948 AA, showed a trend for two-locus association (p = 4.35×10^−11^). Additionally, our GWAS confirmed the previously reported association with CRC of five SNPs located at 3q36.2 (rs10936599), 8q24 (rs10505477), 8q24.21(rs6983267), 11q13.4 (rs3824999) and 14q22.2 (rs4444235).

**Conclusions:**

Our GWAS for CRC patients from Spain confirmed some previously reported associations for CRC and yielded a novel candidate risk SNP, located at 4q26. Epistasis analyses also yielded several novel candidate susceptibility pairs that need to be validated in independent analyses.

## Introduction

Colorectal cancer (CRC) represents globally, in terms of frequency, the third leading cause of cancer-related mortality, and the second most frequent malignant disease in Europe [1]. A minority of patients have a family history of CRC, suggesting some hereditary contribution. Germ-line mutations have been identified as the cause of inherited cancer risk in some of these CRC–prone families. Overall, high penetrance mutations are estimated to account for less than 5% of CRC cases [2]. On the other hand, the vast majority of patients with CRC have no clear evidence of having inherited the disorder and are therefore classified as “sporadic” cancer.

Sporadic CRC is considered a complex disorder resulting from the combination of genetic and non-genetic risk factors in concert with somatic genetic and epigenetic alterations. The non-Mendelian genetic risk factors are common low-risk variants distributed throughout the genome. The Genome–wide association studies (GWAS) approach is an useful tool for identifying such variants [3]. Using this approach about 30 risk genetic variants related to CRC susceptibility have been reported in the last years [4]–[15]. In spite of this, the combined effect of these variants altogether only represents a small proportion of the genetic risk for CRC development in the general population [16]. This suggests that many other risk genetic variants are yet to be discovered.

In general, GWAS have been insufficient to uncover all genes involved in complex diseases and, most importantly, they have not been very useful in isolating specific molecular pathways related to the disorders under study [17]. One of the reasons could be that single-locus approach is typically the only method applied to GWAS datasets, and this does not take into account the multigenic nature that underlies the etiology of complex diseases. Thus, new analytical methods that would help to detect more powerful genetic associations based on combination of markers have been proposed by us and others [18]–[20]. Recently, the first two-locus association study in CRC has been reported [21]. Additional studies are clearly necessary for a more comprehensive understanding of the genetic complexity of CRC susceptibility in the different human populations.

The aim of this work was to search for genetic risk factors for CRC in the Spanish population, performing a new GWAS using single-locus and two-locus genetic association analyses.

## Results

### Phase I. CRC-GWAS analysis

To identify CRC risk-associated SNPs, we designed a GWAS (NXC-GWAS) comprising 801 controls and 500 cases from the scarcely studied Spanish population (NXC-GWAS sample).

All of the SNPs were genotyped using the Affymetrix NSP I 250K chip. After quality control, 20 cases were discarded (4 discordant sex, 8 different ethnicity and 8 low sample call rate). Finally, 480 cases and 801 controls were selected for association analysis. Principal component analysis performed among this sample did not reveal population admixture (Figure S1). Age at recruitment was 58.0±9.1 years in cases and 51.9±8.8 years in controls (mean ± standard deviation). The corresponding number (percentage) of female samples were 278 (57.9%), and 368 (45.9%), respectively. Among the 262264 SNPs that can be genotyped with this chip, 83334 did not pass the quality controls (52964 SNPs were discarded due to low minor allele frequency (MAF), 2307 SNPs failed HWE, and 28333 had a significantly different rate of missingness between case and control groups). A total of 178,930 markers were finally selected for subsequent association analyses. There was no overall inflation of the test statistic (genomic inflation factor = 1.10) (see Figure S2), providing reassurance that systematic confounding factors were unlikely.

**Figure 1:**
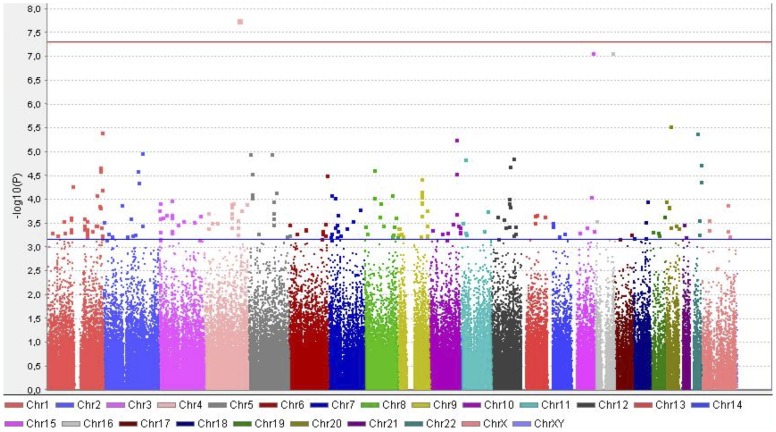
Manhattan plot of CRC-GWAS. Blue and red horizontal lines correspond to p values of 6.97×10^−4^ and 5×10^−8^ respectively.

Using Plink we carried out a single locus genetic association analysis [22]. One genetic marker, rs10446758 in chromosome 4q31.23, reached the GWAS-significant p value (p = 1.73×10^−8^), and other two markers, rs4887855 in chromosome 16q23.1 and rs7171889 in chromosome 15q26.2, showed a trend for association (p = 8.27×10^−8^ and p = 8.53×10^−8^, respectively) (figure 1) (Table S1).

We also performed a two-locus analysis using the HFCC software (see Patients and Methods section), exclusively on the SNPs that passed the quality controls. A total of 1.60×10^10^ two-locus combinations were finally obtained. After applying control direction and tracking filters, this software yielded 5x10^5^ two locus strata. Although none of them reached the cut off p value established at 3.12×10^−12^ some pairs reached values close to that threshold (Table S2).

### Phase II. Validation and meta-analysis

To test the best genetic associations observed in phase I, first, those SNPs that were included in any of the best 157 two-locus signals (Table S2) were selected. These pairs accounted for 276 single SNPs because 38 SNPs were present in more than one pair. Second, 79 SNPs from the single-locus analyses were selected according to the association p value obtained in phase I (p<6.9×10^−4^) or the probability to be successfully genotyped with the Veracode technology. Thus, a total of 355 SNPs were initially selected for the preparation of custom-made arrays. However, it was only possible to design oligonucleotide pools for 340 SNPs (79 single locus SNPs and 261 two-locus SNPs).

These genetic markers were genotyped in 423 different cases and 1448 different controls (NXC-VAL sample). Age at recruitment was 58.7±7.3 years in cases and 51.1±12.9 in controls (mean ± standard deviation). The corresponding number (percentage) of female samples was 262 (61.8%), and 920 (63.5%), respectively. Twenty SNPs did not pass the quality control (14 SNPs were not genotyped in more than 80% of samples, and 6 SNPs showed a HWE p-value <0.001 in controls). As for the samples, 66 controls were excluded (31 individuals did not achieve a genotyping call rate >80%, and 35 individuals showed some degree of relatedness to each other according to data obtained with the GRR software). Finally 423 CRC cases and 1382 controls were genotyped with 320 markers (77 single-locus and 243 two-locus selected SNPs) (Table S3). Table 1 shows those selected SNPs that were replicated in the NXC-VAL sample (p<0.05 and same effect direction). Only one SNP, rs3987 at 4q26, reached a GWAS significant p-value in the meta-analysis (Table 2). Interestingly, four more SNPs in the same genomic region showed a trend for association at GWAS-significant p-value (Table 2).

**Table 1:** SNPs validated in the phase II.

					NXC-GWAS				NXC-VAL			
CHR	Gene†	SNPs	BP‡	A1	MAF A	MAF U	P	OR	MAF A	MAF U	P	OR
4q26	*NDST3*	rs3987*	118978503	C	0.470	0.393	0.000125	1.37	0.513	0.435	8.14E-05	1.36
4q26	*NDST3*	rs1023890*	118920894	A	0.497	0.419	0.000127	1.37	0.541	0.465	0.000114	1.35
4q26	*NDST3*	rs1870481	118882468	T	0.495	0.421	2.40E-04	1.35	0.538	0.462	0.000135	1.35
4q26	*NDST3*	rs2169059	118926638	A	0.495	0.423	3.90E-04	1.33	0.541	0.466	0.000141	1.35
4q26	*NDST3*	rs1459528	118969796	G	0.492	0.417	1.96E-04	1.35	0.529	0.462	0.00062	1.30
4q32.1	*FAM198B*	rs2881373	159312083	T	0.197	0.141	1.71E-04	1.50	0.199	0.160	0.00759	1.30
5p15.1	*MYO10*	rs588367	16709570	A	0.305	0.388	2.85E-05	0.69	0.331	0.369	0.04218	0.84
5p15.1	*MYO10*	rs428263	16711495	T	0.310	0.387	7.66E-05	0.71	0.330	0.369	0.04125	0.84
5p15.1	*MYO10*	rs876095	16709803	T	0.310	0.387	8.81E-05	0.71	0.331	0.369	0.04218	0.84
12q23.1	*AK307646*	rs3858655*	95404373	T	0.167	0.126	3.46E-03	1.39	0.176	0.141	0.01329	1.29
12q23.1	*AK307646*	rs17375557*	95442660	A	0.174	0.131	3.34E-03	1.39	0.178	0.144	0.016	1.28
11q24.1	*SORL1*	rs11218350*	120957861	T	0.254	0.204	3.22E-03	1.32	0.253	0.214	0.01767	1.24
4q32.1	*TMEM144*	rs7664129*	159189132	C	0.148	0.106	1.47E-03	1.47	0.151	0.122	0.03151	1.27
13q14.11	*ENOX1*	rs4941455*	42799471	A	0.151	0.193	7.34E-03	0.74	0.156	0.186	0.04799	0.81
9p13.2	*PAX5*	rs11999298*	36884151	T	0.070	0.051	4.71E-02	1.4	0.070	0.052	0.04759	1.36

CHR: Chromosome; SNP: Single Nucleotide Polymorphism; BP: Base pair position; A1: Reference allele (minor allele). The last eight columns show the minor allele frequency in cases (MAF A), the minor allele frecuency in controls (MAF U) and, the p and the Odds Ratio (OR) values obtained in each analyzed sample.

*SNPs selected by two-locus association analyses in the NXC-GWAS sample.

† The nearest gene or the gene where the SNP is located.

‡ According to UCSC genome browser (NCBI36/hg18) and dbSNP build 130.

**Table 2:** Meta-analysis of SNPs validated in the phase II.

CHR	Gene†	SNPs	BP‡	A1	P	P(R)	OR	OR(R)	Q	I
4q26	*NDST3*	rs3987*	118978503	C	4.02E-08	4.02E-08	1.36	1.36	0.94	0
4q26	*NDST3*	rs1023890*	118920894	A	5.61E-08	5.61E-08	1.36	1.36	0.90	0
4q26	*NDST3*	rs1870481	118882468	T	1.23E-07	1.23E-07	1.35	1.35	0.99	0
4q26	*NDST3*	rs2169059	118926638	A	2.04E-07	2.04E-07	1.34	1.34	0.93	0
4q26	*NDST3*	rs1459528	118969796	G	4.57E-07	4.57E-07	1.33	1.33	0.74	0
4q32.1	*FAM198B*	rs2881373	159312083	T	6.86E-06	6.86E-06	1.39	1.39	0.35	0
5p15.1	*MYO10*	rs588367	16709570	A	0.000013	0.007262	0.76	0.76	0.10	62.7
5p15.1	*MYO10*	rs428263	16711495	T	0.000026	0.003224	0.77	0.77	0.15	51.5
5p15.1	*MYO10*	rs876095	16709803	T	0.00003	0.00312	0.77	0.77	0.15	50.5
12q23.1	*AK307646*	rs3858655*	95404373	T	0.000147	0.000147	1.34	1.34	0.63	0
12q23.1	*AK307646*	rs17375557*	95442660	A	0.000175	0.000175	1.33	1.33	0.60	0
11q24.1	*SORL1*	rs11218350*	120957861	T	0.000179	0.000179	1.28	1.28	0.61	0
4q32.1	*TMEM144*	rs7664129*	159189132	C	0.000197	0.000197	1.35	1.35	0.38	0
13q14.11	*ENOX1*	rs4941455*	42799471	A	0.001042	0.001042	0.77	0.77	0.57	0
9p13.2	*PAX5*	rs11999298*	36884151	T	0.005207	0.005207	1.38	1.38	0.92	0

CHR: Chromosome; SNP: Single Nucleotide Polymorphism; BP: Base pair position; A1: Reference allele (minor allele); P: Fixed-effects p-value; P(R): Random-effects p-value; OR: Fixed-effects Odds Ratio; OR(R): Random-effects Odds Ratio; Q: p-value for heterogeneity of OR; I: effect size for heterogeneity of OR.

*SNPs selected by two-locus association analyses in the NXC-GWAS sample.

† The nearest gene or the gene where the SNP is located.

‡ According to UCSC genome browser (NCBI36/hg18) and dbSNP build 130.

Regarding two locus analysis, only five pairs were validated in phase II (p<0.05 and same effect direction). Although none of them reached GWAS significant p-value (p<3.12×10^−12^) in the meta-analysis (Table 3), a SNP pair, rs1100508 CG and rs8111948 AA, was borderline for association (4.35×10^−11^).

**Table 3:** Best SNP×SNP interactions validated in the phase II and meta-analysis results.

			NXC- GWAS		NXC-Val		Pooled analysis	
CHR	Gene†	SNP pair	OR	p	OR	p	OR	p
6.22p/10.q22.3	*ATXN1/ANXA11*	rs9464886 AG rs2784773 AA	2.70	1.07E-07	1.52	0.009628	1.88103	9.27E-08
2q35/4q26	*MREG/NDST3*	rs3770542 AA rs1023890 GG	2.28	7.63E-07	1.59	0.000762	1.73924	1.00E-07
7q31.33/19q12	*GPR37/LOC100420587*	rs1100508 CG rs8111948 AA	3.16	1.40E-09	1.73	0.001048	2.22646	4.35E-11
12p11.22/21q22.12	*FAR2/DOPEY2*	rs4931122 AA rs7280997 TT	2.85	3.40E-09	1.59	0.007512	2.16412	1.28E-10
12q14.1/12q23.1	*FAM19A2/AK307646*	rs2731402 CC rs17375557 AG	2.20	1.34E-06	1.50	0.005473	1.73009	2.49E-07

SNP: Single Nucleotide Polymorphism; OR: HFCC Odds Ratio; P: value associated with HFCC OR (1 df).

† The nearest gene or the gene where the SNP is located.

### Result validation using additional datasets

To test whether the results could be replicated in another Spanish dataset, we used data from the Epicolon project [23]. However, none of the SNPs that were considered significant or candidates in phase II of this study replicated in this Epicolon sample.

The results obtained in our GWAS (phase I and II), and those obtained from the Epicolon cohort, were combined in an effort to see a global effect of all those SNPs checked in phase II. None of the SNPs reached the GWAS significant p-value in the combined study (Table S4). Table 4 shows the best results obtained in this study (selected from those SNPs showing an effect in the same direction in all three analyzed series. See details from those selected SNPs in Table S5).

**Table 4:** Top results in the global meta-analysis.

CHR	Gene†	SNP	BP‡	A1	p	p(R)	OR	OR(R)	Q	I
9q31.1	*LINC00587*	rs1930551*	104380162	T	0.000117	0.008854	1.42	1.43	0.1005	56.48
9q31.1	*LINC00587*	rs10990158	104335927	T	0.000118	0.010900	1.41	1.42	0.0895	58.57
7q31.1	*NRCAM*	rs2041001*	107870335	G	0.000153	0.002704	1.46	1.46	0.1989	38.08
10q25.3	*ABLIM1*	rs941853	116189165	A	0.000196	0.009045	0.79	0.78	0.125	51.92
7p15.1	*LOC402644*	rs4722778	28278588	G	0.000253	0.025260	0.82	0.82	0.0617	64.11
12q21.33	*LINC00615*	rs10506984*	89217396	G	0.000347	0.000347	0.83	0.83	0.6274	0.00
9q31.1	*LINC00587*	rs7039568	104361604	T	0.000417	0.030730	1.37	1.40	0.0532	65.92
9q31.1	*LINC00587*	rs16921774*	104336206	T	0.000479	0.025540	1.36	1.38	0.0743	61.54
9q31.1	*LINC00587*	rs10990136	104298657	T	0.000831	0.039330	1.35	1.37	0.0581	64.85
5q21.1	*ST8SIA4*	rs2120913*	100096374	A	0.000852	0.000852	0.85	0.85	0.8767	0.00
9q31.1	*LINC00587*	rs7024470	104361506	G	0.000941	0.030740	1.34	1.36	0.0777	60.87

CHR: Chromosome; BP: Base pair position; SNP: Single Nucleotide Polymorphism; A1: Reference allele (minor allele); p: Fixed-effects p-value; p(R): Random-effects p-value; OR: Fixed-effects Odds Ratio; OR(R): Random-effects Odds Ratio; Q: p-value for heterogeneity of OR; I: effect size for heterogeneity of OR.

*SNPs selected by two-locus association analyses in the NXC-GWAS sample.

† The nearest gene or the gene where the SNP is located.

‡ According to UCSC genome browser (NCBI36/hg18) and dbSNP build 130.

Regarding two-locus HFCC analysis, no SNP-pair showed a significant and consistent effect (in the same direction) when the 3 samples (NXC-GWAS, NXC-Val and Epicolon) were analyzed together.

### Analysis of SNPs previously associated with CRC

Only one of the previously associated SNPs with CRC risk was successfully genotyped in our GWAS. In order to cover a greater number of these SNPs we imputed genotypes using CEU HapMap data base and Plink software. After imputation, we obtained a total of 1,371,009 SNPs for subsequent analysis. A total of 16 previously reported as CRC associated SNPs were available at the time of the analysis (Table 5). Of these, five SNPs located at 3q36.2 (rs10936599), 8q24 (rs10505477), 8q24.21(rs6983267), 11q13.4 (rs3824999) and 14q22.2 (rs4444235), showed nominal association with CRC in our GWAS, and with effects in the same direction than those previously reported (Table 5). Two more SNPs located at 8q23.3 (rs16892766) and 12q13.13 (rs7136702) showed a trend to nominal association with CRC in our study, again with the effect in the same direction than previously reported (Table 5).

**Table 5:** Results of previously reported SNPs that were successfully genotyped or imputed in our analysis.

						NXC-GWAS			
CHR	Gene†	SNP	Reported allele	Reported OR	Reference	F A	F U	OR	p
1q25.3	*LAMC1*	rs10911251	A	1.09	[10]	0.570	0.553	1.07	0.4898
1q41	*DUSP10*	rs6691170	G	0.94	[6]	0.626	0.647	0.91	0.2945
1q41	*DUSP10*	rs6687758	A	0.92	[6]	0.797	0.817	0.88	0.2157
2q32.3	*OBFC2A*	rs11903757	C	1.16	[10]	0.142	0.139	1.02	0.8047
**3q26.2**	***MYNN***	**rs10936599**	**C**	**1.08**	[6]	0.821	0.786	**1.25**	**0.0386**
8q23.3	*EIF3H*	rs16892766	A	0.92	[14]	0.905	0.926	0.75	0.0575
**8q24**	***DQ515899***	**rs10505477** *	**A**	**1.17**	[15]	0.571	0.508	**1.28**	**0.0019**
**8q24.21**	***DQ515899***	**rs6983267**	**G**	**1.21**	[12]	0.571	0.508	**1.28**	**0.0019**
9p24	*UHRF2*	rs719725	A	1.07	[15]	0.598	0.605	0.97	0.7259
10p14	*BC031880*	rs10795668	A	0.89	[14]	0.304	0.299	1.02	0.7826
**11q13.4**	***POLD3***	**rs3824999**	**G**	**1.08**	[5]	0.534	0.487	**1.20**	**0.0242**
11q23	*LOC120376*	rs3802842	A	0.90	[11]	0.702	0.709	0.96	0.6972
12q13.13	*LARP4*	rs7136702	C	0.94	[6]	0.612	0.647	0.85	0.0713
**14q22.2**	***BMP4***	**rs4444235**	**C**	**1.09**	[7]	0.539	0.493	**1.20**	**0.0435**
15q13.3	*SCG5*	rs16969681	C	0.84	[13]	0.876	0.900	1.27	0.0618
16q22.1	*CDH1*	rs9929218	A	0.91	[7]	0.262	0.274	0.93	0.5028

CHR: Chromosome; SNP: Single Nucleotide Polymorphism. The last four columns show the allele reported frequency in cases (F A), in controls (F U) and, the p and the Odds Ratio (OR) values obtained in the NXC-GWAS sample.

In bold type, SNPs with a nominal p-value below 0.05.

† The nearest gene or the gene where the SNP is located.

*SNPs genotyped (not imputed).

We could not test the candidate SNPs reported by Fernandez-Rozadilla *et al*. [23] in their CRC-GWAS performed in the Spanish population (Epicolon sample), because those candidates were not covered or successfully genotyped/imputed in our study.

We also tested two-locus interactions between rs1571218 (20p12.3) and rs10879357 (12q21.1) previously associated with CRC [21]. Applying general lineal models we did not observe any evidence of interaction between them in our dataset (data not shown).

## Discussion

We present a new two-phase CRC-GWAS carried out in the Spanish population for single locus and also for two-locus association using our HFCC software [18]. One marker, rs3987 at 4q26, reached association with CRC susceptibility at GWAS significant p-value. Furthermore, one SNP pair, rs1100508 CG rs8111948 AA (located at 7q31.33 and 19q12, respectively), showed also a trend for epistatic association.

In spite of limitations of our GWAS - low density of genomic coverage of the DNA-chip, and a moderate sample size - we replicated 5 of the 16 SNPs previously associated with CRC. In addition, the majority of these 16 SNPs in our GWAS study were in the same direction than in the published reports (Table 5). Furthermore, regression analysis showed good concordance of the odds ratios (Figure S3). These data together suggest that our study is in line with previously published CRC GWAS analyses.

In our two-phase CRC-GWAS, one marker, namely rs3987 at 4q26, exhibited association with CRC susceptibility at GWAS significant p-value. This SNP is located in an intergenic region of 4q26 between *TRAM1L1* and *NDST3 genes* (∼500 kb and ∼180 kb, respectively). Several studies have already suggested the presence of cancer genes in 4q region [24], [25], and it has also been reported that somatic deletions at 4q26 are frequent in CRC [26], [27]. Interestingly, the *NDST4* gene, located also at 4q26, and belonging to the same family than *NDST3*, has been identified as a possible tumour suppressor gene in CRC [27].

The two-locus analysis revealed that one of the SNPs pairs, rs1100508 CG and rs8111948 AA (located at 7q31.33 and 19q12, respectively), showed a trend for association. These SNPs are in intergenic regions located at 7q31.33 and 19q12. The closest gene to rs1100508 is *GPR37*, a member of the G protein-coupled receptor family that is known to interact with Parkin, albeit its function remains to be fully characterized. On the other hand, rs8111948 is located between *LINC00662* and *LINC00906* (∼500 kb and ∼600 kb, respectively), two loci belonging to the long non-coding RNA (lncRNA) family. If the association of this SNP pair is confirmed, the nature of that interaction will need to be further characterized.

We also studied the markers associated with CRC from our two-phase GWAS in an independent Spanish GWAS dataset (Epicolon), but none of these associations replicated. However, since our GWAS could validate more of the well-stablished CRC associations than the Epicolon GWAS [23], we consider that the candidates derived from our study deserve to be validated in further meta-analysis including other GWAS and validation studies performed in the Spanish population, or in a more general Caucasian population.

According to the GWAS catalogue from NIH (http://www.genome.gov/26525384), and previous works in this topic [5]–[15], neither the variants associated with CRC reported in table 1 or 2, nor variants included in the SNP pairs reported in table 3 (or in linkage disequilibrium with them) have been previously associated with CRC. Since the majority of these previous studies were not particularly performed in the Southern Caucasian population, our results could be specific for that population. An alternative explanation would be that they are false positive. The clustering of several SNPs at the same 4q26, and the replication of previously reported associations argues against this possibility.

Although our results could not be replicated in the independent Epicolon sample, we carried out a meta-analysis taking into account the three samples analyzed here (NXC-GWAS, NXC-VAL, and Epicolon). None of the SNPs, or combinations of them, were replicated in the three samples, but the best signals comprise several SNPs in linkage disequilibrium at 9q31.1, within or close to *LINC00587* locus (Table 4). This gene also belongs to the lncRNA family involved in cellular differentiation and proliferation as post-transcriptional regulators of splicing or as molecular decoys for miRNA [28], [29]. The expression of lncRNAs is deregulated in many different cancers, including colon cancer [30], and some studies suggest a role in cancer initiation, progression and metastasis [31]. The association reported in previous GWAS between CRC susceptibility and SNPs located at 8q24 could be due to the *PRNCR1* locus, a lncRNA member [32].

Interestingly, a high proportion of SNPs found to be associated with CRC in our study discovery phase (tables 1, 2 and 4), were selected by the two-locus analysis. This suggests that in addition to identify epistatic interactions, our two-locus analysis method (HFCC software) can also improve the capture of single signals in the genome related to CRC susceptibility in particular and thus in multigenic disease in general. This is an enticing hypothesis that might be confirmed if some of these SNPs are validated in future studies. On the other hand, the results of our two-locus analyses suggest that the interaction signals have no more powerful predictive value than single loci for CRC susceptibility because of the failure to detect SNP pairs associated with CRC at GWAS significant p-value. This observation, together with the absence of statistically significant results in our global meta-analysis, as well as the lack of replication of the only SNP pair interaction previously reported as associated with CRC [21] suggests that the role of genetic factors in CRC susceptibility might be more intricate that previously thought.

In conclusion, we have carried out a CRC-GWAS in the Spanish population that is in line with some previously reported associations and yielded a new candidate SNP for CRC susceptibility at 4q26 that needs to be validated in future studies. Our two-locus study also provides evidence of the high level of complexity in genetic cancer risk.

## Materials and Methods

### Patients

Subjects in phase I were 801 controls from the Spanish general population (which were previously described [33]) and 500 cases diagnosed of CRC with pathological confirmation (NXC-GWAS sample). In phase II 1448 controls and 423 cases of CRC were used (NXC-VAL sample). CRC samples were collected in two different Spanish hospitals (Hospital Universitario Virgen del Rocío in Seville and Hospital Universitario 12 de Octubre in Madrid) from November 2002 to April 2008. The control samples included in phase II were collected during the same time period in several primary health care centres from all around Spain. These samples have been previously used as controls in other association studies performed for different diseases in the Spanish population [34]. Therefore, a total of 923 CRC cases and 2249 controls from the Spanish general population were included in this study. All individuals enrolled were Caucasian with registered Spanish ancestors (two generations) as recorded by clinical researchers.

### Ethics Statement

The ethics committees from Hospital Universitario Virgen del Rocío, Sevilla, and Hospital Universitario 12 de Octubre, Madrid, as well as Neocodex approved the research protocol, which was in compliance with national legislation and performed according to the ethical guidelines of the Declaration of Helsinki [35]. Written informed consent was obtained from all individuals included in this work.

### External genotyping dataset

Genotyping data of selected SNPs from other GWAS performed in the Spanish population (Epicolon cohort) [23] were used as a reference for the results obtained herein. Specifically, this cohort consisted in 882 cases and 473 controls ascertained through the Epicolon II project and 194 additional controls from the Spanish National DNA bank.

### Genotyping

Peripheral blood from all cases and controls were used to isolate germline DNA from leukocytes. DNA extraction was performed automatically according to standard procedures using the Magnapure DNA isolation system (Roche Diagnostics, Mannheim, Germany).

For genome-wide genotyping we used the Afymetrix NspI chip as previously described [33]. For genotyping of selected SNPs in the NXC-VAL sample we employed custom Golden Gate protocols and Veracode genotyping assay (Illumina, San Diego, California USA) according to the manufacturer’s instructions.

### Data availability

Association results for genotyped and imputed SNPs are provided as compressed Plink files (Dataset S1 and Dataset S2). Case by case genotype data is available on request to the ethics committee of the IMPPC (Instituto de Medicina Predictiva y Personalizada del Cáncer) according to the conditions established in the Spanish Law for Biomedical Research (Ley 14/2007, de 3 de julio).

### Quality control analyses

For samples genotyped using the Affymetrix platform, we performed an extensive quality control using Affymetrix Genotyping Console Software (http://www.affymetrix.com) and Plink [22]. Only individuals with a sample call rate above 93% were later re-called with the Bayesian Robust Linear Model with Malalanobis (BRLMM) distance algorithm, ran with default parameters. BRLLM improved call rates in most samples. Self-reported sex was compared to sex assigned by chromosome X genotypes, and discrepancies were resolved or samples removed. The program Graphical Representation of Relationships (GRR) [36] was used to check sample relatedness and to correct potential sample mislabelling, duplications, or contaminations. SNPs were selected to have a call rate above 95% (in each case, control, and combined group), and a minor allele frequency above 1% (again in each case, control, and combined group). SNPs that deviated grossly from Hardy-Weinberg equilibrium (HWE) (P-value <10^−4^) in control samples were also removed. We also removed SNPs with a significantly different rate of missingness (P-value <5×10^−4^) between case and control samples.

Similarly, SNPs genotyped in the phase II were subjected to quality control filters. Thus, those SNPs that were not successfully genotyped in at least 80% of individuals, and those with a p-value for Hardy–Weinberg equilibrium (HWE) lower than 0.001 were discarded. In addition, individuals with more than 10% of missing genotype data or that showed relatedness to each other were also excluded.

### Principal components analysis

Principal component analysis was carried out with EIGENSOFT [37], [38] to evaluate population admixture within our population, and to identify individuals as outliers. We ran the SMARTPCA program with default parameters, excluding chromosome X markers and using independent SNPs (pairwise r^2^<0.1). To minimize the effect of linkage disequilibrium in the analysis, long-range linkage disequilibrium regions previously reported [39] or detected in our population were also excluded. Individuals identified as outliers (six standard deviations or more along one of the top ten principal components) were removed from all subsequent analyses. Principal component analysis was run together with other HapMap European and worldwide populations to detect individuals of different ethnicities.

### Single locus association analysis

Unadjusted single-locus allelic (1 degree of freedom, df) association analyses were carried out using Plink software [22], independently within each group of subjects from phase I or phase II. Meta-analysis tool in Plink was used to analyze combined data from different datasets. In these studies, fixed effects models were employed when no evidence of heterogeneity was found. Otherwise random effects models were employed. A GWAS significant p-value was established at 5×10^−8^ [40]. Plink was also employed to estimate the genomic inflation factor. Haploview software [41] was employed for graphical representation of the GWAS single locus analysis results (Manhattan plot). The concordance of the detected effect and the reported effect for those SNPs previously found to be associated with CRC was analyzed by linear regression after logarithmic transformation of the odds ratios.

### Two-locus association analysis

Aiming to detect potential epistatic *loci*, we explored the entire universe of two-locus interactions (all SNP x SNP interactions) using the Hypothesis Free Clinical Cloning (HFCC) software as described previously [18]. Briefly, in phase I three different replication groups of 160 cases and 267 controls were created. In order to be considered a preliminary positive result, the chi-square (1 df) test cut-off value was set at 6.64 (p<0.01) and the direction of the effect had to be the same for each replication group (which approximates to p<1×10^−6^ over all three replication groups).

To explore the nature and strength of interactions in selected two-locus patterns, we further evaluated epistasis among selected markers using Alambique software [18]. Specifically, Alambique was programmed to measure departure from additive models by calculating the Synergy index, AP or RERI statistics, whilst departure from multiplicity was measured by computing strata-specific odds ratios and case-only interaction test. The algorithms included in the Alambique software have been previously described elsewhere [42], [43].

During the validation process, those SNPs selected by HFCC that were successfully genotyped in the NXC-VAL sample were analyzed for replication. In this case two groups of replication were created: the NXC-GWAS sample and the NXC-VAL sample. When the selected pairs were also studied in the Epicolon cohort, three groups of replication were created: NXC-GWAS, NXC-VAL and the Epicolon sample.

Multiple-testing correction was applied in those studies taking into account the number of different SNP-pairs generated. Thus, the p-value threshold was established at (p = 3.12×10^−12^ (0.05/total number of SNP-pairs generated in the phase I dataset).

To test the two-locus interaction that was previously associated with CRC susceptibility [21], i.e. rs1571218 (20p12.3) and rs10879357 (12q21.1), we modelled the interaction using linear regression with SPSS software 19.0 (IBM Corporation, Somers, NY, USA).

### Imputation

We imputed genotypes using HapMap phase 2 CEU founders (n = 60) as a reference panel with Plink [22]. Genotype calls with high quality scores (info>0.8) were used in subsequent association analyses.

## Supporting Information

Figure S1 **Scatterplot of the two main eigenvectors obtained from the principal component analysis performed on 801 controls (green circles) and 480 cases (blue circles) selected for the phase-I association study.**
(PDF)

Figure S2 **Quantile-Quantile (Q-Q) plot of the observed and expected χ2 values obtained from the study of the association between SNP genotype and colorectal cancer risk.**
(PDF)

Figure S3 **Correlation between the effects (OR) found in the NXC-GWAS and the reported effects for the 16 SNPs previously found to associate with CRC risk.** The blue line represents perfect correlation. The green line indicates the correlation excluding the outlayer rs16969681 (red circle). This SNP was originally reported in the UK2 GWAS with an OR of 1.247, that reached GWAS significant after meta analysis with other Northern Europe GWAS but was not replicated in the Epicolon GWAS of Southern Europe. The coefficient of determination (R2) and p-value (Pearson’s P) of the correlation are indicated. Without excluding the rs16969681, the coefficient of determination and p-value were 0.28 and 0.035, respectively.
(PDF)

Table S1 **Best phase I results obtained by Plink.**
(DOC)

Table S2 **Best SNP×SNP interactions obtained by HFCC software.**
(DOC)

Table S3 **SNPs included in the phase II and meta-analysis results.**
(DOC)

Table S4 **SNPs included in the stage II and global meta-analysis results.**
(DOC)

Table S5 **Details of the results obtained in each sample from those SNPs that showed the best results in the global meta-analysis.**
(DOC)

Dataset S1 **Plink association file of genotyped SNPs.**
(ZIP)

Dataset S2 **Plink association file of imputed SNPs.**
(ZIP)
