# Supplementary figures and images for: A Colorectal Cancer Susceptibility New Variant at 4q26 in the Spanish Population Identified by Genome-Wide Association Analysis
Source: PLoS One. 2014 Jun 30;9(6):e101178. doi: 10.1371/journal.pone.0101178 (PMC4076321; doi:10.1371/journal.pone.0101178)

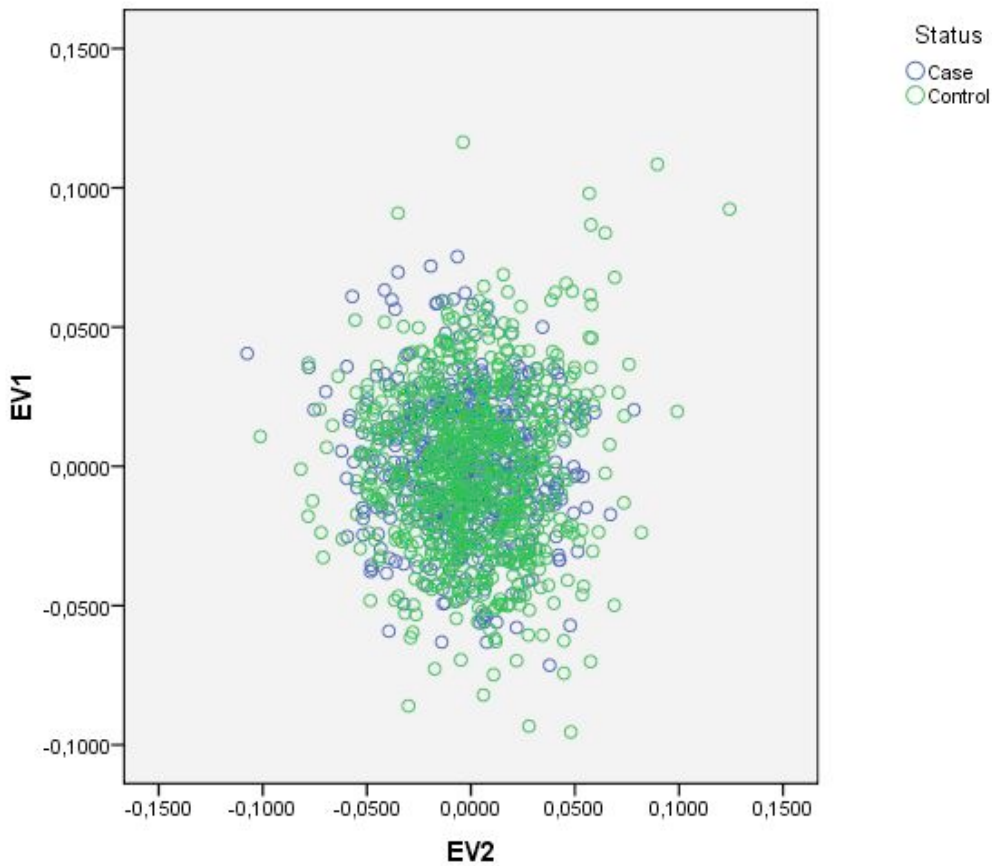

Supplement: Figure S1 — Scatterplot of the two main eigenvectors obtained from the principal component analysis performed on 801 controls (green circles) and 480 cases (blue circles) selected for the phase-I association study. (PDF) [file pone.0101178.s001.pdf]

Chi-square Q-Q Plot of A2

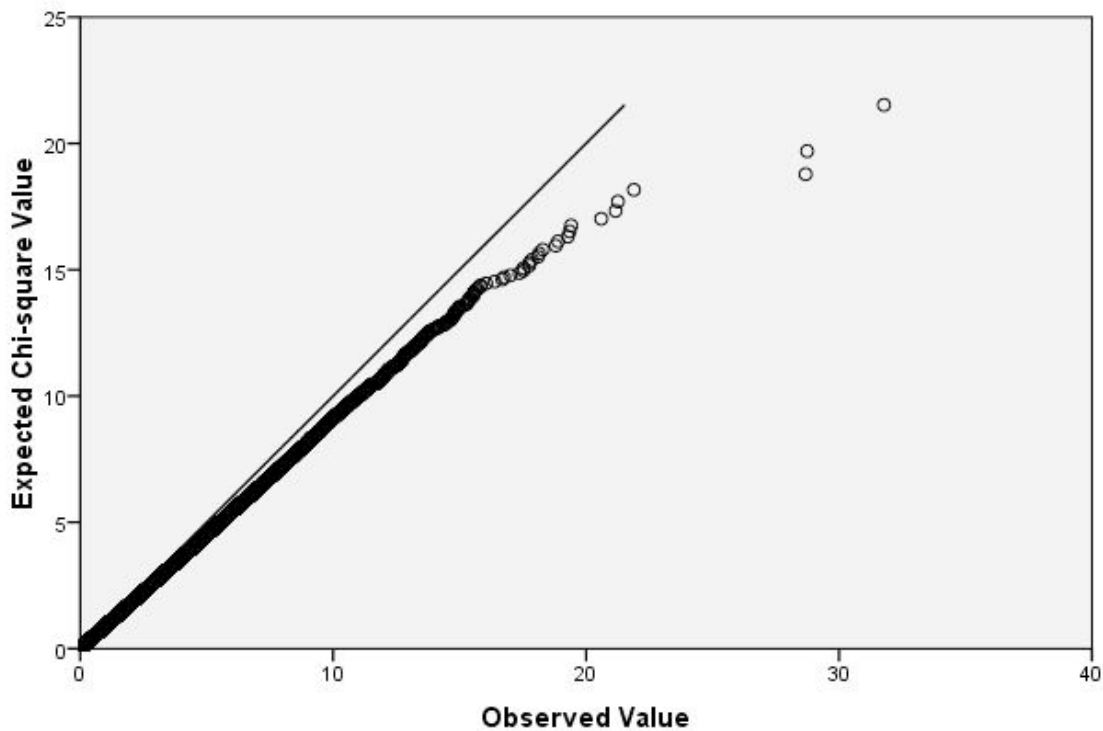

Supplement: Figure S2 — Quantile-Quantile (Q-Q) plot of the observed and expected χ2 values obtained from the study of the association between SNP genotype and colorectal cancer risk. (PDF) [file pone.0101178.s002.pdf]

# NXC-GWAS OR vs Reported OR

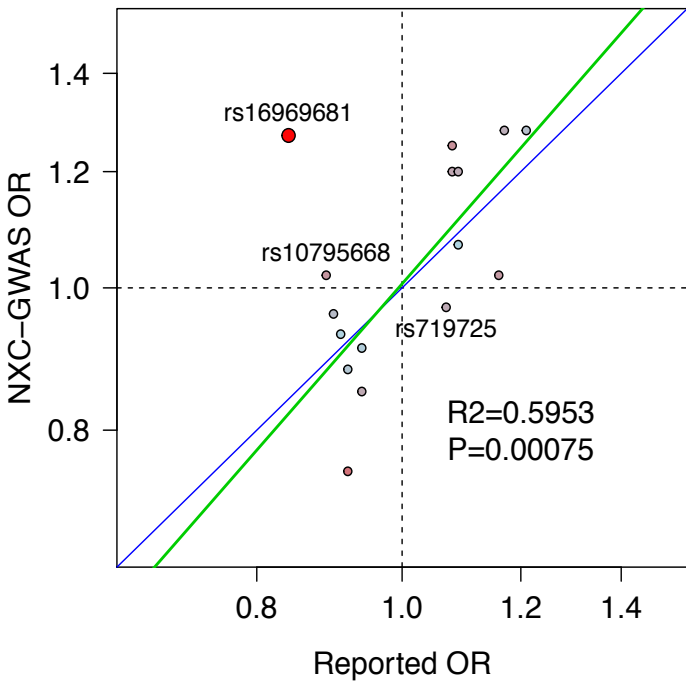

Supplement: Figure S3 — Correlation between the effects (OR) found in the NXC-GWAS and the reported effects for the 16 SNPs previously found to associate with CRC risk. The blue line represents perfect correlation. The green line indicates the correlation excluding the outlayer rs16969681 (red circle). This SNP was originally reported in the UK2 GWAS with an OR of 1.247, that reached GWAS significant after meta analysis with other Northern Europe GWAS but was not replicated in the Epicolon GWAS of Southern Europe. The coefficient of determination (R2) and p-value (Pearson’s P) of the correlation are indicated. Without excluding the rs16969681, the coefficient of determination and p-value were 0.28 and 0.035, respectively. (PDF) [file pone.0101178.s003.pdf]
