# Supplementary material for: A Colorectal Cancer Susceptibility New Variant at 4q26 in the Spanish Population Identified by Genome-Wide Association Analysis
Source: PLoS One. 2014 Jun 30;9(6):e101178. doi: 10.1371/journal.pone.0101178 (PMC4076321; doi:10.1371/journal.pone.0101178)
Supplement: Table S1 — Best phase I results obtained by Plink. (DOC) [file pone.0101178.s004.doc]

**Table S1**. Best phase I results obtained by Plink

| **CHR** | **SNP** | **BP*** | **A1** | **MAF cases** | **MAF controls** | **A2** | **P** | **OR** |
| --- | --- | --- | --- | --- | --- | --- | --- | --- |
| 4 | rs10446758 | 149667035 | A | 0.4022 | 0.5194 | G | 1,73E-08 | 0.6225 |
| 16 | rs4887855 | 74989899 | T | 0.03486 | 0.09207 | C | 8,27E-08 | 0.3562 |
| 15 | rs7171889 | 92377186 | C | 0.1236 | 0.06178 | A | 8,53E-08 | 2.143 |
| 20 | rs16986484 | 24276308 | C | 0.1481 | 0.08732 | T | 2,89E-06 | 1.817 |
| 1 | rs3014578 | 239698430 | C | 0.09935 | 0.05096 | T | 4,00E-06 | 2.055 |
| 22 | rs242896 | 32765815 | G | 0.5199 | 0.4261 | C | 4,20E-06 | 1.459 |
| 10 | rs17091953 | 116253478 | G | 0.2328 | 0.1604 | A | 5,66E-06 | 1.588 |
| 2 | rs559113 | 169411175 | A | 0.4024 | 0.4936 | T | 1,06E-05 | 0.6908 |
| 5 | rs16879116 | 7847503 | G | 0.3177 | 0.4045 | A | 1,09E-05 | 0.6855 |
| 5 | rs7734355 | 102775829 | C | 0.07569 | 0.03598 | T | 1,13E-05 | 2.194 |
| 12 | rs1477102 | 95526343 | G | 0.3983 | 0.4869 | T | 1,38E-05 | 0.6978 |
| 11 | rs2403583 | 20334676 | C | 0.241 | 0.1699 | A | 1,45E-05 | 1.551 |
| 22 | rs6009047 | 45683534 | T | 0.2947 | 0.3781 | C | 1,90E-05 | 0.6873 |
| 12 | rs1922397 | 80507780 | T | 0.08096 | 0.1379 | C | 2,05E-05 | 0.5506 |
| 1 | rs6664738 | 232002372 | T | 0.3458 | 0.2665 | C | 2,09E-05 | 1.455 |
| 8 | rs7838116 | 40048312 | A | 0.06799 | 0.12 | G | 2,38E-05 | 0.5351 |
| 2 | rs7578749 | 148038283 | A | 0.2073 | 0.2833 | G | 2,50E-05 | 0.6614 |
| 1 | rs4649259 | 232007684 | G | 0.3542 | 0.275 | A | 2,52E-05 | 1.446 |
| 10 | rs17091955 | 116253976 | T | 0.2254 | 0.1589 | C | 2,83E-05 | 1.54 |
| 5 | rs588367 | 16709570 | A | 0.3056 | 0.3883 | G | 2,85E-05 | 0.6933 |
| 6 | rs4490643 | 165800135 | T | 0.2017 | 0.2753 | C | 3,08E-05 | 0.6649 |
| 9 | rs10990113 | 104268870 | C | 0.08581 | 0.0454 | T | 3,73E-05 | 1.974 |
| 22 | rs5769234 | 45683168 | C | 0.2979 | 0.3775 | G | 4,25E-05 | 0.6997 |
| 2 | rs707025 | 154896769 | A | 0.4467 | 0.365 | C | 4,41E-05 | 1.404 |
| 1 | rs1359414 | 111963158 | C | 0.2854 | 0.2147 | T | 5,17E-05 | 1.461 |
| 1 | rs6429174 | 238239018 | G | 0.3927 | 0.315 | T | 6,16E-05 | 1.406 |
| 9 | rs7039568 | 104361604 | T | 0.08021 | 0.04261 | C | 6,90E-05 | 1.959 |
| 5 | rs12519633 | 121967053 | T | 0.1177 | 0.07134 | C | 7,00E-05 | 1.737 |
| 9 | rs10990158 | 104335927 | T | 0.08577 | 0.04688 | A | 7,40E-05 | 1.908 |
| 5 | rs428263 | 16711495 | T | 0.31 | 0.3875 | C | 7,66E-05 | 0.7102 |
| 1 | rs17666678 | 215844513 | T | 0.2432 | 0.1782 | C | 7,94E-05 | 1.482 |
| 8 | rs4870723 | 121297860 | A | 0.4332 | 0.5138 | C | 7,95E-05 | 0.7234 |
| 5 | rs388887 | 16711524 | T | 0.3075 | 0.3847 | C | 8,09E-05 | 0.7103 |
| 7 | rs6943487 | 12711855 | C | 0.2317 | 0.3038 | G | 8,14E-05 | 0.6914 |
| 9 | rs1930551 | 104380162 | T | 0.08021 | 0.04307 | G | 8,67E-05 | 1.937 |
| 15 | rs4842907 | 84239094 | A | 0.09539 | 0.1492 | C | 8,73E-05 | 0.6014 |
| 5 | rs876095 | 16709803 | T | 0.3104 | 0.3872 | C | 8,81E-05 | 0.7124 |
| 8 | rs7821033 | 40047207 | G | 0.08368 | 0.1348 | A | 9,13E-05 | 0.586 |
| 7 | rs4722778 | 28278588 | G | 0.2357 | 0.308 | C | 9,24E-05 | 0.693 |
| 5 | rs685875 | 16709506 | A | 0.3111 | 0.3876 | C | 9,29E-05 | 0.7133 |
| 12 | rs2710901 | 77433629 | G | 0.2322 | 0.1694 | A | 9,77E-05 | 1.483 |
| 3 | rs4373099 | 54254022 | T | 0.4078 | 0.3317 | C | 1,07E-04 | 1.387 |
| 5 | rs13354207 | 110179690 | C | 0.2604 | 0.195 | G | 1,08E-04 | 1.454 |
| 20 | rs1810636 | 2602925 | T | 0.3355 | 0.4131 | G | 1,11E-04 | 0.7173 |
| 18 | rs4477825 | 63210306 | C | 0.5283 | 0.4493 | G | 1,11E-04 | 1.373 |
| 9 | rs16921774 | 104336206 | T | 0.08559 | 0.04762 | C | 1,15E-04 | 1.872 |
| 9 | rs10990136 | 104298657 | T | 0.08125 | 0.04443 | C | 1,18E-04 | 1.902 |
| 3 | rs794184 | 4426994 | C | 0.3211 | 0.397 | T | 1,19E-04 | 0.7185 |
| 12 | rs1521386 | 77418817 | G | 0.2328 | 0.1706 | A | 1,19E-04 | 1.475 |
| 8 | rs13273088 | 70656528 | G | 0.24 | 0.1767 | A | 1,19E-04 | 1.471 |
| 4 | rs10518461 | 126383748 | G | 0.1695 | 0.2338 | C | 1,22E-04 | 0.6689 |
| 4 | rs7657701 | 182529026 | C | 0.06318 | 0.1094 | T | 1,25E-04 | 0.5491 |
| 4 | rs3987 | 118978503 | C | 0.4706 | 0.3931 | T | 1,26E-04 | 1.373 |
| 4 | rs1023890 | 118920894 | A | 0.4979 | 0.4192 | G | 1,27E-04 | 1.374 |
| 23 | rs6608501 | 115308935 | C | 0.3652 | 0.2796 | T | 1,28E-04 | 1.483 |
| 2 | rs6730095 | 77151964 | A | 0.3531 | 0.2806 | C | 1,32E-04 | 1.399 |
| 1 | rs9660543 | 227349555 | T | 0.1349 | 0.1938 | C | 1,38E-04 | 0.6488 |
| 20 | rs10485515 | 14797853 | T | 0.1276 | 0.1852 | C | 1,40E-04 | 0.6434 |
| 12 | rs4842316 | 78498696 | G | 0.4441 | 0.3673 | A | 1,43E-04 | 1.376 |
| 4 | rs12648654 | 118915046 | A | 0.5 | 0.4226 | G | 1,44E-04 | 1.366 |
| 1 | rs16859609 | 232000522 | G | 0.3435 | 0.2722 | A | 1,49E-04 | 1.399 |
| 20 | rs1998087 | 14764382 | A | 0.1324 | 0.1908 | C | 1,50E-04 | 0.6473 |
| 7 | rs17160621 | 138268222 | T | 0.1333 | 0.08636 | C | 1,64E-04 | 1.628 |
| 3 | rs4594610 | 989666 | T | 0.04947 | 0.09016 | C | 1,65E-04 | 0.5252 |
| 9 | rs6478690 | 126797131 | C | 0.4618 | 0.3857 | T | 1,71E-04 | 1.367 |
| 4 | rs2881373 | 159312083 | T | 0.1977 | 0.1411 | C | 1,71E-04 | 1.5 |
| 11 | rs538645 | 118216279 | G | 0.2531 | 0.1904 | A | 1,78E-04 | 1.441 |
| 4 | rs12503362 | 14452104 | T | 0.1027 | 0.06195 | C | 1,89E-04 | 1.733 |
| 5 | rs2416248 | 110206705 | C | 0.2771 | 0.2122 | T | 1,89E-04 | 1.423 |
| 4 | rs1459528 | 118969796 | G | 0.4927 | 0.417 | A | 1,96E-04 | 1.358 |
| 10 | rs941853 | 116189165 | A | 0.1357 | 0.1935 | G | 2,04E-04 | 0.6542 |
| 7 | rs1029621 | 40832234 | A | 0.2803 | 0.2152 | G | 2,07E-04 | 1.42 |
| 13 | rs9540846 | 66110941 | A | 0.06737 | 0.112 | T | 2,07E-04 | 0.5726 |
| 3 | rs7645545 | 54392362 | T | 0.3559 | 0.2857 | C | 2,08E-04 | 1.382 |
| 3 | rs421653 | 32486654 | T | 0.452 | 0.3777 | G | 2,10E-04 | 1.359 |
| 13 | rs354789 | 57024535 | T | 0.2153 | 0.1573 | G | 2,16E-04 | 1.471 |
| 3 | rs3774230 | 181004332 | C | 0.2724 | 0.3427 | T | 2,17E-04 | 0.7182 |
| 9 | rs7024470 | 104361506 | G | 0.08209 | 0.04581 | A | 2,21E-04 | 1.863 |
| 8 | rs12545053 | 65236159 | C | 0.3305 | 0.4036 | T | 2,25E-04 | 0.7295 |
| 12 | rs840895 | 27001290 | G | 0.04228 | 0.07985 | A | 2,28E-04 | 0.5088 |
| 19 | rs17239559 | 59398234 | C | 0.369 | 0.4432 | T | 2,31E-04 | 0.7346 |
| 13 | rs1283145 | 100234116 | C | 0.05833 | 0.1001 | T | 2,33E-04 | 0.5567 |
| 3 | rs4234541 | 17157701 | C | 0.5156 | 0.4407 | T | 2,35E-04 | 1.351 |
| 1 | rs550437 | 104121003 | G | 0.2944 | 0.3663 | C | 2,38E-04 | 0.722 |
| 8 | rs1457461 | 135167438 | C | 0.2115 | 0.1544 | T | 2,39E-04 | 1.469 |
| 4 | rs1870481 | 118882468 | T | 0.4958 | 0.4213 | C | 2,40E-04 | 1.351 |
| 3 | rs1473348 | 54384035 | C | 0.3558 | 0.2857 | T | 2,41E-04 | 1.381 |
| 1 | rs4656349 | 160316448 | G | 0.4078 | 0.3356 | A | 2,44E-04 | 1.363 |
| 2 | rs2008776 | 118997264 | G | 0.5189 | 0.4439 | A | 2,46E-04 | 1.351 |
| 5 | rs6888588 | 110225865 | G | 0.2771 | 0.2135 | A | 2,50E-04 | 1.412 |
| 3 | rs2117637 | 5813180 | A | 0.275 | 0.2116 | T | 2,51E-04 | 1.413 |
| 12 | rs931586 | 53360703 | G | 0.2264 | 0.2928 | C | 2,58E-04 | 0.707 |
| 1 | rs2646249 | 104110655 | C | 0.2944 | 0.365 | A | 2,58E-04 | 0.7257 |
| 23 | rs1923093 | 32838128 | C | 0.5374 | 0.4494 | G | 2,66E-04 | 1.423 |
| 22 | rs2255957 | 40571318 | A | 0.1099 | 0.1623 | G | 2,66E-04 | 0.6376 |
| 4 | rs1912555 | 144641172 | T | 0.522 | 0.4474 | C | 2,70E-04 | 1.349 |
| 4 | rs12512157 | 144662185 | A | 0.5229 | 0.4487 | G | 2,75E-04 | 1.346 |
| 7 | rs2041001 | 107870335 | G | 0.07218 | 0.03933 | A | 2,80E-04 | 1.9 |
| 16 | rs17440477 | 6452734 | T | 0.2716 | 0.3416 | A | 2,83E-04 | 0.7187 |
| 1 | rs7545661 | 73749076 | T | 0.04486 | 0.08312 | C | 2,84E-04 | 0.518 |
| 1 | rs10913182 | 174718843 | C | 0.05263 | 0.09238 | T | 2,85E-04 | 0.5458 |
| 3 | rs1165898 | 105305259 | A | 0.2662 | 0.2041 | G | 2,88E-04 | 1.414 |
| 2 | rs2059411 | 1721959 | C | 0.3211 | 0.2544 | T | 2,89E-04 | 1.386 |
| 3 | rs16863323 | 152542758 | T | 0.2318 | 0.2984 | C | 2,92E-04 | 0.7097 |
| 18 | rs2851855 | 55394340 | A | 0.1333 | 0.08801 | C | 2,92E-04 | 1.594 |
| 11 | rs2343231 | 5727416 | T | 0.3884 | 0.3181 | C | 3,04E-04 | 1.361 |
| 4 | rs12233687 | 31058796 | A | 0.07484 | 0.04096 | G | 3,07E-04 | 1.894 |
| 13 | rs1374482 | 57054311 | T | 0.2474 | 0.1873 | C | 3,09E-04 | 1.426 |
| 14 | rs4982731 | 22655173 | C | 0.2985 | 0.234 | T | 3,10E-04 | 1.393 |
| 4 | rs17086320 | 56683501 | A | 0.1367 | 0.1923 | G | 3,11E-04 | 0.6655 |
| 6 | rs4709443 | 157960957 | G | 0.3277 | 0.3994 | C | 3,18E-04 | 0.7329 |
| 10 | rs1998756 | 100219522 | A | 0.3758 | 0.3066 | G | 3,28E-04 | 1.361 |
| 3 | rs17021431 | 84427280 | T | 0.1572 | 0.1083 | G | 3,30E-04 | 1.536 |
| 7 | rs17169864 | 34577115 | G | 0.09708 | 0.05879 | A | 3,30E-04 | 1.721 |
| 6 | rs3778533 | 1777935 | C | 0.1429 | 0.09625 | T | 3,34E-04 | 1.565 |
| 5 | rs1350294 | 110205180 | A | 0.274 | 0.2119 | C | 3,36E-04 | 1.404 |
| 21 | rs11909376 | 21377027 | A | 0.1216 | 0.07862 | G | 3,39E-04 | 1.622 |
| 9 | rs10986505 | 126780259 | A | 0.4515 | 0.3793 | G | 3,44E-04 | 1.347 |
| 2 | rs548367 | 169377708 | A | 0.2766 | 0.3448 | G | 3,44E-04 | 0.7265 |
| 1 | rs3790733 | 215866331 | G | 0.2711 | 0.2091 | T | 3,45E-04 | 1.407 |
| 20 | rs396221 | 45707496 | A | 0.5355 | 0.4624 | C | 3,45E-04 | 1.341 |
| 1 | rs1415257 | 160328668 | G | 0.4247 | 0.3538 | A | 3,51E-04 | 1.349 |
| 9 | rs10986471 | 126675534 | A | 0.4591 | 0.3871 | G | 3,53E-04 | 1.344 |
| 10 | rs4752352 | 121681508 | C | 0.1875 | 0.2484 | T | 3,54E-04 | 0.6981 |
| 8 | rs2013421 | 79975099 | A | 0.2443 | 0.3102 | T | 3,57E-04 | 0.7189 |
| 1 | rs1187781 | 211613720 | A | 0.2591 | 0.1978 | T | 3,57E-04 | 1.418 |
| 8 | rs17332483 | 3945370 | A | 0.1615 | 0.1124 | T | 3,60E-04 | 1.521 |
| 14 | rs8015478 | 22655858 | A | 0.2975 | 0.2338 | C | 3,60E-04 | 1.388 |
| 12 | rs2710922 | 77423482 | G | 0.1273 | 0.08365 | A | 3,61E-04 | 1.599 |
| 8 | rs17330462 | 126680229 | C | 0.3305 | 0.2644 | T | 3,62E-04 | 1.374 |
| 12 | rs7134701 | 102726821 | T | 0.2741 | 0.2122 | C | 3,62E-04 | 1.401 |
| 15 | rs7183242 | 64883712 | G | 0.2179 | 0.2821 | A | 3,74E-04 | 0.7092 |
| 4 | rs2270677 | 144686894 | C | 0.5251 | 0.4524 | T | 3,75E-04 | 1.338 |
| 20 | rs6083315 | 23811795 | G | 0.2951 | 0.3645 | A | 3,75E-04 | 0.7298 |
| 12 | rs17124269 | 59546866 | C | 0.1373 | 0.1922 | G | 3,77E-04 | 0.6689 |
| 7 | rs17717216 | 28448949 | C | 0.2427 | 0.3081 | T | 3,78E-04 | 0.7197 |
| 1 | rs1270972 | 235180964 | T | 0.25 | 0.3159 | C | 3,83E-04 | 0.722 |
| 4 | rs2169059 | 118926638 | A | 0.4958 | 0.4237 | C | 3,90E-04 | 1.338 |
| 10 | rs11016976 | 131540391 | C | 0.2385 | 0.1804 | T | 3,90E-04 | 1.423 |
| 4 | rs12510758 | 14451896 | A | 0.1002 | 0.0618 | G | 3,92E-04 | 1.691 |
| 9 | rs10975323 | 5857435 | C | 0.1253 | 0.0815 | T | 3,94E-04 | 1.614 |
| 20 | rs6128768 | 58162219 | G | 0.5095 | 0.4369 | C | 3,95E-04 | 1.338 |
| 9 | rs4740497 | 1151515 | T | 0.1458 | 0.09938 | C | 3,95E-04 | 1.547 |
| 7 | rs7793055 | 79841434 | C | 0.1148 | 0.166 | T | 3,96E-04 | 0.6515 |
| 6 | rs4706313 | 68785785 | G | 0.04017 | 0.07519 | T | 4,08E-04 | 0.5148 |
| 1 | rs1337061 | 160329876 | A | 0.3952 | 0.3262 | G | 4,18E-04 | 1.349 |
| 1 | rs10913183 | 174719823 | A | 0.05335 | 0.09187 | G | 4,23E-04 | 0.557 |
| 1 | rs1930184 | 104169421 | C | 0.2969 | 0.365 | T | 4,25E-04 | 0.7346 |
| 10 | rs17469997 | 8495938 | C | 0.09062 | 0.1373 | G | 4,26E-04 | 0.626 |
| 6 | rs9360320 | 68931796 | A | 0.2718 | 0.3391 | C | 4,32E-04 | 0.7275 |
| 23 | rs5928104 | 32827171 | G | 0.5199 | 0.4353 | A | 4,44E-04 | 1.405 |
| 11 | rs11224806 | 100883042 | T | 0.06789 | 0.1104 | A | 4,48E-04 | 0.5868 |
| 1 | rs16844287 | 197385336 | A | 0.1126 | 0.1633 | G | 4,50E-04 | 0.6504 |
| 3 | rs11715179 | 54259337 | T | 0.428 | 0.3583 | A | 4,53E-04 | 1.34 |
| 6 | rs6935203 | 144475717 | A | 0.1464 | 0.1003 | G | 4,54E-04 | 1.54 |
| 15 | rs4984349 | 92894673 | A | 0.1439 | 0.1989 | G | 4,56E-04 | 0.6772 |
| 23 | rs17326660 | 114601927 | A | 0.2377 | 0.3149 | G | 4,63E-04 | 0.6786 |
| 7 | rs728585 | 40873222 | A | 0.2699 | 0.2089 | G | 4,63E-04 | 1.4 |
| 10 | rs10734092 | 131550598 | A | 0.324 | 0.2597 | G | 4,77E-04 | 1.366 |
| 19 | rs12150997 | 7063265 | T | 0.1526 | 0.2085 | C | 4,78E-04 | 0.6836 |
| 3 | rs310724 | 4326751 | T | 0.2595 | 0.2 | C | 4,79E-04 | 1.402 |
| 1 | rs504877 | 104117020 | T | 0.2965 | 0.3639 | A | 4,82E-04 | 0.7365 |
| 1 | rs10873788 | 76140445 | C | 0.3184 | 0.2544 | A | 4,86E-04 | 1.369 |
| 1 | rs6425387 | 174732623 | T | 0.05952 | 0.1 | G | 4,87E-04 | 0.5696 |
| 1 | rs16826818 | 22433872 | C | 0.1811 | 0.2405 | A | 4,87E-04 | 0.6988 |
| 10 | rs12415708 | 77586614 | C | 0.1127 | 0.07242 | T | 4,90E-04 | 1.627 |
| 11 | rs1353649 | 20210175 | A | 0.333 | 0.2676 | G | 4,91E-04 | 1.366 |
| 15 | rs4924344 | 32989045 | A | 0.3169 | 0.2531 | T | 4,94E-04 | 1.369 |
| 10 | rs10734091 | 131550488 | T | 0.3288 | 0.2644 | C | 4,99E-04 | 1.363 |
| 19 | rs8111948 | 33517335 | G | 0.4781 | 0.4076 | A | 5,00E-04 | 1.331 |
| 3 | rs1869156 | 56318391 | C | 0.3628 | 0.2954 | T | 5,01E-04 | 1.358 |
| 5 | rs9292848 | 42342138 | G | 0.3238 | 0.3926 | C | 5,07E-04 | 0.741 |
| 9 | rs6474838 | 14454254 | G | 0.2927 | 0.231 | C | 5,11E-04 | 1.378 |
| 8 | rs2975696 | 10150116 | A | 0.2547 | 0.3194 | C | 5,18E-04 | 0.7283 |
| 7 | rs2723412 | 13445949 | C | 0.2375 | 0.1806 | A | 5,20E-04 | 1.413 |
| 6 | rs6935462 | 144554694 | A | 0.07741 | 0.04454 | T | 5,22E-04 | 1.8 |
| 10 | rs10998031 | 52973403 | A | 0.3948 | 0.465 | G | 5,23E-04 | 0.7504 |
| 14 | rs1476611 | 70765790 | A | 0.08854 | 0.05326 | C | 5,26E-04 | 1.727 |
| 2 | rs7589798 | 20342651 | A | 0.1086 | 0.06929 | G | 5,27E-04 | 1.636 |
| 12 | rs12816747 | 104872513 | C | 0.4301 | 0.5013 | T | 5,27E-04 | 0.7508 |
| 6 | rs2975046 | 30023128 | C | 0.2073 | 0.2683 | T | 5,28E-04 | 0.7133 |
| 4 | rs7685063 | 144662800 | A | 0.523 | 0.4521 | G | 5,29E-04 | 1.329 |
| 9 | rs1572745 | 2074739 | T | 0.1823 | 0.1317 | C | 5,32E-04 | 1.47 |
| 2 | rs4954231 | 135732984 | A | 0.191 | 0.1386 | G | 5,33E-04 | 1.467 |
| 11 | rs12274598 | 24196876 | G | 0.09002 | 0.05381 | T | 5,39E-04 | 1.74 |
| 17 | rs4793456 | 68069433 | C | 0.285 | 0.224 | T | 5,43E-04 | 1.38 |
| 22 | rs1009544 | 40569294 | G | 0.08977 | 0.1355 | C | 5,47E-04 | 0.629 |
| 8 | rs1021898 | 122308217 | T | 0.4021 | 0.4725 | C | 5,51E-04 | 0.7509 |
| 6 | rs4709439 | 157954505 | G | 0.3585 | 0.2925 | C | 5,53E-04 | 1.352 |
| 12 | rs7962891 | 95751533 | T | 0.3135 | 0.2506 | G | 5,55E-04 | 1.366 |
| 1 | rs2050516 | 239773156 | T | 0.3937 | 0.3265 | C | 5,56E-04 | 1.34 |
| 9 | rs2289631 | 126773747 | C | 0.4588 | 0.3884 | T | 5,60E-04 | 1.335 |
| 5 | rs13190345 | 179609585 | A | 0.136 | 0.09211 | G | 5,63E-04 | 1.551 |
| 1 | rs6689487 | 174733116 | C | 0.05365 | 0.09171 | T | 5,65E-04 | 0.5615 |
| 2 | rs4848884 | 124130522 | A | 0.1052 | 0.06679 | G | 5,69E-04 | 1.643 |
| 7 | rs2074777 | 50767383 | C | 0.2803 | 0.2199 | T | 5,69E-04 | 1.382 |
| 1 | rs946527 | 46258557 | T | 0.3351 | 0.2709 | C | 5,71E-04 | 1.356 |
| 19 | rs35793600 | 37151136 | A | 0.2227 | 0.1671 | G | 5,72E-04 | 1.428 |
| 1 | rs10926514 | 239772973 | G | 0.3933 | 0.3259 | T | 5,75E-04 | 1.341 |
| 2 | rs12613761 | 104122546 | G | 0.325 | 0.2615 | C | 5,76E-04 | 1.359 |
| 8 | rs6988293 | 121303479 | A | 0.5272 | 0.4569 | G | 5,76E-04 | 1.326 |
| 5 | rs17644917 | 169027137 | C | 0.03887 | 0.07234 | A | 5,81E-04 | 0.5186 |
| 9 | rs2891140 | 20140132 | A | 0.05638 | 0.09499 | G | 5,88E-04 | 0.5693 |
| 2 | rs6736078 | 35300712 | T | 0.1237 | 0.08198 | C | 5,91E-04 | 1.581 |
| 14 | rs7145941 | 49056851 | G | 0.4454 | 0.5158 | T | 5,94E-04 | 0.7539 |
| 23 | rs4474149 | 122458057 | G | 0.4006 | 0.4832 | A | 5,97E-04 | 0.7148 |
| 9 | rs4743091 | 97417012 | A | 0.1778 | 0.2353 | G | 6,00E-04 | 0.7028 |
| 8 | rs7017932 | 143197436 | G | 0.1025 | 0.1501 | A | 6,06E-04 | 0.6469 |
| 21 | rs2830727 | 27441703 | A | 0.3449 | 0.4136 | G | 6,09E-04 | 0.7466 |
| 1 | rs10913185 | 174725602 | C | 0.05428 | 0.09176 | G | 6,09E-04 | 0.5681 |
| 9 | rs463774 | 126992646 | C | 0.3344 | 0.2706 | G | 6,13E-04 | 1.354 |
| 7 | rs1034876 | 34574506 | C | 0.09599 | 0.05945 | T | 6,14E-04 | 1.68 |
| 7 | rs6948280 | 10083530 | T | 0.1385 | 0.1913 | G | 6,14E-04 | 0.6801 |
| 18 | rs2298536 | 3065872 | G | 0.1823 | 0.1323 | C | 6,31E-04 | 1.462 |
| 18 | rs2564486 | 55397273 | T | 0.1308 | 0.08812 | G | 6,38E-04 | 1.556 |
| 1 | rs2050504 | 239774362 | T | 0.3954 | 0.3287 | C | 6,50E-04 | 1.335 |
| 12 | rs11610353 | 27282464 | G | 0.04906 | 0.08489 | T | 6,60E-04 | 0.5561 |
| 6 | rs1188862 | 139275856 | G | 0.4152 | 0.3474 | A | 6,64E-04 | 1.334 |
| 17 | rs9914758 | 20851016 | G | 0.3563 | 0.2917 | A | 6,80E-04 | 1.344 |
| 3 | rs12634249 | 4461303 | T | 0.2306 | 0.2922 | G | 6,83E-04 | 0.7259 |
| 3 | rs9854434 | 176172614 | C | 0.4507 | 0.3825 | G | 6,88E-04 | 1.325 |
| 2 | rs779379 | 12998058 | A | 0.1534 | 0.1074 | G | 6,90E-04 | 1.506 |
| 7 | rs5014691 | 14817686 | A | 0.2898 | 0.2287 | G | 6,97E-04 | 1.376 |
| 10 | rs10823772 | 72934436 | T | 0.2065 | 0.2662 | C | 7,01E-04 | 0.7174 |
| 3 | rs4074087 | 4656908 | T | 0.07987 | 0.04657 | G | 7,03E-04 | 1.777 |

CHR: Chromosome, SNP: Single Nucleotide Polymorphism, BP: Base pair position, A1: Reference allele, A2: Alternative allele, MAF: minor allele frequency, OR: Odds Ratio

*According to UCSC genome browser (NCBI36/hg18) and dbSNP build 130.
