# Supplementary material for: A Colorectal Cancer Susceptibility New Variant at 4q26 in the Spanish Population Identified by Genome-Wide Association Analysis
Source: PLoS One. 2014 Jun 30;9(6):e101178. doi: 10.1371/journal.pone.0101178 (PMC4076321; doi:10.1371/journal.pone.0101178)
Supplement: Table S2 — Best SNP×SNP interactions obtained by HFCC software. (DOC) [file pone.0101178.s005.doc]

**Table S2**. Best SNP×SNP interactions obtained by HFCC software.

| **SNP pair** | **CHR(M1)** | **BP*** | **CHR(M2)** | **BP*** | **HFCC diplotype** | **HFCC OR (diplotype)** | | **p (HFCC)** | **OR(M1)** | **OR(M2)** | **Mult.** |
| --- | --- | --- | --- | --- | --- | --- | --- | --- | --- | --- | --- |
| rs685875xrs8012146 | 5 | 16709506 | 14 | 62215852 | (55159 3 143935 3) | | 2.42 | 5.2012E-11 | 1.01 | 1.12 | 1.13 |
| rs9289008xrs7027349 | 3 | 116302696 | 9 | 82242161 | (35891 2 102198 2) | | 2.51 | 6.9606E-11 | 0.91 | 0.84 | 0.77 |
| rs10064352xrs7898110 | 5 | 75512971 | 10 | 53562357 | (58724 2 109852 2) | | 2.43 | 1.6437E-10 | 0.93 | 1.04 | 0.97 |
| rs4663128xrs1941065 | 2 | 234881472 | 18 | 10012266 | (28006 2 160165 3) | | 0.42 | 1.7568E-10 | 1.15 | 1.11 | 1.29 |
| rs9660543xrs914480 | 1 | 227349555 | 13 | 73830626 | (11989 3 137697 2) | | 2.14 | 6.9665E-10 | 1.29 | 1.04 | 1.35 |
| rs4419372xrs10038955 | 3 | 61580831 | 5 | 155681482 | (32625 2 63931 3) | | 0.28 | 7.4465E-10 | 1.38 | 1.27 | 1.76 |
| rs4659986xrs4687889 | 1 | 238866166 | 3 | 119020129 | (12925 2 36126 3) | | 2.97 | 8.3783E-10 | 1.03 | 1.06 | 1.09 |
| rs8012146xrs724879 | 14 | 62215852 | 18 | 71944229 | (143935 3 164509 2) | | 2.17 | 1.0499E-09 | 1.01 | 0.85 | 0.86 |
| rs7518650xrs9289008 | 1 | 215538497 | 3 | 116302696 | (11267 3 35891 2) | | 2.15 | 1.1108E-09 | 1.18 | 0.86 | 1.01 |
| rs17644183xrs7774757 | 2 | 168343798 | 6 | 155417681 | (23845 2 76527 2) | | 2.27 | 1.1514E-09 | 0.93 | 1.03 | 0.97 |
| rs1428558xrs10764825 | 5 | 73887534 | 10 | 130559927 | (58627 2 115373 2) | | 2.26 | 1.2499E-09 | 0.94 | 0.86 | 0.81 |
| rs1100508xrs8111948 | 7 | 124132594 | 19 | 33517335 | (85358 2 165462 1) | | 3.16 | 1.4064E-09 | 0.89 | 0.87 | 0.78 |
| rs6664738xrs1323341 | 1 | 232002372 | 9 | 14443010 | (12335 3 99295 3) | | 0.45 | 1.488E-09 | 0.92 | 1.01 | 0.93 |
| rs10794730xrs2824723 | 10 | 1237022 | 21 | 18595079 | (105978 2 170978 3) | | 2.3 | 1.5111E-09 | 0.67 | 0.81 | 0.55 |
| rs1252101xrs1374482 | 1 | 235170243 | 13 | 57054311 | (12559 3 136344 2) | | 2.73 | 1.5189E-09 | 1.07 | 1.17 | 1.26 |
| rs2139404xrs421653 | 2 | 100608026 | 3 | 32486654 | (19898 2 31078 1) | | 3.77 | 1.607E-09 | 0.97 | 0.97 | 0.95 |
| rs1874406xrs9959583 | 1 | 64547498 | 18 | 20993778 | (3158 2 160672 2) | | 2.53 | 1.7899E-09 | 1.04 | 1.22 | 1.27 |
| rs10514631xrs221752 | 2 | 19767453 | 6 | 165779431 | (15040 2 77395 3) | | 2.68 | 2.3494E-09 | 0.69 | 1.02 | 0.71 |
| rs279108xrs9639329 | 5 | 100110254 | 7 | 19366356 | (60128 2 79263 3) | | 2.32 | 2.5768E-09 | 0.97 | 0.81 | 0.79 |
| rs2120913xrs10485515 | 5 | 100096374 | 20 | 14797853 | (60126 2 167804 3) | | 2.07 | 2.6438E-09 | 0.76 | 1.01 | 0.77 |
| rs17661170xrs8038473 | 4 | 171411895 | 15 | 96097480 | (52245 2 150939 2) | | 2.56 | 2.6574E-09 | 0.81 | 0.98 | 0.8 |
| rs9660543xrs4941455 | 1 | 227349555 | 13 | 42799471 | (11989 3 135457 3) | | 2.01 | 2.9448E-09 | 0.84 | 0.68 | 0.58 |
| rs4650704xrs4426363 | 1 | 173385546 | 16 | 78595960 | (8499 3 155181 3) | | 0.47 | 2.9906E-09 | 1.21 | 1.22 | 1.48 |
| rs11123800xrs12106650 | 2 | 99831454 | 22 | 15551377 | (19840 2 173322 2) | | 2.38 | 3.0841E-09 | 0.79 | 0.94 | 0.74 |
| rs17717216xrs11116324 | 7 | 28448949 | 12 | 83254244 | (79965 3 130353 3) | | 2.22 | 3.1E-09 | 0.9 | 0.82 | 0.74 |
| rs12645979xrs1372622 | 4 | 11477195 | 18 | 2619368 | (41767 2 159568 2) | | 2.51 | 3.1E-09 | 0.97 | 0.83 | 0.81 |
| rs10497616xrs10998835 | 2 | 183986209 | 10 | 70922049 | (24915 3 111312 2) | | 2.09 | 3.2971E-09 | 0.95 | 0.83 | 0.8 |
| rs10918985xrs4687889 | 1 | 167022584 | 3 | 119020129 | (8066 2 36126 3) | | 2.73 | 3.3655E-09 | 0.89 | 1 | 0.89 |
| rs935466xrs7975794 | 9 | 87308057 | 12 | 127973026 | (102625 2 133280 2) | | 2.27 | 3.3655E-09 | 0.89 | 0.87 | 0.78 |
| rs6474838xrs214760 | 9 | 14454254 | 20 | 2223976 | (99298 2 166773 2) | | 2.91 | 3.4002E-09 | 1.15 | 0.97 | 1.12 |
| rs4931122xrs7280997 | 12 | 28992287 | 21 | 36580379 | (126838 3 172553 3) | | 2.85 | 3.4177E-09 | 1.01 | 1.15 | 1.17 |
| rs2215595xrs11116324 | 7 | 28424667 | 12 | 83254244 | (79962 3 130353 3) | | 2.27 | 3.453E-09 | 0.9 | 0.9 | 0.81 |
| rs4653300xrs6988293 | 1 | 37796641 | 8 | 121303479 | (1733 2 95811 1) | | 2.97 | 3.8463E-09 | 1.03 | 1.03 | 1.07 |
| rs2743937xrs7898110 | 6 | 29915902 | 10 | 53562357 | (68087 3 109852 2) | | 2.23 | 4.0699E-09 | 0.8 | 0.97 | 0.78 |
| rs13314127xrs1075905 | 3 | 189952658 | 16 | 9015599 | (40459 3 151902 2) | | 2.74 | 4.0699E-09 | 0.85 | 0.75 | 0.64 |
| rs3892789xrs9568005 | 6 | 111403162 | 13 | 47594325 | (73467 2 135792 2) | | 3.28 | 4.1331E-09 | 0.94 | 0.99 | 0.94 |
| rs2114862xrs1374482 | 12 | 105120036 | 13 | 57054311 | (131952 3 136344 2) | | 2.24 | 4.3734E-09 | 0.72 | 0.78 | 0.56 |
| rs10953515xrs6988293 | 7 | 106080918 | 8 | 121303479 | (84370 2 95811 1) | | 3.28 | 4.4186E-09 | 0.89 | 1.08 | 0.97 |
| rs2975696xrs7087228 | 8 | 10150116 | 10 | 8304160 | (88616 3 106752 3) | | 2.15 | 1.1617E-08 | 1.22 | 1.01 | 1.24 |
| rs7140637xrs1201987 | 14 | 49892268 | 20 | 58736287 | (143077 2 170442 2) | | 2.34 | 2.2685E-08 | 1.04 | 1.01 | 1.05 |
| rs10932541xrs9959583 | 2 | 215099110 | 18 | 20993778 | (26663 3 160672 2) | | 2.04 | 2.5017E-08 | 1.03 | 1.09 | 1.13 |
| rs193841xrs7096673 | 7 | 105374089 | 10 | 88522232 | (84307 2 112495 2) | | 4.9 | 2.8604E-08 | 0.91 | 1.16 | 1.05 |
| rs6664738xrs3858655 | 1 | 232002372 | 12 | 95404373 | (12335 3 131188 3) | | 0.5 | 3.6445E-08 | 0.76 | 0.77 | 0.59 |
| rs5018999xrs1735090 | 4 | 114251101 | 7 | 152626909 | (48475 2 87050 2) | | 2.95 | 6.0106E-08 | 0.85 | 0.94 | 0.8 |
| rs4148206xrs2735096 | 2 | 43923285 | 6 | 30023730 | (16622 3 68105 1) | | 3 | 6.3289E-08 | 0.8 | 0.98 | 0.78 |
| rs1885952xrs2055846 | 13 | 18548695 | 16 | 61733578 | (133560 2 153973 3) | | 2.36 | 6.7681E-08 | 0.95 | 0.9 | 0.86 |
| rs10157868xrs11016976 | 1 | 96049209 | 10 | 131540391 | (5371 3 115468 3) | | 0.5 | 8.1084E-08 | 0.92 | 0.86 | 0.8 |
| rs17644183xrs6893150 | 2 | 168343798 | 5 | 80545692 | (23845 2 59060 2) | | 2.41 | 9.3216E-08 | 1.1 | 0.94 | 1.03 |
| rs3774108xrs12509776 | 3 | 10898193 | 4 | 116608823 | (29544 2 48654 3) | | 2.07 | 9.8157E-08 | 0.6 | 0.71 | 0.43 |
| rs10488779xrs9950038 | 11 | 79494759 | 18 | 24262559 | (120418 3 160889 1) | | 2.68 | 1.0661E-07 | 0.96 | 0.86 | 0.83 |
| rs9464886xrs2784773 | 6 | 16495782 | 10 | 81909780 | (67043 2 111908 3) | | 2.7 | 1.0772E-07 | 0.83 | 0.8 | 0.66 |
| rs17746541xrs17651115 | 3 | 60354293 | 5 | 75475886 | (32479 2 58715 2) | | 2.03 | 1.0772E-07 | 1.11 | 1.07 | 1.18 |
| rs10804515xrs12768718 | 3 | 116184747 | 10 | 49376207 | (35890 2 109529 3) | | 1.92 | 1.1111E-07 | 0.74 | 1.06 | 0.79 |
| rs2588223xrs10817664 | 8 | 17431383 | 9 | 116472734 | (89319 3 104826 3) | | 0.35 | 1.1285E-07 | 1.15 | 1.13 | 1.31 |
| rs1348213xrs5023585 | 3 | 77398339 | 5 | 109381500 | (33826 2 60758 3) | | 0.51 | 1.1761E-07 | 0.91 | 0.83 | 0.76 |
| rs9862718xrs5023585 | 3 | 54399741 | 5 | 109381500 | (31993 2 60758 2) | | 2.88 | 1.2514E-07 | 1.24 | 1.19 | 1.48 |
| rs9322458xrs10498420 | 6 | 154635053 | 14 | 49483793 | (76486 2 143048 1) | | 4.19 | 1.3246E-07 | 1.03 | 0.79 | 0.82 |
| rs17021431xrs11909376 | 3 | 84427280 | 21 | 21377027 | (34248 3 171223 3) | | 0.53 | 1.3876E-07 | 1.06 | 0.98 | 1.04 |
| rs1961460xrs1528641 | 4 | 82393522 | 11 | 14043034 | (46417 2 116732 3) | | 0.32 | 1.5388E-07 | 0.9 | 1.4 | 1.27 |
| rs6664738xrs4442966 | 1 | 232002372 | 2 | 11003195 | (12335 2 14374 3) | | 2.08 | 1.6802E-07 | 1.13 | 1.04 | 1.19 |
| rs17293620xrs2474568 | 4 | 184539543 | 10 | 38423153 | (53264 3 109256 2) | | 1.86 | 1.7331E-07 | 0.98 | 1.02 | 1 |
| rs4951701xrs9542253 | 1 | 209232629 | 13 | 69752150 | (10803 3 137327 1) | | 3.28 | 1.8441E-07 | 0.87 | 0.78 | 0.68 |
| rs851681xrs1374482 | 7 | 147107591 | 13 | 57054311 | (86794 2 136344 2) | | 2.29 | 2.0241E-07 | 1.11 | 1.32 | 1.47 |
| rs11016976xrs1521386 | 10 | 131540391 | 12 | 77418817 | (115468 3 129955 3) | | 0.53 | 2.0557E-07 | 0.85 | 0.85 | 0.73 |
| rs4663128xrs10818226 | 2 | 234881472 | 9 | 120523994 | (28006 2 105179 3) | | 0.48 | 2.1315E-07 | 0.83 | 0.94 | 0.79 |
| rs1165898xrs11745313 | 3 | 105305259 | 5 | 147552456 | (35202 3 63314 2) | | 0.49 | 2.2447E-07 | 0.88 | 1.01 | 0.9 |
| rs421653xrs1528502 | 3 | 32486654 | 7 | 84932177 | (31078 1 83250 3) | | 3.05 | 2.3396E-07 | 1.09 | 0.85 | 0.93 |
| rs10759949xrs11020505 | 9 | 119632012 | 11 | 93117781 | (105110 3 121539 2) | | 2.55 | 2.568E-07 | 0.84 | 1.06 | 0.9 |
| rs1165898xrs1930551 | 3 | 105305259 | 9 | 104380162 | (35202 3 103761 3) | | 0.54 | 2.568E-07 | 1.26 | 0.73 | 0.93 |
| rs7762948xrs11197206 | 6 | 34094069 | 10 | 117172335 | (68452 3 114326 1) | | 0.36 | 2.7045E-07 | 1.3 | 3.16 | 4.11 |
| rs2979378xrs4408437 | 3 | 126779879 | 13 | 57133947 | (36593 2 136349 2) | | 2.23 | 2.7898E-07 | 0.68 | 0.79 | 0.54 |
| rs4340489xrs12354899 | 2 | 14583293 | 10 | 86977616 | (14655 2 112393 2) | | 2.09 | 3.0308E-07 | 0.88 | 0.86 | 0.76 |
| rs4594610xrs4377227 | 3 | 989666 | 18 | 20987826 | (28510 3 160670 2) | | 1.82 | 3.2251E-07 | 1.42 | 0.9 | 1.28 |
| rs11795150xrs6515674 | 9 | 16095961 | 20 | 25597055 | (99453 1 168726 3) | | 0.32 | 3.4319E-07 | 3.34 | 2.19 | 7.33 |
| rs3987xrs2178683 | 4 | 118978503 | 12 | 80465589 | (48822 1 130140 3) | | 2.29 | 3.5771E-07 | 0.55 | 0.75 | 0.41 |
| rs7641152xrs17133918 | 3 | 194502461 | 7 | 50679766 | (40833 3 81603 2) | | 2.13 | 3.5957E-07 | 1.06 | 1.13 | 1.21 |
| rs1825238xrs12546220 | 4 | 60176065 | 8 | 70536282 | (44941 3 92461 3) | | 0.51 | 3.652E-07 | 1.27 | 1.27 | 1.62 |
| rs1989658xrs4939756 | 15 | 23376708 | 18 | 43977260 | (146726 2 162256 2) | | 2.32 | 3.7092E-07 | 1.16 | 1.06 | 1.24 |
| rs11888564xrs6769328 | 2 | 173274379 | 3 | 79455526 | (24163 3 33956 2) | | 1.86 | 3.8863E-07 | 1.23 | 1.16 | 1.42 |
| rs4709680xrs1930551 | 6 | 163520791 | 9 | 104380162 | (77184 2 103761 2) | | 5.16 | 3.8863E-07 | 0.83 | 1.09 | 0.92 |
| rs1499571xrs1922397 | 6 | 119871783 | 12 | 80507780 | (74034 2 130143 3) | | 1.85 | 3.9268E-07 | 0.98 | 1.4 | 1.37 |
| rs2008776xrs10173668 | 2 | 118997264 | 2 | 192344408 | (20927 1 25363 2) | | 2.96 | 3.9472E-07 | 1.18 | 1.02 | 1.21 |
| rs17233898xrs1189041 | 3 | 5807331 | 14 | 55931553 | (29060 2 143517 3) | | 0.45 | 4.093E-07 | 1.95 | 1.26 | 2.46 |
| rs752036xrs1870301 | 8 | 101695417 | 15 | 93412015 | (94382 2 150737 3) | | 0.52 | 4.1571E-07 | 0.84 | 0.8 | 0.67 |
| rs1023102xrs12933414 | 13 | 72723017 | 16 | 9401468 | (137583 2 151930 3) | | 2.25 | 4.1787E-07 | 0.88 | 0.8 | 0.71 |
| rs17334527xrs17499330 | 2 | 77117777 | 2 | 118231401 | (18984 2 20883 2) | | 2.76 | 4.54E-07 | 1.02 | 0.79 | 0.81 |
| rs9846736xrs7280997 | 3 | 31586782 | 21 | 36580379 | (31024 3 172553 3) | | 2.07 | 4.6834E-07 | 1.3 | 1.32 | 1.73 |
| rs707025xrs189900 | 2 | 154896769 | 21 | 23322128 | (23082 3 171384 2) | | 0.43 | 4.8064E-07 | 0.84 | 0.94 | 0.79 |
| rs3807660xrs11624725 | 7 | 77656088 | 14 | 89783362 | (82656 1 145706 3) | | 3.16 | 4.8565E-07 | 0.81 | 0.92 | 0.75 |
| rs2292518xrs10758941 | 3 | 112928236 | 9 | 8126797 | (35700 2 98556 2) | | 1.97 | 4.9326E-07 | 0.82 | 0.81 | 0.67 |
| rs10141804xrs9959583 | 14 | 70318983 | 18 | 20993778 | (144417 2 160672 2) | | 2.07 | 4.9582E-07 | 0.97 | 1.25 | 1.23 |
| rs10493335xrs6934412 | 1 | 63354702 | 6 | 85119973 | (3060 3 71690 3) | | 2.14 | 5.2221E-07 | 0.93 | 0.87 | 0.81 |
| rs1383421xrs10501659 | 4 | 80154502 | 11 | 87513536 | (46300 3 121106 2) | | 1.83 | 5.5E-07 | 0.9 | 0.98 | 0.89 |
| rs4737291xrs1474844 | 8 | 70924348 | 11 | 32266552 | (92494 2 118205 3) | | 2.12 | 5.733E-07 | 0.72 | 0.78 | 0.56 |
| rs10800533xrs4146606 | 1 | 160683253 | 5 | 34539764 | (7510 3 56254 3) | | 2.08 | 6.007E-07 | 0.97 | 0.78 | 0.76 |
| rs10516147xrs2804869 | 5 | 178027090 | 10 | 33856365 | (65492 3 108943 1) | | 2.79 | 6.2615E-07 | 0.97 | 0.65 | 0.63 |
| rs2181011xrs11016976 | 6 | 70896157 | 10 | 131540391 | (70668 2 115468 3) | | 0.52 | 6.2615E-07 | 1.06 | 0.88 | 0.93 |
| rs1015340xrs10263677 | 6 | 144077050 | 7 | 40578775 | (75645 1 80897 2) | | 3.65 | 6.3597E-07 | 0.66 | 1.05 | 0.69 |
| rs1468244xrs10954969 | 7 | 11431856 | 8 | 34724264 | (78463 1 90642 2) | | 4.17 | 6.4261E-07 | 0.86 | 0.94 | 0.82 |
| rs193803xrs7096673 | 7 | 105394028 | 10 | 88522232 | (84313 2 112495 2) | | 3.9 | 6.4595E-07 | 0.8 | 1.16 | 0.94 |
| rs7774757xrs2369548 | 6 | 155417681 | 8 | 80044352 | (76527 3 93141 2) | | 0.4 | 6.5269E-07 | 1.11 | 1.65 | 1.84 |
| rs685875xrs7856164 | 5 | 16709506 | 9 | 110574103 | (55159 3 104322 3) | | 1.82 | 6.595E-07 | 0.85 | 0.88 | 0.75 |
| rs10957988xrs2173202 | 8 | 81983149 | 17 | 36488761 | (93240 3 157472 1) | | 3.05 | 6.6293E-07 | 0.84 | 0.77 | 0.65 |
| rs17375557xrs9564316 | 12 | 95442660 | 13 | 33884770 | (131199 2 134738 3) | | 2.17 | 6.6638E-07 | 1.16 | 1.05 | 1.23 |
| rs1845040xrs8035127 | 8 | 19612672 | 15 | 83929154 | (89582 3 150020 3) | | 1.89 | 6.6638E-07 | 0.8 | 1.33 | 1.07 |
| rs685875xrs10145565 | 5 | 16709506 | 14 | 68612977 | (55159 3 144317 3) | | 2 | 6.7333E-07 | 1.2 | 0.97 | 1.17 |
| rs9660543xrs10123421 | 1 | 227349555 | 9 | 76903099 | (11989 3 101780 3) | | 1.79 | 6.7683E-07 | 1.15 | 0.78 | 0.9 |
| rs1220445xrs2957761 | 6 | 91712833 | 11 | 24098510 | (72142 2 117558 3) | | 1.84 | 6.7683E-07 | 0.97 | 0.98 | 0.96 |
| rs4380636xrs10501659 | 5 | 93203859 | 11 | 87513536 | (59729 3 121106 2) | | 1.82 | 6.7683E-07 | 0.99 | 0.82 | 0.82 |
| rs17191351xrs4146472 | 15 | 58418368 | 15 | 62087994 | (148862 2 149127 2) | | 3.72 | 6.8035E-07 | 1.1 | 0.89 | 0.98 |
| rs3770542xrs1023890 | 2 | 216555396 | 4 | 118920894 | (26761 3 48814 1) | | 2.28 | 7.666E-07 | 0.86 | 0.87 | 0.75 |
| rs9873216xrs10973109 | 3 | 64094238 | 9 | 36851407 | (32836 1 101142 3) | | 0.38 | 7.8269E-07 | 1.32 | 1.39 | 1.84 |
| rs565394xrs1227921 | 2 | 21259094 | 2 | 162400446 | (15115 1 23544 3) | | 0.24 | 8.4169E-07 | 1.23 | 1.08 | 1.34 |
| rs7664129xrs10506984 | 4 | 159189132 | 12 | 89217396 | (51360 2 130701 3) | | 2.31 | 9.534E-07 | 0.69 | 0.83 | 0.57 |
| rs1015887xrs10263677 | 6 | 144077451 | 7 | 40578775 | (75647 1 80897 2) | | 3.76 | 9.5837E-07 | 0.79 | 1.07 | 0.84 |
| rs6463813xrs2896252 | 7 | 8476768 | 14 | 93497784 | (78178 2 145911 1) | | 2.84 | 9.7849E-07 | 0.96 | 0.75 | 0.72 |
| rs1295212xrs17118166 | 5 | 154965952 | 5 | 155010282 | (63877 2 63884 2) | | 6.84 | 9.887E-07 | 1.05 | 0.74 | 0.78 |
| rs1728232xrs11218350 | 1 | 113366167 | 11 | 120957861 | (6450 3 123677 2) | | 2.48 | 9.9903E-07 | 0.91 | 1.17 | 1.07 |
| rs2205848xrs11999298 | 1 | 167942917 | 9 | 36884151 | (8170 2 101146 2) | | 3.57 | 1.0307E-06 | 1 | 0.6 | 0.6 |
| rs9873216xrs17594878 | 3 | 64094238 | 8 | 137855619 | (32836 1 97145 3) | | 0.44 | 1.0578E-06 | 1.79 | 1.43 | 2.56 |
| rs6908632xrs730520 | 6 | 17505095 | 20 | 7635252 | (67130 3 167131 2) | | 0.5 | 1.097E-06 | 1.18 | 1.03 | 1.22 |
| rs6706469xrs1930551 | 2 | 240059548 | 9 | 104380162 | (28313 3 103761 2) | | 2.95 | 1.1317E-06 | 0.87 | 0.88 | 0.77 |
| rs6445058xrs2833822 | 3 | 173498694 | 21 | 32726508 | (39431 2 172288 2) | | 1.93 | 1.3022E-06 | 0.65 | 0.92 | 0.6 |
| rs2731402xrs17375557 | 12 | 59992544 | 12 | 95442660 | (128713 3 131199 2) | | 2.2 | 1.3434E-06 | 0.76 | 1.04 | 0.79 |
| rs10170310xrs1436958 | 2 | 138995392 | 15 | 60126089 | (22083 3 149013 1) | | 0.25 | 1.5459E-06 | 1.16 | 1.15 | 1.34 |
| rs880385xrs7731633 | 1 | 55592687 | 5 | 85586892 | (2409 1 59387 3) | | 3.1 | 1.5784E-06 | 0.98 | 0.92 | 0.91 |
| rs12595616xrs10852877 | 15 | 89364517 | 17 | 6230343 | (150364 2 156261 2) | | 2.34 | 1.7065E-06 | 0.92 | 0.75 | 0.7 |
| rs11795150xrs11002539 | 9 | 16095961 | 10 | 79841011 | (99453 1 111809 3) | | 0.34 | 1.7334E-06 | 0.99 | 1.11 | 1.1 |
| rs931882xrs12961718 | 12 | 59868562 | 18 | 65238984 | (128696 1 163903 2) | | 3.85 | 1.7424E-06 | 1.11 | 0.89 | 0.99 |
| rs2008776xrs17738626 | 2 | 118997264 | 15 | 60613489 | (20927 1 149050 3) | | 2.05 | 1.7424E-06 | 0.61 | 0.77 | 0.47 |
| rs4973006xrs10040249 | 2 | 228455120 | 5 | 110625639 | (27608 1 60817 3) | | 0.42 | 1.8165E-06 | 1.57 | 1.07 | 1.69 |
| rs3778248xrs10990113 | 6 | 165674451 | 9 | 104268870 | (77384 3 103749 2) | | 2.66 | 1.9135E-06 | 0.81 | 0.72 | 0.59 |
| rs2249816xrs7131306 | 10 | 62917801 | 11 | 107830026 | (110744 2 122814 2) | | 2.35 | 1.9537E-06 | 0.77 | 0.84 | 0.65 |
| rs2085672xrs1374482 | 6 | 153903143 | 13 | 57054311 | (76404 3 136344 3) | | 0.52 | 2.1234E-06 | 1.21 | 0.79 | 0.96 |
| rs11727321xrs2041001 | 4 | 43344913 | 7 | 107870335 | (44094 3 84489 2) | | 2.85 | 2.1681E-06 | 1.18 | 0.71 | 0.84 |
| rs6738950xrs10104759 | 2 | 227755364 | 8 | 35423940 | (27556 3 90691 3) | | 1.79 | 2.2137E-06 | 0.67 | 0.5 | 0.34 |
| rs9472419xrs7488933 | 6 | 12826487 | 12 | 8902779 | (66766 2 125305 3) | | 0.4 | 2.2253E-06 | 1.17 | 1.44 | 1.69 |
| rs6967565xrs7161259 | 7 | 8096253 | 14 | 91214828 | (78135 3 145780 1) | | 0.29 | 2.2603E-06 | 1.11 | 1.52 | 1.69 |
| rs11718847xrs10491590 | 3 | 43026662 | 9 | 728940 | (31651 2 97687 2) | | 4.91 | 2.3812E-06 | 0.83 | 0.75 | 0.63 |
| rs16831315xrs9554679 | 2 | 135541544 | 13 | 99787063 | (21888 2 139767 3) | | 2.43 | 2.4061E-06 | 0.85 | 0.84 | 0.72 |
| rs7045215xrs2306743 | 9 | 104739847 | 16 | 34649292 | (103804 3 152989 2) | | 0.27 | 2.4313E-06 | 1.04 | 1.12 | 1.17 |
| rs542972xrs1461371 | 2 | 239032770 | 11 | 26351264 | (28272 2 117812 3) | | 0.52 | 2.5216E-06 | 1.42 | 1.47 | 2.09 |
| rs1329393xrs4408437 | 9 | 97358747 | 13 | 57133947 | (103286 3 136349 2) | | 1.98 | 2.5613E-06 | 1.14 | 1.02 | 1.17 |
| rs7656584xrs7808652 | 4 | 137628031 | 7 | 88881333 | (49916 2 83490 2) | | 0.4 | 2.6152E-06 | 1.37 | 0.98 | 1.35 |
| rs9368394xrs16938580 | 6 | 22132981 | 8 | 74249204 | (67506 2 92743 3) | | 0.23 | 2.7123E-06 | 1.65 | 1.09 | 1.81 |
| rs782103xrs7228414 | 12 | 57122898 | 18 | 30888632 | (128494 1 161338 2) | | 4.62 | 2.8278E-06 | 0.94 | 0.85 | 0.8 |
| rs11892271xrs9600236 | 2 | 52237202 | 13 | 73603200 | (17273 2 137664 2) | | 3.75 | 2.9946E-06 | 0.91 | 0.77 | 0.7 |
| rs7759835xrs1845040 | 6 | 9529407 | 8 | 19612672 | (66469 3 89582 3) | | 1.96 | 3.0418E-06 | 0.71 | 0.88 | 0.63 |
| rs416101xrs16921774 | 6 | 95090722 | 9 | 104336206 | (72408 2 103756 2) | | 3.83 | 3.2892E-06 | 0.84 | 1.19 | 1.01 |
| rs2891152xrs10990113 | 9 | 20898750 | 9 | 104268870 | (99947 3 103749 2) | | 3.48 | 3.3064E-06 | 0.86 | 1.02 | 0.89 |
| rs2707469xrs9299582 | 7 | 120764122 | 10 | 51974286 | (85143 2 109697 1) | | 3.45 | 3.4834E-06 | 0.86 | 0.8 | 0.69 |
| rs2800708xrs1665571 | 6 | 127479310 | 10 | 80081635 | (74590 3 111833 2) | | 2.7 | 3.6698E-06 | 0.87 | 1 | 0.87 |
| rs4373539xrs6538271 | 8 | 5063717 | 12 | 90335642 | (88092 1 130780 3) | | 4.04 | 3.8866E-06 | 0.53 | 0.89 | 0.47 |
| rs1764844xrs1836872 | 1 | 217309442 | 16 | 62621225 | (11388 3 154035 2) | | 2.5 | 4.641E-06 | 0.75 | 0.7 | 0.53 |
| rs12695382xrs912044 | 3 | 120430861 | 13 | 38962446 | (36227 2 135178 2) | | 2.54 | 4.8643E-06 | 0.84 | 0.7 | 0.6 |
| rs6602686xrs7240074 | 10 | 13904269 | 18 | 25336210 | (107320 1 160958 2) | | 2.57 | 5.125E-06 | 0.53 | 0.83 | 0.44 |
| rs4292660xrs6581677 | 8 | 99865472 | 12 | 65056646 | (94317 3 129022 1) | | 2.67 | 5.3997E-06 | 0.99 | 0.77 | 0.76 |

SNP: single nucleotide polymorphism, CHR: Chromosome, BP: Base pair position, HFCC: hypothesis free clinical cloning, HFCC OR: odds ratio (calculated comparing selected diplotype versus the rest of genotypic combinations), p (HFCC): p value associated with HFCC OR (1 df), OR (M1): univariate effect size of marker 1 of HFCC diplotype, OR (M2): univariate effect size of marker 2 of HFCC diplotype, Mult: expected effect size of HFCC diplotype under linear (multiplicative) models.

*According to UCSC genome browser (NCBI36/hg18) and dbSNP build 130.
