# Supplementary material for: A Colorectal Cancer Susceptibility New Variant at 4q26 in the Spanish Population Identified by Genome-Wide Association Analysis
Source: PLoS One. 2014 Jun 30;9(6):e101178. doi: 10.1371/journal.pone.0101178 (PMC4076321; doi:10.1371/journal.pone.0101178)
Supplement: Table S3 — SNPs included in the phase II and meta-analysis results. (DOC) [file pone.0101178.s006.doc]

**Table S3.** SNPs included in the phase II and meta-analysis results.

|  |  |  |  |  |  |  |  |  |  |  | NXC-GWAS | | NXC-VAL | |
| --- | --- | --- | --- | --- | --- | --- | --- | --- | --- | --- | --- | --- | --- | --- |
| CHR | SNPs OPA | BP** | A1 | A2 | P | P(R) | OR | OR(R) | Q | I | P | OR | P | OR |
| 4 | rs10446758 | 149667035 | A | G | 0.0000162 | 0.2161 | 0.7802 | 0.7699 | 0.0002 | 92.55 | 1.73E-08 | 0.6225 | 0.5159 | 0.9501 |
| 16 | rs4887855 | 74989899 | T | C | 0.0009276 | 0.2635 | 0.6893 | 0.5834 | 0.0001 | 93.71 | 8.27E-08 | 0.3562 | 0.6159 | 0.9342 |
| 15 | rs7171889 | 92377186 | C | A | 0.00000994 | 0.154 | 1.5943 | 1.5671 | 0.0029 | 88.74 | 8.53E-08 | 2.143 | 0.392 | 1.141 |
| 20 | rs16986484 | 24276308 | C | T | 0.004728 | 0.5035 | 1.3073 | 1.2715 | 0.0002 | 92.97 | 2.89E-06 | 1.817 | 0.3877 | 0.8861 |
| 1 | rs3014578 | 239698430 | C | T | 0.0001806 | 0.1575 | 1.518 | 1.5242 | 0.0075 | 86.02 | 4.00E-06 | 2.055 | 0.4284 | 1.132 |
| 22 | rs242896 | 32765815 | G | C | 0.0008968 | 0.281 | 1.2082 | 1.2161 | 0.0015 | 90.13 | 4.20E-06 | 1.459 | 0.8509 | 1.015 |
| 10 | rs17091953 | 116253478 | G | A | 0.0004795 | 0.2181 | 1.2819 | 1.2902 | 0.0037 | 88.16 | 5.66E-06 | 1.588 | 0.6221 | 1.05 |
| 2 | rs559113 | 169411175 | A | T | 0.0009252 | 0.2364 | 0.826 | 0.8188 | 0.0035 | 88.28 | 1.06E-05 | 0.6908 | 0.6849 | 0.9683 |
| 5 | rs16879116 | 7847503 | G | A | 0.0005454 | 0.196 | 0.8152 | 0.8088 | 0.0055 | 87 | 1.09E-05 | 0.6855 | 0.5443 | 0.9519 |
| 5 | rs7734355 | 102775829 | C | T | 0.007519 | 0.4334 | 1.4135 | 1.4123 | 0.0007 | 91.37 | 1.13E-05 | 2.194 | 0.6023 | 0.9089 |
| 12 | rs1477102 | 95526343 | G | T | 0.0000165 | 0.02066 | 0.7815 | 0.7786 | 0.0592 | 71.92 | 1.38E-05 | 0.6978 | 0.06951 | 0.8663 |
| 11 | rs2403583 | 20334676 | C | A | 0.0000137 | 0.01504 | 1.3584 | 1.3632 | 0.0706 | 69.41 | 1.45E-05 | 1.551 | 0.05934 | 1.202 |
| 22 | rs6009047 | 45683534 | T | C | 0.0002611 | 0.12 | 0.8028 | 0.7967 | 0.0153 | 83 | 1.90E-05 | 0.6873 | 0.3167 | 0.9207 |
| 12 | rs1922397* | 80507780 | T | C | 0.0001931 | 0.09058 | 0.7075 | 0.6897 | 0.0192 | 81.77 | 2.05E-05 | 0.5506 | 0.1991 | 0.8542 |
| 8 | rs7838116 | 40048312 | A | G | 0.000191 | 0.08381 | 0.684 | 0.6744 | 0.0257 | 79.92 | 2.38E-05 | 0.5351 | 0.2209 | 0.844 |
| 2 | rs7578749 | 148038283 | A | G | 0.0007198 | 0.1761 | 0.7952 | 0.7891 | 0.0099 | 84.98 | 2.50E-05 | 0.6614 | 0.4994 | 0.9388 |
| 1 | rs4649259 | 232007684 | G | A | 0.001184 | 0.2242 | 1.2199 | 1.2239 | 0.0067 | 86.4 | 2.52E-05 | 1.446 | 0.6691 | 1.037 |
| 10 | rs17091955 | 116253976 | T | C | 0.001785 | 0.247 | 1.2515 | 1.26 | 0.0055 | 87.04 | 2.83E-05 | 1.54 | 0.7458 | 1.033 |
| 5 | rs588367 | 16709570 | A | G | 0.0000139 | 0.007262 | 0.7695 | 0.7669 | 0.1015 | 62.7 | 2.85E-05 | 0.6933 | 0.04218 | 0.8449 |
| 22 | rs5769234 | 45683168 | C | G | 0.0009625 | 0.1716 | 0.8205 | 0.8143 | 0.0123 | 84.04 | 4.25E-05 | 0.6997 | 0.4919 | 0.945 |
| 2 | rs707025* | 154896769 | A | C | 0.01756 | 0.4604 | 1.1469 | 1.1547 | 0.0007 | 91.21 | 4.41E-05 | 1.404 | 0.53 | 0.9509 |
| 1 | rs1359414 | 111963158 | C | T | 0.0009125 | 0.1614 | 1.242 | 1.2469 | 0.0159 | 82.79 | 5.17E-05 | 1.461 | 0.4821 | 1.066 |
| 1 | rs6429174 | 238239018 | G | T | 0.07371 | 0.6362 | 1.1132 | 1.1156 | 0.0001 | 93.27 | 6.16E-05 | 1.406 | 0.1491 | 0.8854 |
| 9 | rs7039568 | 104361604 | T | C | 0.0003042 | 0.06304 | 1.5134 | 1.5422 | 0.0434 | 75.5 | 6.90E-05 | 1.959 | 0.1808 | 1.229 |
| 5 | rs12519633 | 121967053 | T | C | 0.002279 | 0.2017 | 1.3317 | 1.36 | 0.0107 | 84.64 | 7.00E-05 | 1.737 | 0.5775 | 1.073 |
| 9 | rs10990158 | 104335927 | T | A | 0.000107 | 0.02387 | 1.5395 | 1.5585 | 0.079 | 67.6 | 7.40E-05 | 1.908 | 0.09212 | 1.288 |
| 5 | rs428263 | 16711495 | T | C | 0.0000261 | 0.003224 | 0.777 | 0.7756 | 0.1507 | 51.59 | 7.66E-05 | 0.7102 | 0.04125 | 0.844 |
| 1 | rs17666678 | 215844513 | T | C | 0.001331 | 0.1701 | 1.2526 | 1.2552 | 0.0182 | 82.06 | 7.94E-05 | 1.482 | 0.5287 | 1.064 |
| 7 | rs6943487 | 12711855 | C | G | 0.002223 | 0.2116 | 0.8193 | 0.8151 | 0.0121 | 84.13 | 8.14E-05 | 0.6914 | 0.6446 | 0.9591 |
| 9 | rs1930551* | 104380162 | T | G | 0.000165 | 0.03154 | 1.5413 | 1.5634 | 0.0716 | 69.2 | 8.67E-05 | 1.937 | 0.113 | 1.278 |
| 15 | rs4842907 | 84239094 | A | C | 0.004055 | 0.2423 | 0.7782 | 0.7617 | 0.008 | 85.78 | 8.73E-05 | 0.6014 | 0.7147 | 0.958 |
| 5 | rs876095 | 16709803 | T | C | 0.00003 | 0.00312 | 0.7787 | 0.7772 | 0.1552 | 50.51 | 8.81E-05 | 0.7124 | 0.04218 | 0.8449 |
| 7 | rs4722778 | 28278588 | G | C | 0.001203 | 0.1482 | 0.8109 | 0.8058 | 0.0212 | 81.16 | 9.24E-05 | 0.693 | 0.4458 | 0.9342 |
| 12 | rs2710901 | 77433629 | G | A | 0.04771 | 0.5981 | 1.1555 | 1.146 | 0.0004 | 92.01 | 9.77E-05 | 1.483 | 0.2419 | 0.8843 |
| 3 | rs4373099 | 54254022 | T | C | 0.004155 | 0.2583 | 1.1834 | 1.189 | 0.0092 | 85.27 | 0.0001069 | 1.387 | 0.8021 | 1.021 |
| 5 | rs13354207 | 110179690 | C | G | 0.04783 | 0.5428 | 1.1421 | 1.1518 | 0.0005 | 91.63 | 0.0001083 | 1.454 | 0.3331 | 0.9138 |
| 20 | rs1810636 | 2602925 | T | G | 0.03015 | 0.4687 | 0.8803 | 0.8701 | 0.0011 | 90.57 | 0.0001105 | 0.7173 | 0.521 | 1.053 |
| 18 | rs4477825 | 63210306 | C | G | 0.01141 | 0.3678 | 1.1546 | 1.1616 | 0.0034 | 88.32 | 0.0001112 | 1.373 | 0.8416 | 0.9844 |
| 9 | rs16921774* | 104336206 | T | C | 0.0003542 | 0.05051 | 1.4951 | 1.5101 | 0.0617 | 71.35 | 0.0001148 | 1.872 | 0.1824 | 1.228 |
| 9 | rs10990136 | 104298657 | T | C | 0.0004071 | 0.05562 | 1.5041 | 1.5211 | 0.0583 | 72.1 | 0.0001182 | 1.902 | 0.1935 | 1.227 |
| 3 | rs794184 | 4426994 | C | T | 0.04757 | 0.5249 | 0.8897 | 0.8798 | 0.0006 | 91.4 | 0.000119 | 0.7185 | 0.3722 | 1.075 |
| 12 | rs1521386* | 77418817 | G | A | 0.06195 | 0.6223 | 1.1453 | 1.1371 | 0.0003 | 92.22 | 0.000119 | 1.475 | 0.2026 | 0.8755 |
| 8 | rs13273088 | 70656528 | G | A | 0.0001351 | 0.01971 | 1.3046 | 1.3085 | 0.0981 | 63.45 | 0.0001194 | 1.471 | 0.1075 | 1.168 |
| 4 | rs7657701 | 182529026 | C | T | 0.1158 | 0.5711 | 0.8505 | 0.8064 | 0.0003 | 92.48 | 0.0001247 | 0.5491 | 0.2378 | 1.174 |
| 4 | rs3987* | 118978503 | C | T | 4.02E-08 | 4.02E-08 | 1.3677 | 1.3677 | 0.949 | 0 | 0.0001256 | 1.373 | 8.14E-05 | 1.363 |
| 4 | rs1023890* | 118920894 | A | G | 5.61E-08 | 5.61E-08 | 1.364 | 1.364 | 0.9032 | 0 | 0.0001274 | 1.374 | 0.0001141 | 1.355 |
| 2 | rs6730095 | 77151964 | A | C | 0.008352 | 0.3341 | 1.1775 | 1.1792 | 0.0059 | 86.83 | 0.0001318 | 1.399 | 0.9485 | 0.9944 |
| 1 | rs9660543* | 227349555 | T | C | 0.004835 | 0.2503 | 0.8023 | 0.7942 | 0.0105 | 84.73 | 0.0001376 | 0.6488 | 0.7669 | 0.9687 |
| 20 | rs10485515* | 14797853 | T | C | 0.01986 | 0.3956 | 0.8326 | 0.8175 | 0.0027 | 88.92 | 0.0001397 | 0.6434 | 0.7516 | 1.034 |
| 12 | rs4842316 | 78498696 | G | A | 0.01503 | 0.387 | 1.1523 | 1.1592 | 0.0034 | 88.32 | 1.43E-04 | 1.376 | 0.7834 | 0.978 |
| 1 | rs16859609 | 232000522 | G | A | 0.002996 | 0.2077 | 1.2016 | 1.2054 | 0.0166 | 82.57 | 1.49E-04 | 1.399 | 0.6493 | 1.04 |
| 20 | rs1998087 | 14764382 | A | C | 0.02035 | 0.3927 | 0.8351 | 0.8193 | 0.0028 | 88.81 | 1.50E-04 | 0.6473 | 0.7623 | 1.032 |
| 7 | rs17160621 | 138268222 | T | C | 0.008748 | 0.3424 | 1.2784 | 1.2687 | 0.0075 | 86.01 | 1.64E-04 | 1.628 | 0.9178 | 0.9862 |
| 3 | rs4594610* | 989666 | T | C | 0.0101 | 0.2867 | 0.7457 | 0.7195 | 0.0073 | 86.12 | 1.65E-04 | 0.5252 | 0.864 | 0.9744 |
| 9 | rs6478690 | 126797131 | C | T | 0.003733 | 0.218 | 1.1814 | 1.1876 | 0.0153 | 83.01 | 1.71E-04 | 1.367 | 0.6734 | 1.034 |
| 4 | rs2881373 | 159312083 | T | C | 0.00000686 | 0.00000686 | 1.3928 | 1.3928 | 0.3512 | 0 | 1.71E-04 | 1.5 | 0.00759 | 1.307 |
| 11 | rs538645 | 118216279 | G | A | 0.02333 | 0.4355 | 1.1671 | 1.1732 | 0.0026 | 88.94 | 1.78E-04 | 1.441 | 0.6394 | 0.9565 |
| 4 | rs12503362 | 14452104 | T | C | 0.0008626 | 0.07836 | 1.4117 | 1.4184 | 0.0551 | 72.81 | 1.89E-04 | 1.733 | 0.2874 | 1.165 |
| 5 | rs2416248 | 110206705 | C | T | 0.08616 | 0.6013 | 1.1193 | 1.1284 | 0.0004 | 91.92 | 1.89E-04 | 1.423 | 0.2285 | 0.8961 |
| 4 | rs1459528 | 118969796 | G | A | 4.57E-07 | 4.57E-07 | 1.3322 | 1.3322 | 0.7469 | 0 | 1.96E-04 | 1.358 | 0.00062 | 1.309 |
| 10 | rs941853 | 116189165 | A | G | 0.0009047 | 0.08198 | 0.7649 | 0.764 | 0.0553 | 72.77 | 2.04E-04 | 0.6542 | 0.3117 | 0.8915 |
| 7 | rs1029621 | 40832234 | A | G | 0.003886 | 0.2064 | 1.2072 | 1.2152 | 0.0182 | 82.08 | 2.07E-04 | 1.42 | 0.6411 | 1.043 |
| 13 | rs9540846 | 66110941 | A | T | 0.0008921 | 0.07614 | 0.7098 | 0.702 | 0.0537 | 73.13 | 2.07E-04 | 0.5726 | 0.2595 | 0.8535 |
| 3 | rs7645545 | 54392362 | T | C | 0.1222 | 0.657 | 1.0988 | 1.1045 | 0.0002 | 92.57 | 2.08E-04 | 1.382 | 0.1454 | 0.8834 |
| 3 | rs421653* | 32486654 | T | G | 0.02107 | 0.4126 | 1.1422 | 1.1478 | 0.0035 | 88.25 | 2.10E-04 | 1.359 | 0.7109 | 0.9707 |
| 13 | rs354789 | 57024535 | T | G | 0.0005687 | 0.0543 | 1.2775 | 1.2866 | 0.0662 | 70.38 | 2.16E-04 | 1.471 | 0.2008 | 1.132 |
| 3 | rs3774230 | 181004332 | C | T | 0.00139 | 0.1116 | 0.8204 | 0.8168 | 0.0401 | 76.26 | 2.17E-04 | 0.7182 | 0.3712 | 0.9263 |
| 9 | rs7024470 | 104361506 | G | A | 0.000523 | 0.04462 | 1.4873 | 1.5083 | 0.075 | 68.45 | 2.21E-04 | 1.863 | 0.1679 | 1.237 |
| 8 | rs12545053 | 65236159 | C | T | 0.003012 | 0.1776 | 0.8393 | 0.835 | 0.0237 | 80.46 | 2.25E-04 | 0.7295 | 0.5581 | 0.9533 |
| 19 | rs17239559 | 59398234 | C | T | 0.0004549 | 0.04073 | 0.8162 | 0.8137 | 0.0823 | 66.89 | 2.31E-04 | 0.7346 | 0.182 | 0.8986 |
| 13 | rs1283145 | 100234116 | C | T | 0.005201 | 0.2184 | 0.7351 | 0.7251 | 0.0179 | 82.15 | 2.33E-04 | 0.5567 | 0.6754 | 0.9387 |
| 3 | rs4234541 | 17157701 | C | T | 0.3673 | 0.8068 | 1.0526 | 1.0608 | 0 | 94.44 | 2.35E-04 | 1.351 | 0.02144 | 0.8337 |
| 1 | rs550437 | 104121003 | G | C | 0.01478 | 0.3454 | 0.8625 | 0.8543 | 0.0061 | 86.71 | 2.38E-04 | 0.722 | 0.9202 | 1.008 |
| 8 | rs1457461 | 135167438 | C | T | 0.002212 | 0.1439 | 1.2491 | 1.2555 | 0.0324 | 78.16 | 2.39E-04 | 1.469 | 0.4656 | 1.076 |
| 4 | rs1870481 | 118882468 | T | C | 1.23E-07 | 1.23E-07 | 1.3515 | 1.3515 | 0.9948 | 0 | 2.40E-04 | 1.351 | 0.0001359 | 1.352 |
| 3 | rs1473348 | 54384035 | C | T | 0.1006 | 0.6237 | 1.1061 | 1.1119 | 0.0004 | 91.93 | 2.41E-04 | 1.381 | 0.2006 | 0.8961 |
| 1 | rs4656349 | 160316448 | G | A | 0.0003576 | 0.0291 | 1.2319 | 1.2353 | 0.0978 | 63.52 | 2.44E-04 | 1.363 | 0.1513 | 1.123 |
| 2 | rs2008776* | 118997264 | G | A | 0.02862 | 0.4383 | 1.1324 | 1.1399 | 0.003 | 88.68 | 2.46E-04 | 1.351 | 0.6365 | 0.9635 |
| 5 | rs6888588 | 110225865 | G | A | 0.0998 | 0.6153 | 1.1141 | 1.1221 | 0.0005 | 91.79 | 2.50E-04 | 1.412 | 0.2139 | 0.8928 |
| 12 | rs931586 | 53360703 | G | C | 0.0004728 | 0.03733 | 0.7947 | 0.7921 | 0.0889 | 65.46 | 2.58E-04 | 0.707 | 0.1765 | 0.8844 |
| 1 | rs2646249 | 104110655 | C | A | 0.01547 | 0.3511 | 0.864 | 0.8572 | 0.0063 | 86.61 | 2.58E-04 | 0.7257 | 0.9047 | 1.01 |
| 22 | rs2255957 | 40571318 | A | G | 0.2894 | 0.714 | 0.9158 | 0.8868 | 0.0001 | 93.51 | 2.66E-04 | 0.6376 | 0.06611 | 1.228 |
| 7 | rs2041001* | 107870335 | G | A | 0.0006956 | 0.04455 | 1.5209 | 1.5313 | 0.0865 | 65.96 | 2.80E-04 | 1.9 | 0.2026 | 1.243 |
| 13 | rs1374482* | 57054311 | T | C | 0.001739 | 0.1064 | 1.2339 | 1.2431 | 0.0451 | 75.09 | 3.09E-04 | 1.426 | 0.3524 | 1.089 |
| 3 | rs17021431* | 84427280 | T | G | 0.01596 | 0.3781 | 1.2312 | 1.2233 | 0.0081 | 85.74 | 3.30E-04 | 1.536 | 0.8204 | 0.9722 |
| 12 | rs2710922 | 77423482 | G | A | 0.01935 | 0.4124 | 1.2525 | 1.2366 | 0.0072 | 86.15 | 3.61E-04 | 1.599 | 0.7279 | 0.9524 |
| 15 | rs7183242 | 64883712 | G | A | 0.02306 | 0.3629 | 0.8623 | 0.8499 | 0.0063 | 86.58 | 3.74E-04 | 0.7092 | 0.8754 | 1.014 |
| 7 | rs17717216* | 28448949 | C | T | 0.03232 | 0.4195 | 0.8732 | 0.8639 | 0.0043 | 87.73 | 3.78E-04 | 0.7197 | 0.6963 | 1.034 |
| 4 | rs2169059 | 118926638 | A | C | 2.04E-07 | 2.04E-07 | 1.3442 | 1.3442 | 0.9375 | 0 | 3.90E-04 | 1.338 | 0.0001412 | 1.35 |
| 10 | rs11016976* | 131540391 | C | T | 0.0005038 | 0.02384 | 1.2734 | 1.2758 | 0.1209 | 58.43 | 3.90E-04 | 1.423 | 0.1573 | 1.147 |
| 19 | rs8111948* | 33517335 | G | A | 0.005088 | 0.1783 | 1.1732 | 1.1776 | 0.0334 | 77.91 | 5.00E-04 | 1.331 | 0.5892 | 1.044 |
| 9 | rs6474838* | 14454254 | G | C | 0.001094 | 0.04835 | 1.2319 | 1.2358 | 0.0935 | 64.44 | 5.11E-04 | 1.378 | 0.228 | 1.112 |
| 8 | rs2975696* | 10150116 | A | C | 0.1581 | 0.6317 | 0.9159 | 0.9028 | 0.0006 | 91.44 | 5.18E-04 | 0.7283 | 0.1965 | 1.116 |
| 8 | rs6988293* | 121303479 | A | G | 0.05749 | 0.4964 | 1.1138 | 1.1206 | 0.0032 | 88.5 | 5.76E-04 | 1.326 | 0.502 | 0.9486 |
| 7 | rs5014691 | 14817686 | A | G | 0.00367 | 0.1185 | 1.2072 | 1.2133 | 0.0563 | 72.54 | 6.97E-04 | 1.376 | 0.425 | 1.074 |
| 1 | rs10157868* | 96049209 | A | G | 0.001707 | 0.04783 | 1.2209 | 1.2241 | 0.1085 | 61.17 | 8.48E-04 | 1.358 | 0.2467 | 1.107 |
| 6 | rs221752* | 165779431 | T | A | 0.0004178 | 0.003772 | 0.7957 | 0.7946 | 0.2207 | 33.32 | 8.90E-04 | 0.7319 | 0.08585 | 0.8579 |
| 21 | rs7280997* | 36580379 | T | C | 0.00309 | 0.08502 | 0.8436 | 0.8408 | 0.0802 | 67.33 | 9.31E-04 | 0.7591 | 0.3495 | 0.9284 |
| 3 | rs9873216* | 64094238 | G | A | 0.01457 | 0.2608 | 0.8706 | 0.867 | 0.0253 | 80.01 | 9.38E-04 | 0.7628 | 0.83 | 0.9833 |
| 2 | rs10497616* | 183986209 | T | C | 0.2019 | 0.6406 | 0.913 | 0.8963 | 0.001 | 90.69 | 1.01E-03 | 0.7076 | 0.2029 | 1.131 |
| 1 | rs10493335* | 63354702 | A | G | 0.00874 | 0.1779 | 0.857 | 0.8521 | 0.0439 | 75.38 | 1.09E-03 | 0.7552 | 0.5932 | 0.9578 |
| 1 | rs7518650* | 215538497 | G | A | 0.09412 | 0.5143 | 0.8497 | 0.8337 | 0.0043 | 87.75 | 1.13E-03 | 0.6291 | 0.477 | 1.099 |
| 3 | rs4687889* | 119020129 | G | A | 0.021 | 0.292 | 0.8771 | 0.8731 | 0.0235 | 80.5 | 1.22E-03 | 0.7668 | 0.9181 | 0.992 |
| 18 | rs4939756* | 43977260 | G | C | 0.02112 | 0.2958 | 1.1839 | 1.1865 | 0.0255 | 79.95 | 1.29E-03 | 1.398 | 0.9371 | 1.008 |
| 8 | rs2588223* | 17431383 | T | A | 0.1507 | 0.6172 | 1.0854 | 1.0917 | 0.0021 | 89.44 | 1.30E-03 | 1.302 | 0.2712 | 0.9166 |
| 14 | rs8012146* | 62215852 | A | C | 0.1378 | 0.5589 | 0.9075 | 0.8924 | 0.003 | 88.64 | 1.37E-03 | 0.7328 | 0.3696 | 1.082 |
| 7 | rs10263677* | 40578775 | G | A | 0.0261 | 0.3215 | 1.2227 | 1.2239 | 0.0241 | 80.34 | 1.44E-03 | 1.501 | 0.9909 | 0.9985 |
| 4 | rs7664129* | 159189132 | C | A | 0.000197 | 0.000197 | 1.3596 | 1.3596 | 0.3801 | 0 | 1.47E-03 | 1.471 | 0.03151 | 1.272 |
| 1 | rs1252101* | 235170243 | T | C | 0.0005964 | 0.001748 | 0.8164 | 0.816 | 0.2717 | 17.22 | 1.51E-03 | 0.763 | 0.08605 | 0.8689 |
| 3 | rs9846736* | 31586782 | A | G | 0.0667 | 0.4384 | 0.8848 | 0.8781 | 0.0121 | 84.14 | 2.00E-03 | 0.7414 | 0.6922 | 1.037 |
| 18 | rs1941065* | 10012266 | T | A | 0.3933 | 0.8165 | 1.0667 | 1.0633 | 0.0005 | 91.82 | 2.11E-03 | 1.385 | 0.05854 | 0.8159 |
| 4 | rs3857116 | 24421504 | T | C | 0.0145 | 0.1797 | 0.8028 | 0.7966 | 0.0598 | 71.78 | 2.20E-03 | 0.6704 | 0.6207 | 0.9409 |
| 7 | rs2215595* | 28424667 | A | G | 0.07051 | 0.414 | 0.8926 | 0.8832 | 0.0159 | 82.82 | 2.66E-03 | 0.757 | 0.7602 | 1.026 |
| 7 | rs1735090* | 152626909 | A | G | 0.002534 | 0.01307 | 1.2701 | 1.2707 | 0.2228 | 32.7 | 2.75E-03 | 1.401 | 0.1938 | 1.155 |
| 5 | rs5023585* | 109381500 | A | G | 0.03098 | 0.2795 | 1.1841 | 1.1908 | 0.0394 | 76.43 | 2.80E-03 | 1.402 | 0.8903 | 1.015 |
| 17 | rs2173202* | 36488761 | T | C | 0.08774 | 0.4731 | 1.1031 | 1.1082 | 0.0128 | 83.87 | 2.89E-03 | 1.28 | 0.6196 | 0.9612 |
| 7 | rs3807660* | 77656088 | C | T | 0.0005955 | 0.0005955 | 1.215 | 1.215 | 0.422 | 0 | 3.05E-03 | 1.274 | 0.05485 | 1.163 |
| 11 | rs11218350* | 120957861 | T | A | 0.0001791 | 0.0001791 | 1.283 | 1.283 | 0.6155 | 0 | 3.22E-03 | 1.329 | 0.01767 | 1.243 |
| 16 | rs4426363* | 78595960 | A | G | 0.02527 | 0.2356 | 1.1597 | 1.1629 | 0.0548 | 72.88 | 3.31E-03 | 1.322 | 0.7861 | 1.025 |
| 12 | rs17375557* | 95442660 | A | G | 0.0001755 | 0.0001755 | 1.3348 | 1.3348 | 0.6083 | 0 | 3.34E-03 | 1.393 | 0.016 | 1.287 |
| 12 | rs3858655* | 95404373 | T | G | 0.0001478 | 0.0001478 | 1.3425 | 1.3425 | 0.637 | 0 | 3.46E-03 | 1.397 | 0.01329 | 1.298 |
| 9 | rs1323341* | 14443010 | A | G | 0.003445 | 0.01636 | 1.224 | 1.2252 | 0.221 | 33.23 | 3.54E-03 | 1.336 | 0.2095 | 1.128 |
| 13 | rs4408437* | 57133947 | C | T | 0.46 | 0.7993 | 1.0564 | 1.0648 | 0.0009 | 90.94 | 3.57E-03 | 1.364 | 0.07548 | 0.8325 |
| 16 | rs1075905* | 9015599 | C | T | 0.4162 | 0.8162 | 1.0663 | 1.0613 | 0.0012 | 90.46 | 4.28E-03 | 1.37 | 0.08089 | 0.8213 |
| 3 | rs9862718* | 54399741 | A | G | 0.2329 | 0.6462 | 1.0828 | 1.0897 | 0.0051 | 87.26 | 4.32E-03 | 1.315 | 0.2787 | 0.9046 |
| 11 | rs2957761* | 24098510 | T | A | 0.02291 | 0.1774 | 0.8518 | 0.8447 | 0.0772 | 67.97 | 4.48E-03 | 0.7424 | 0.6175 | 0.9536 |
| 3 | rs3774108* | 10898193 | C | A | 0.1386 | 0.5644 | 1.1068 | 1.1051 | 0.0114 | 84.39 | 4.58E-03 | 1.314 | 0.4487 | 0.9289 |
| 9 | rs11795150* | 16095961 | C | A | 0.1407 | 0.5276 | 0.9177 | 0.9119 | 0.0123 | 84.04 | 4.63E-03 | 0.787 | 0.5143 | 1.054 |
| 10 | rs7087228* | 8304160 | T | C | 0.1137 | 0.4709 | 0.9068 | 0.8996 | 0.0179 | 82.16 | 4.91E-03 | 0.7755 | 0.6421 | 1.04 |
| 2 | rs17334527* | 77117777 | C | A | 0.07057 | 0.4161 | 1.1485 | 1.1435 | 0.0313 | 78.44 | 5.02E-03 | 1.347 | 0.7711 | 0.9685 |
| 2 | rs4973006* | 228455120 | C | A | 0.316 | 0.6942 | 0.9443 | 0.9367 | 0.0036 | 88.17 | 5.05E-03 | 0.7923 | 0.2054 | 1.105 |
| 2 | rs16831315* | 135541544 | G | A | 0.005957 | 0.02371 | 1.2469 | 1.2478 | 0.2226 | 32.76 | 5.09E-03 | 1.378 | 0.2671 | 1.133 |
| 2 | rs11892271* | 52237202 | A | G | 0.1269 | 0.4919 | 1.1723 | 1.1851 | 0.0178 | 82.19 | 5.33E-03 | 1.521 | 0.6016 | 0.9278 |
| 3 | rs17233898* | 5807331 | T | C | 0.2222 | 0.5785 | 0.9093 | 0.8934 | 0.0096 | 85.09 | 6.00E-03 | 0.7271 | 0.4085 | 1.091 |
| 13 | rs4941455* | 42799471 | A | G | 0.001042 | 0.001042 | 0.7776 | 0.7776 | 0.5799 | 0 | 7.34E-03 | 0.7441 | 0.04799 | 0.8101 |
| 10 | rs7898110* | 53562357 | C | A | 0.1026 | 0.4195 | 1.1068 | 1.1134 | 0.0325 | 78.12 | 7.35E-03 | 1.274 | 0.7787 | 0.9763 |
| 15 | rs17191351* | 58418368 | T | C | 0.07427 | 0.4003 | 1.1614 | 1.1522 | 0.0449 | 75.13 | 7.43E-03 | 1.36 | 0.8102 | 0.971 |
| 2 | rs11888564* | 173274379 | A | G | 0.5733 | 0.7784 | 0.9578 | 0.9359 | 0.0022 | 89.3 | 7.81E-03 | 0.7376 | 0.1043 | 1.181 |
| 6 | rs9368394* | 22132981 | T | C | 0.1877 | 0.494 | 0.8499 | 0.8231 | 0.0224 | 80.83 | 9.07E-03 | 0.6144 | 0.6137 | 1.086 |
| 8 | rs12546220* | 70536282 | A | G | 0.02624 | 0.1138 | 1.1589 | 1.1616 | 0.1536 | 50.89 | 1.02E-02 | 1.28 | 0.5292 | 1.059 |
| 15 | rs1870301* | 93412015 | T | C | 0.002737 | 0.002737 | 1.2672 | 1.2672 | 0.4876 | 0 | 1.06E-02 | 1.344 | 0.08487 | 1.204 |
| 6 | rs3892789* | 111403162 | G | A | 0.08821 | 0.3686 | 1.1362 | 1.1364 | 0.0577 | 72.23 | 1.08E-02 | 1.31 | 0.8927 | 0.9858 |
| 13 | rs9542253* | 69752150 | T | A | 0.8592 | 0.9406 | 1.0104 | 1.0147 | 0.0008 | 91.06 | 1.17E-02 | 1.234 | 0.027 | 0.835 |
| 8 | rs1845040* | 19612672 | G | A | 0.216 | 0.5548 | 0.9311 | 0.9266 | 0.0254 | 79.99 | 1.34E-02 | 0.8134 | 0.5174 | 1.053 |
| 6 | rs7774757* | 155417681 | C | A | 0.024 | 0.0705 | 1.1383 | 1.1395 | 0.2086 | 36.75 | 1.35E-02 | 1.227 | 0.4479 | 1.062 |
| 16 | rs2306743* | 34649292 | C | G | 0.5776 | 0.7698 | 0.951 | 0.9283 | 0.0051 | 87.27 | 1.36E-02 | 0.7172 | 0.1443 | 1.193 |
| 5 | rs4146606* | 34539764 | G | A | 0.1657 | 0.4811 | 0.9242 | 0.9211 | 0.0404 | 76.21 | 1.47E-02 | 0.8188 | 0.6758 | 1.034 |
| 11 | rs10501659* | 87513536 | T | C | 0.03482 | 0.09683 | 1.1376 | 1.1389 | 0.1998 | 39.16 | 1.68E-02 | 1.234 | 0.5295 | 1.055 |
| 3 | rs9289008* | 116302696 | G | A | 0.008902 | 0.008902 | 1.1768 | 1.1768 | 0.4369 | 0 | 1.68E-02 | 1.236 | 0.1885 | 1.122 |
| 13 | rs914480* | 73830626 | A | G | 0.003029 | 0.003029 | 1.1864 | 1.1864 | 0.6723 | 0 | 1.86E-02 | 1.217 | 0.06444 | 1.159 |
| 6 | rs9322458* | 154635053 | A | T | 0.04211 | 0.1146 | 1.1723 | 1.1742 | 0.1934 | 40.89 | 1.86E-02 | 1.303 | 0.5729 | 1.063 |
| 1 | rs880385* | 55592687 | G | T | 0.1494 | 0.4072 | 1.0851 | 1.088 | 0.0728 | 68.94 | 2.17E-02 | 1.206 | 0.8367 | 0.9839 |
| 7 | rs2707469* | 120764122 | G | A | 0.3017 | 0.6507 | 1.0826 | 1.0802 | 0.0267 | 79.63 | 2.17E-02 | 1.28 | 0.3919 | 0.9105 |
| 1 | rs4650704* | 173385546 | G | A | 0.07099 | 0.2059 | 1.1407 | 1.1418 | 0.1507 | 51.58 | 2.17E-02 | 1.269 | 0.7814 | 1.029 |
| 12 | rs782103* | 57122898 | T | C | 0.1474 | 0.3941 | 1.0939 | 1.0951 | 0.0854 | 66.21 | 2.46E-02 | 1.219 | 0.8608 | 0.9849 |
| 4 | rs17293620* | 184539543 | T | G | 0.04602 | 0.09397 | 0.8473 | 0.8454 | 0.228 | 31.18 | 2.46E-02 | 0.761 | 0.5237 | 0.9302 |
| 16 | rs12933414* | 9401468 | G | A | 0.02138 | 0.02138 | 0.877 | 0.877 | 0.3734 | 0 | 2.54E-02 | 0.8316 | 0.2947 | 0.9206 |
| 18 | rs9950038* | 24262559 | C | A | 0.07223 | 0.1734 | 1.1078 | 1.1095 | 0.1802 | 44.31 | 2.70E-02 | 1.2 | 0.7031 | 1.03 |
| 18 | rs1372622* | 2619368 | G | A | 0.04536 | 0.07543 | 1.15 | 1.1505 | 0.2588 | 21.57 | 2.72E-02 | 1.247 | 0.516 | 1.065 |
| 5 | rs11745313* | 147552456 | G | A | 0.6311 | 0.8189 | 0.9727 | 0.9663 | 0.0097 | 85.06 | 2.73E-02 | 0.8311 | 0.153 | 1.121 |
| 5 | rs10064352* | 75512971 | A | G | 0.03271 | 0.03437 | 1.131 | 1.131 | 0.3127 | 1.91 | 2.73E-02 | 1.201 | 0.4064 | 1.069 |
| 3 | rs1348213* | 77398339 | A | G | 0.3603 | 0.6471 | 0.9479 | 0.9435 | 0.0299 | 78.8 | 2.75E-02 | 0.8298 | 0.4018 | 1.07 |
| 9 | rs10818226* | 120523994 | G | C | 0.2127 | 0.4965 | 1.0884 | 1.0888 | 0.0658 | 70.46 | 2.90E-02 | 1.234 | 0.6774 | 0.9609 |
| 6 | rs2743937* | 29915902 | C | T | 0.01814 | 0.01814 | 0.8585 | 0.8585 | 0.4468 | 0 | 2.95E-02 | 0.8149 | 0.2315 | 0.8992 |
| 15 | rs1989658* | 23376708 | G | T | 0.2581 | 0.537 | 1.0694 | 1.0729 | 0.0547 | 72.91 | 2.97E-02 | 1.204 | 0.6066 | 0.9585 |
| 17 | rs10852877* | 6230343 | T | C | 0.2267 | 0.4951 | 1.097 | 1.0999 | 0.0686 | 69.85 | 3.12E-02 | 1.266 | 0.6859 | 0.9577 |
| 8 | rs4373539* | 5063717 | C | T | 0.08485 | 0.1852 | 1.1209 | 1.1243 | 0.1829 | 43.62 | 3.15E-02 | 1.233 | 0.7227 | 1.033 |
| 12 | rs6581677* | 65056646 | G | C | 0.1375 | 0.3196 | 1.0892 | 1.0912 | 0.1276 | 56.93 | 3.34E-02 | 1.193 | 0.9896 | 1.001 |
| 12 | rs931882* | 59868562 | G | A | 0.01561 | 0.01561 | 1.1541 | 1.1541 | 0.5496 | 0 | 3.42E-02 | 1.197 | 0.1881 | 1.115 |
| 9 | rs1329393* | 97358747 | T | C | 0.4649 | 0.6857 | 0.9455 | 0.9347 | 0.0302 | 78.7 | 3.62E-02 | 0.7887 | 0.3534 | 1.101 |
| 8 | rs10104759* | 35423940 | G | T | 0.04838 | 0.04918 | 0.8894 | 0.8894 | 0.3155 | 0.75 | 3.69E-02 | 0.8351 | 0.4568 | 0.941 |
| 13 | rs9568005* | 47594325 | T | C | 0.00717 | 0.00717 | 1.2021 | 1.2021 | 0.7571 | 0 | 3.72E-02 | 1.229 | 0.08353 | 1.178 |
| 3 | rs12695382* | 120430861 | G | A | 0.402 | 0.6976 | 1.0764 | 1.0747 | 0.0349 | 77.53 | 3.72E-02 | 1.293 | 0.362 | 0.8924 |
| 12 | rs11116324* | 83254244 | T | C | 0.5339 | 0.7382 | 0.962 | 0.954 | 0.0242 | 80.31 | 3.79E-02 | 0.8271 | 0.2788 | 1.096 |
| 12 | rs4931122* | 28992287 | A | G | 0.03158 | 0.03158 | 0.884 | 0.884 | 0.4228 | 0 | 3.89E-02 | 0.8425 | 0.3165 | 0.9237 |
| 14 | rs10141804* | 70318983 | T | C | 0.008804 | 0.008804 | 1.1756 | 1.1756 | 0.7464 | 0 | 3.93E-02 | 1.2 | 0.09745 | 1.153 |
| 3 | rs4419372* | 61580831 | C | T | 0.2736 | 0.5005 | 0.926 | 0.92 | 0.0789 | 67.61 | 4.18E-02 | 0.8104 | 0.6937 | 1.038 |
| 20 | rs214760* | 2223976 | C | T | 0.1249 | 0.2374 | 1.1183 | 1.1188 | 0.1924 | 41.16 | 4.42E-02 | 1.231 | 0.8613 | 1.018 |
| 4 | rs1961460* | 82393522 | T | C | 0.6388 | 0.8063 | 0.9714 | 0.9654 | 0.0205 | 81.38 | 4.50E-02 | 0.835 | 0.2106 | 1.113 |
| 3 | rs7641152* | 194502461 | T | C | 0.07434 | 0.09365 | 0.9003 | 0.9 | 0.2855 | 12.34 | 4.50E-02 | 0.8428 | 0.5774 | 0.9558 |
| 9 | rs10491590* | 728940 | T | C | 0.4034 | 0.6649 | 1.0972 | 1.0991 | 0.0494 | 74.1 | 4.62E-02 | 1.368 | 0.4303 | 0.8842 |
| 9 | rs11999298* | 36884151 | T | C | 0.005207 | 0.005207 | 1.3828 | 1.3828 | 0.9208 | 0 | 4.71E-02 | 1.4 | 0.04759 | 1.368 |
| 21 | rs2824723* | 18595079 | C | T | 0.3496 | 0.5732 | 0.9404 | 0.9368 | 0.0784 | 67.73 | 5.48E-02 | 0.8329 | 0.5939 | 1.05 |
| 14 | rs7140637* | 49892268 | C | G | 0.2519 | 0.4521 | 1.0716 | 1.0728 | 0.1212 | 58.37 | 5.64E-02 | 1.179 | 0.7914 | 0.9779 |
| 14 | rs7161259* | 91214828 | T | G | 0.04102 | 0.04102 | 0.8893 | 0.8893 | 0.5023 | 0 | 5.75E-02 | 0.8543 | 0.3131 | 0.9228 |
| 9 | rs10123421* | 76903099 | C | T | 0.01441 | 0.01441 | 0.8457 | 0.8457 | 0.7977 | 0 | 6.00E-02 | 0.8304 | 0.1121 | 0.8601 |
| 2 | rs542972* | 239032770 | T | C | 0.9538 | 0.949 | 0.9965 | 0.9902 | 0.0111 | 84.5 | 6.01E-02 | 0.8485 | 0.08754 | 1.153 |
| 3 | rs10804515* | 116184747 | T | C | 0.01047 | 0.01047 | 1.1783 | 1.1783 | 0.9209 | 0 | 6.26E-02 | 1.186 | 0.07803 | 1.171 |
| 1 | rs4659986* | 238866166 | C | T | 0.09538 | 0.09538 | 1.1091 | 1.1091 | 0.3499 | 0 | 6.66E-02 | 1.177 | 0.5851 | 1.048 |
| 9 | rs7045215* | 104739847 | G | C | 0.2255 | 0.3717 | 1.078 | 1.079 | 0.1697 | 46.98 | 6.77E-02 | 1.176 | 0.9265 | 0.992 |
| 5 | rs4380636* | 93203859 | A | G | 0.633 | 0.7137 | 0.9448 | 0.9167 | 0.0494 | 74.11 | 6.89E-02 | 0.7155 | 0.3669 | 1.15 |
| 5 | rs1428558* | 73887534 | T | C | 0.06643 | 0.06643 | 1.1114 | 1.1114 | 0.459 | 0 | 7.12E-02 | 1.162 | 0.4149 | 1.067 |
| 9 | rs935466* | 87308057 | T | C | 0.025 | 0.025 | 1.1376 | 1.1376 | 0.7258 | 0 | 7.25E-02 | 1.162 | 0.1679 | 1.116 |
| 1 | rs1874406* | 64547498 | T | C | 0.6556 | 0.8111 | 1.0307 | 1.0334 | 0.0423 | 75.76 | 7.68E-02 | 1.187 | 0.272 | 0.9013 |
| 2 | rs565394* | 21259094 | G | A | 0.07312 | 0.07312 | 0.9032 | 0.9032 | 0.4756 | 0 | 7.89E-02 | 0.8659 | 0.4256 | 0.9391 |
| 7 | rs851681* | 147107591 | A | C | 0.8791 | 0.9743 | 0.9908 | 0.995 | 0.0107 | 84.64 | 8.33E-02 | 1.163 | 0.05939 | 0.8526 |
| 11 | rs10488779* | 79494759 | C | T | 0.2498 | 0.3645 | 0.9263 | 0.9251 | 0.1967 | 40.01 | 8.37E-02 | 0.8472 | 0.9481 | 1.006 |
| 5 | rs10038955* | 155681482 | G | T | 0.2553 | 0.3525 | 1.0714 | 1.0726 | 0.214 | 35.24 | 9.11E-02 | 1.159 | 0.9684 | 0.9967 |
| 16 | rs1836872* | 62621225 | A | G | 0.2587 | 0.361 | 1.112 | 1.1127 | 0.2137 | 35.32 | 9.29E-02 | 1.252 | 0.9453 | 0.991 |
| 10 | rs10794730* | 1237022 | T | C | 0.04638 | 0.04638 | 1.1335 | 1.1335 | 0.6848 | 0 | 9.39E-02 | 1.164 | 0.2503 | 1.106 |
| 8 | rs4737291* | 70924348 | C | T | 0.2222 | 0.2808 | 1.0819 | 1.0826 | 0.2538 | 23.2 | 9.57E-02 | 1.168 | 0.9325 | 1.008 |
| 5 | rs2120913* | 100096374 | A | G | 0.01919 | 0.01919 | 0.874 | 0.874 | 0.9501 | 0 | 9.60E-02 | 0.8707 | 0.09911 | 0.877 |
| 2 | rs10932541* | 215099110 | T | C | 0.3152 | 0.8244 | 1.0691 | 1.0475 | 0.0018 | 89.75 | 9.76E-02 | 0.8479 | 0.004584 | 1.288 |
| 2 | rs4340489* | 14583293 | A | G | 0.9191 | 0.9398 | 1.0058 | 1.0095 | 0.0277 | 79.36 | 9.78E-02 | 1.145 | 0.1459 | 0.8917 |
| 10 | rs6602686* | 13904269 | A | G | 0.1604 | 0.1604 | 1.0856 | 1.0856 | 0.3505 | 0 | 9.86E-02 | 1.149 | 0.7142 | 1.03 |
| 3 | rs13314127* | 189952658 | T | C | 0.1164 | 0.1164 | 0.9065 | 0.9065 | 0.4652 | 0 | 1.07E-01 | 0.8644 | 0.5306 | 0.9471 |
| 5 | rs6893150* | 80545692 | A | G | 0.3509 | 0.4808 | 1.0673 | 1.0676 | 0.1837 | 43.42 | 1.08E-01 | 1.172 | 0.7839 | 0.9734 |
| 15 | rs8038473* | 96097480 | G | A | 0.09027 | 0.09027 | 1.1052 | 1.1052 | 0.5924 | 0 | 1.18E-01 | 1.142 | 0.3959 | 1.072 |
| 11 | rs1474844* | 32266552 | G | A | 0.1892 | 0.1892 | 0.9234 | 0.9234 | 0.3766 | 0 | 1.21E-01 | 0.8737 | 0.7435 | 0.9727 |
| 6 | rs6934412* | 85119973 | T | C | 0.0619 | 0.0619 | 0.8902 | 0.8902 | 0.7271 | 0 | 1.23E-01 | 0.8702 | 0.2674 | 0.9089 |
| 16 | rs2055846* | 61733578 | T | C | 0.3804 | 0.4847 | 0.9478 | 0.9467 | 0.1995 | 39.24 | 1.25E-01 | 0.8737 | 0.795 | 1.022 |
| 8 | rs10954969* | 34724264 | G | C | 0.002891 | 0.002891 | 1.2539 | 1.2539 | 0.4883 | 0 | 1.38E-01 | 1.183 | 0.007292 | 1.315 |
| 21 | rs2833822* | 32726508 | C | T | 0.5691 | 0.6894 | 1.0333 | 1.0351 | 0.1341 | 55.45 | 1.39E-01 | 1.13 | 0.5281 | 0.9508 |
| 4 | rs11727321* | 43344913 | T | G | 0.4439 | 0.5337 | 0.9096 | 0.9057 | 0.1989 | 39.41 | 1.43E-01 | 0.7671 | 0.7522 | 1.055 |
| 6 | rs6908632* | 17505095 | C | T | 0.6988 | 0.7959 | 1.0241 | 1.0268 | 0.0974 | 63.61 | 1.44E-01 | 1.139 | 0.3845 | 0.9284 |
| 13 | rs1023102* | 72723017 | C | T | 0.3373 | 0.3721 | 1.0561 | 1.0565 | 0.2793 | 14.56 | 1.48E-01 | 1.126 | 0.9543 | 0.9955 |
| 2 | rs10514631* | 19767453 | C | T | 0.1045 | 0.1045 | 1.1298 | 1.1298 | 0.6476 | 0 | 1.48E-01 | 1.172 | 0.3826 | 1.094 |
| 12 | rs10506984* | 89217396 | G | C | 0.02549 | 0.02549 | 0.8656 | 0.8656 | 0.8737 | 0 | 1.49E-01 | 0.8748 | 0.08662 | 0.857 |
| 6 | rs2085672* | 153903143 | G | C | 0.5133 | 0.6211 | 1.0405 | 1.0417 | 0.1734 | 46.04 | 1.52E-01 | 1.133 | 0.6328 | 0.9604 |
| 2 | rs17499330* | 118231401 | T | C | 0.4257 | 0.5047 | 1.0656 | 1.0667 | 0.2246 | 32.2 | 1.53E-01 | 1.178 | 0.7868 | 0.9705 |
| 6 | rs2800708* | 127479310 | G | A | 0.5662 | 0.6713 | 0.9679 | 0.9661 | 0.1534 | 50.94 | 1.53E-01 | 0.8892 | 0.5634 | 1.046 |
| 13 | rs9564316* | 33884770 | A | T | 0.2386 | 0.2386 | 0.9184 | 0.9184 | 0.3988 | 0 | 1.54E-01 | 0.8614 | 0.7852 | 0.9732 |
| 1 | rs10918985* | 167022584 | A | C | 0.9547 | 0.9968 | 0.9967 | 1.0005 | 0.0454 | 75.02 | 1.59E-01 | 1.127 | 0.1539 | 0.8904 |
| 2 | rs1227921* | 162400446 | C | G | 0.5686 | 0.6769 | 1.0344 | 1.0352 | 0.1607 | 49.18 | 1.61E-01 | 1.126 | 0.5657 | 0.9535 |
| 6 | rs1499571* | 119871783 | T | C | 0.05908 | 0.05908 | 0.8958 | 0.8958 | 0.9026 | 0 | 1.64E-01 | 0.8891 | 0.2004 | 0.9019 |
| 10 | rs1665571* | 80081635 | G | A | 0.1706 | 0.1706 | 1.0957 | 1.0957 | 0.5437 | 0 | 1.66E-01 | 1.143 | 0.5665 | 1.054 |
| 9 | rs7856164* | 110574103 | G | T | 0.3129 | 0.3129 | 0.909 | 0.909 | 0.3504 | 0 | 1.71E-01 | 0.8242 | 0.9013 | 0.9843 |
| 7 | rs1468244* | 11431856 | T | C | 0.8818 | 0.9076 | 1.0089 | 1.0119 | 0.0863 | 66.01 | 1.79E-01 | 1.122 | 0.2812 | 0.9154 |
| 10 | rs2804869* | 33856365 | A | G | 0.1181 | 0.1181 | 1.0951 | 1.0951 | 0.7299 | 0 | 1.80E-01 | 1.118 | 0.375 | 1.074 |
| 20 | rs730520* | 7635252 | A | G | 0.4188 | 0.4416 | 1.0469 | 1.0471 | 0.2909 | 10.36 | 1.85E-01 | 1.114 | 0.8811 | 0.9883 |
| 7 | rs9639329* | 19366356 | C | T | 0.6407 | 0.712 | 0.9724 | 0.9705 | 0.1769 | 45.16 | 1.93E-01 | 0.8929 | 0.5582 | 1.05 |
| 15 | rs12595616* | 89364517 | C | T | 0.06331 | 0.06331 | 1.1135 | 1.1135 | 0.9938 | 0 | 1.97E-01 | 1.114 | 0.1829 | 1.113 |
| 6 | rs9464886* | 16495782 | G | A | 0.0949 | 0.0949 | 1.125 | 1.125 | 0.8849 | 0 | 2.07E-01 | 1.137 | 0.269 | 1.114 |
| 12 | rs2114862* | 105120036 | G | C | 0.3382 | 0.3382 | 0.9339 | 0.9339 | 0.4065 | 0 | 2.08E-01 | 0.877 | 0.8977 | 0.9875 |
| 4 | rs12509776* | 116608823 | T | C | 0.1464 | 0.1464 | 0.9061 | 0.9061 | 0.7452 | 0 | 2.16E-01 | 0.8853 | 0.4076 | 0.9253 |
| 5 | rs279108* | 100110254 | C | T | 0.07823 | 0.07823 | 0.9039 | 0.9039 | 0.9962 | 0 | 2.21E-01 | 0.9036 | 0.2051 | 0.9041 |
| 7 | rs1100508* | 124132594 | G | C | 0.1198 | 0.1198 | 0.9154 | 0.9154 | 0.8755 | 0 | 2.34E-01 | 0.907 | 0.3114 | 0.9233 |
| 1 | rs10800533* | 160683253 | A | G | 0.4608 | 0.4608 | 0.9446 | 0.9446 | 0.3601 | 0 | 2.41E-01 | 0.8766 | 0.9231 | 1.01 |
| 7 | rs6967565* | 8096253 | A | G | 0.8669 | 0.8955 | 1.0109 | 1.0125 | 0.1432 | 53.34 | 2.42E-01 | 1.115 | 0.3687 | 0.9221 |
| 7 | rs6463813* | 8476768 | A | G | 0.1071 | 0.1071 | 1.0963 | 1.0963 | 0.9427 | 0 | 2.45E-01 | 1.101 | 0.2638 | 1.092 |
| 4 | rs17553672 | 24448662 | G | T | 0.1984 | 0.1984 | 1.0877 | 1.0877 | 0.7151 | 0 | 2.48E-01 | 1.115 | 0.5038 | 1.063 |
| 2 | rs6738950* | 227755364 | T | C | 0.5392 | 0.5392 | 0.9505 | 0.9505 | 0.3204 | 0 | 2.54E-01 | 0.8729 | 0.8035 | 1.029 |
| 1 | rs4951701* | 209232629 | C | G | 0.09034 | 0.09034 | 0.9048 | 0.9048 | 0.9433 | 0 | 2.61E-01 | 0.9088 | 0.2043 | 0.9012 |
| 5 | rs17651115* | 75475886 | C | G | 0.5076 | 0.5076 | 0.9627 | 0.9627 | 0.3622 | 0 | 2.64E-01 | 0.9115 | 0.8792 | 1.012 |
| 14 | rs11624725* | 89783362 | T | G | 0.1676 | 0.1676 | 0.9229 | 0.9229 | 0.8361 | 0 | 2.66E-01 | 0.9116 | 0.3984 | 0.9338 |
| 1 | rs1728232* | 113366167 | A | G | 0.414 | 0.414 | 0.9539 | 0.9539 | 0.4603 | 0 | 2.72E-01 | 0.9125 | 0.9395 | 0.9939 |
| 2 | rs17644183* | 168343798 | C | T | 0.1229 | 0.1229 | 1.0935 | 1.0935 | 0.9937 | 0 | 2.80E-01 | 1.094 | 0.2676 | 1.093 |
| 6 | rs7762948* | 34094069 | A | G | 0.4277 | 0.4277 | 1.0955 | 1.0955 | 0.4701 | 0 | 2.82E-01 | 1.189 | 0.9683 | 1.007 |
| 8 | rs752036* | 101695417 | A | G | 0.9532 | 0.939 | 0.9966 | 0.9937 | 0.1601 | 49.33 | 2.85E-01 | 0.9123 | 0.3633 | 1.076 |
| 10 | rs2784773* | 81909780 | A | G | 0.415 | 0.415 | 0.9535 | 0.9535 | 0.4873 | 0 | 2.87E-01 | 0.9138 | 0.9128 | 0.9912 |
| 5 | rs10516147* | 178027090 | G | A | 0.697 | 0.703 | 0.9731 | 0.9729 | 0.3036 | 5.52 | 3.11E-01 | 0.9018 | 0.6724 | 1.042 |
| 14 | rs10498420* | 49483793 | C | T | 0.5006 | 0.5006 | 1.0389 | 1.0389 | 0.4515 | 0 | 3.12E-01 | 1.086 | 0.9704 | 0.9971 |
| 13 | rs912044* | 38962446 | G | A | 0.487 | 0.487 | 1.0408 | 1.0408 | 0.5154 | 0 | 3.43E-01 | 1.082 | 0.9596 | 1.004 |
| 3 | rs2292518* | 112928236 | C | T | 0.4182 | 0.4182 | 1.0491 | 1.0491 | 0.602 | 0 | 3.48E-01 | 1.084 | 0.8136 | 1.019 |
| 9 | rs10817664* | 116472734 | A | C | 0.4902 | 0.4902 | 1.045 | 1.045 | 0.5391 | 0 | 3.56E-01 | 1.088 | 0.945 | 1.006 |
| 8 | rs10957988* | 81983149 | T | C | 0.1467 | 0.1467 | 0.918 | 0.918 | 0.8892 | 0 | 3.67E-01 | 0.9259 | 0.2522 | 0.9108 |
| 21 | rs189900* | 23322128 | G | A | 0.9554 | 0.9548 | 1.0032 | 1.0038 | 0.2389 | 27.89 | 3.75E-01 | 1.075 | 0.436 | 0.9403 |
| 3 | rs11718847* | 43026662 | A | T | 0.6437 | 0.6437 | 1.0411 | 1.0411 | 0.4411 | 0 | 3.83E-01 | 1.115 | 0.8356 | 0.9751 |
| 7 | rs7808652* | 88881333 | C | A | 0.6883 | 0.6883 | 0.9731 | 0.9731 | 0.4207 | 0 | 3.89E-01 | 0.9184 | 0.7915 | 1.025 |
| 10 | rs2474568* | 38423153 | T | A | 0.625 | 0.625 | 1.0286 | 1.0286 | 0.4879 | 0 | 4.01E-01 | 1.072 | 0.8962 | 0.9896 |
| 11 | rs11020505* | 93117781 | A | C | 0.2502 | 0.2502 | 0.9354 | 0.9354 | 0.9516 | 0 | 4.02E-01 | 0.9319 | 0.4296 | 0.9385 |
| 6 | rs4707795 | 94111589 | T | C | 0.09213 | 0.09213 | 1.1386 | 1.1386 | 0.6444 | 0 | 4.05E-01 | 1.097 | 0.1235 | 1.178 |
| 8 | rs4292660* | 99865472 | G | A | 0.4121 | 0.4121 | 0.9528 | 0.9528 | 0.7196 | 0 | 4.08E-01 | 0.9322 | 0.7335 | 0.9725 |
| 2 | rs3770542* | 216555396 | A | T | 0.265 | 0.265 | 0.9142 | 0.9142 | 0.9378 | 0 | 4.09E-01 | 0.9082 | 0.4511 | 0.9197 |
| 2 | rs4663128* | 234881472 | C | A | 0.3889 | 0.3889 | 0.9501 | 0.9501 | 0.783 | 0 | 4.26E-01 | 0.9341 | 0.668 | 0.9652 |
| 14 | rs1189041* | 55931553 | C | A | 0.9215 | 0.9287 | 0.9892 | 0.9879 | 0.2185 | 33.95 | 4.30E-01 | 1.129 | 0.3413 | 0.8602 |
| 13 | rs1885952* | 18548695 | C | T | 0.5187 | 0.7217 | 0.9569 | 0.9588 | 0.0838 | 66.55 | 4.34E-01 | 1.08 | 0.0949 | 0.853 |
| 10 | rs10764825* | 130559927 | G | A | 0.2928 | 0.6295 | 0.9399 | 0.9419 | 0.0353 | 77.43 | 4.41E-01 | 1.067 | 0.02611 | 0.8325 |
| 10 | rs9299582* | 51974286 | G | A | 0.5789 | 0.5789 | 1.0326 | 1.0326 | 0.6037 | 0 | 4.46E-01 | 1.065 | 0.9673 | 1.003 |
| 3 | rs6445058* | 173498694 | A | C | 0.7826 | 0.7826 | 0.9845 | 0.9845 | 0.4482 | 0 | 4.60E-01 | 0.9414 | 0.7458 | 1.026 |
| 8 | rs16938580* | 74249204 | A | G | 0.7996 | 0.7996 | 1.0202 | 1.0202 | 0.4493 | 0 | 4.71E-01 | 1.084 | 0.7268 | 0.9622 |
| 13 | rs9554679* | 99787063 | A | G | 0.7376 | 0.7376 | 0.981 | 0.981 | 0.5107 | 0 | 4.80E-01 | 0.9431 | 0.8275 | 1.017 |
| 20 | rs1201987* | 58736287 | A | G | 0.5091 | 0.5091 | 0.9597 | 0.9597 | 0.7395 | 0 | 4.85E-01 | 0.9392 | 0.806 | 0.979 |
| 10 | rs12354899* | 86977616 | C | G | 0.0655 | 0.0655 | 1.1179 | 1.1179 | 0.4201 | 0 | 4.91E-01 | 1.062 | 0.05825 | 1.171 |
| 2 | rs10173668* | 192344408 | T | G | 0.6737 | 0.6737 | 0.9755 | 0.9755 | 0.5904 | 0 | 4.96E-01 | 0.9432 | 0.9464 | 1.005 |
| 7 | rs17133918* | 50679766 | T | C | 0.5965 | 0.5965 | 1.0324 | 1.0324 | 0.6765 | 0 | 5.06E-01 | 1.06 | 0.9266 | 1.008 |
| 10 | rs10998835* | 70922049 | A | G | 0.7247 | 0.7247 | 1.0206 | 1.0206 | 0.5793 | 0 | 5.18E-01 | 1.055 | 0.8956 | 0.9895 |
| 2 | rs2139404* | 100608026 | A | G | 0.3286 | 0.6088 | 1.0571 | 1.0538 | 0.0715 | 69.22 | 5.31E-01 | 0.9499 | 0.05115 | 1.166 |
| 9 | rs10758941* | 8126797 | C | T | 0.3031 | 0.3031 | 0.941 | 0.941 | 0.8405 | 0 | 5.67E-01 | 0.9526 | 0.3788 | 0.9302 |
| 18 | rs12961718* | 65238984 | G | A | 0.3278 | 0.3278 | 1.0605 | 1.0605 | 0.8629 | 0 | 5.86E-01 | 1.049 | 0.4094 | 1.071 |
| 4 | rs7656584* | 137628031 | A | G | 0.3421 | 0.3421 | 0.936 | 0.936 | 0.8233 | 0 | 6.11E-01 | 0.9509 | 0.4047 | 0.9218 |
| 5 | rs10040249* | 110625639 | T | C | 0.4901 | 0.4901 | 1.0508 | 1.0508 | 0.9684 | 0 | 6.17E-01 | 1.054 | 0.6346 | 1.048 |
| 9 | rs10759949* | 119632012 | C | A | 0.8302 | 0.8302 | 0.9879 | 0.9879 | 0.6328 | 0 | 6.22E-01 | 0.9605 | 0.8616 | 1.014 |
| 4 | rs17661170* | 171411895 | G | A | 0.4257 | 0.5867 | 0.9468 | 0.9461 | 0.1379 | 54.56 | 6.34E-01 | 1.047 | 0.106 | 0.854 |
| 11 | rs1528641* | 14043034 | A | T | 0.9595 | 0.9595 | 1.0029 | 1.0029 | 0.5423 | 0 | 6.34E-01 | 1.04 | 0.6999 | 0.9699 |
| 4 | rs1383421* | 80154502 | G | T | 0.1014 | 0.1014 | 0.8247 | 0.8247 | 0.3256 | 0 | 6.41E-01 | 0.9254 | 0.06303 | 0.7343 |
| 1 | rs1764844* | 217309442 | C | T | 0.7259 | 0.7259 | 1.0211 | 1.0211 | 0.7798 | 0 | 6.55E-01 | 1.039 | 0.9536 | 1.005 |
| 9 | rs10973109* | 36851407 | G | A | 0.711 | 0.711 | 0.9742 | 0.9742 | 0.8165 | 0 | 6.73E-01 | 0.9573 | 0.9104 | 0.9892 |
| 5 | rs1295212* | 154965952 | T | C | 0.6033 | 0.6308 | 1.0305 | 1.0301 | 0.2853 | 12.41 | 6.79E-01 | 0.966 | 0.2652 | 1.093 |
| 6 | rs1015887* | 144077451 | A | G | 0.5691 | 0.5691 | 1.0331 | 1.0331 | 0.9731 | 0 | 7.14E-01 | 1.031 | 0.6652 | 1.035 |
| 7 | rs193841* | 105374089 | T | C | 0.1721 | 0.1721 | 1.0985 | 1.0985 | 0.35 | 0 | 7.96E-01 | 1.026 | 0.1012 | 1.167 |
| 13 | rs9600236* | 73603200 | T | C | 0.1512 | 0.4188 | 0.8894 | 0.8889 | 0.0744 | 68.59 | 8.08E-01 | 1.028 | 0.02252 | 0.7682 |
| 3 | rs6769328* | 79455526 | C | T | 0.4793 | 0.4793 | 0.9585 | 0.9585 | 0.7166 | 0 | 8.17E-01 | 0.9803 | 0.4467 | 0.9386 |
| 7 | rs10953515* | 106080918 | T | C | 0.3712 | 0.4375 | 1.0557 | 1.0547 | 0.2581 | 21.81 | 8.32E-01 | 0.9814 | 0.1543 | 1.126 |
| 5 | rs7731633* | 85586892 | T | C | 0.2919 | 0.2919 | 0.9414 | 0.9414 | 0.4663 | 0 | 8.35E-01 | 0.9829 | 0.206 | 0.9041 |
| 18 | rs9959583* | 20993778 | T | C | 0.9587 | 0.9587 | 1.003 | 1.003 | 0.8284 | 0 | 8.45E-01 | 1.016 | 0.9104 | 0.9911 |
| 18 | rs4377227* | 20987826 | C | G | 0.7868 | 0.7868 | 1.0155 | 1.0155 | 0.9931 | 0 | 8.48E-01 | 1.016 | 0.8514 | 1.015 |
| 11 | rs1461371* | 26351264 | T | A | 0.6507 | 0.6507 | 1.036 | 1.036 | 0.8678 | 0 | 8.49E-01 | 1.022 | 0.6608 | 1.049 |
| 7 | rs1528502* | 84932177 | G | A | 0.7211 | 0.7211 | 1.0226 | 1.0226 | 0.547 | 0 | 8.50E-01 | 0.9829 | 0.5016 | 1.06 |
| 8 | rs2369548* | 80044352 | C | T | 0.2815 | 0.2815 | 1.0633 | 1.0633 | 0.4378 | 0 | 8.58E-01 | 1.015 | 0.1867 | 1.109 |
| 4 | rs12645979* | 11477195 | G | A | 0.1808 | 0.2044 | 1.0791 | 1.0789 | 0.2932 | 9.5 | 8.62E-01 | 1.014 | 0.09002 | 1.143 |
| 6 | rs1015340* | 144077050 | C | T | 0.6583 | 0.6583 | 1.0254 | 1.0254 | 0.85 | 0 | 8.64E-01 | 1.014 | 0.6558 | 1.036 |
| 6 | rs1220445* | 91712833 | A | G | 0.9814 | 0.9814 | 0.9987 | 0.9987 | 0.8362 | 0 | 8.69E-01 | 0.9866 | 0.8972 | 1.01 |
| 3 | rs17746541* | 60354293 | C | A | 0.3968 | 0.3968 | 1.0506 | 1.0506 | 0.5508 | 0 | 8.78E-01 | 1.013 | 0.304 | 1.086 |
| 12 | rs2731402* | 59992544 | C | T | 0.5989 | 0.5989 | 0.9662 | 0.9662 | 0.4837 | 0 | 8.88E-01 | 1.013 | 0.3867 | 0.9242 |
| 18 | rs7240074* | 25336210 | T | C | 0.6744 | 0.6744 | 0.9763 | 0.9763 | 0.5577 | 0 | 8.97E-01 | 1.011 | 0.4779 | 0.9455 |
| 10 | rs11002539* | 79841011 | C | T | 0.8234 | 0.8234 | 0.9796 | 0.9796 | 0.6953 | 0 | 8.98E-01 | 1.017 | 0.6655 | 0.9461 |
| 12 | rs2178683* | 80465589 | T | C | 0.1793 | 0.2191 | 1.1264 | 1.1254 | 0.2783 | 14.93 | 8.99E-01 | 1.017 | 0.08421 | 1.233 |
| 15 | rs17738626* | 60613489 | T | C | 0.6713 | 0.6713 | 1.0429 | 1.0429 | 0.5962 | 0 | 9.15E-01 | 0.9845 | 0.5036 | 1.094 |
| 1 | rs4653300* | 37796641 | A | G | 0.8244 | 0.8244 | 1.0127 | 1.0127 | 0.9377 | 0 | 9.21E-01 | 1.008 | 0.8256 | 1.017 |
| 6 | rs7759835* | 9529407 | T | C | 0.1126 | 0.28 | 0.8988 | 0.8989 | 0.1423 | 53.54 | 9.34E-01 | 0.9922 | 0.03066 | 0.8145 |
| 15 | rs4146472* | 62087994 | G | A | 0.8423 | 0.8423 | 0.9848 | 0.9848 | 0.7646 | 0 | 9.42E-01 | 1.008 | 0.7246 | 0.9627 |
| 12 | rs6538271* | 90335642 | G | A | 0.3805 | 0.3805 | 1.0864 | 1.0864 | 0.4555 | 0 | 9.45E-01 | 1.009 | 0.2491 | 1.162 |
| 2 | rs6706469* | 240059548 | T | A | 0.426 | 0.426 | 0.9359 | 0.9359 | 0.4011 | 0 | 9.47E-01 | 1.008 | 0.2479 | 0.8763 |
| 6 | rs416101* | 95090722 | C | A | 0.4118 | 0.4118 | 0.9469 | 0.9469 | 0.3693 | 0 | 9.49E-01 | 1.006 | 0.2244 | 0.8928 |
| 3 | rs2979378* | 126779879 | G | T | 0.8033 | 0.8033 | 1.0143 | 1.0143 | 0.7498 | 0 | 9.52E-01 | 0.9951 | 0.6851 | 1.032 |
| 1 | rs2205848* | 167942917 | C | T | 0.2252 | 0.2768 | 1.0799 | 1.0792 | 0.2692 | 18.08 | 9.72E-01 | 1.003 | 0.101 | 1.154 |
| 6 | rs4709680* | 163520791 | C | T | 0.6437 | 0.6437 | 1.0309 | 1.0309 | 0.6797 | 0 | 9.83E-01 | 1.002 | 0.538 | 1.058 |
| 7 | rs193803* | 105394028 | A | G | 0.1578 | 0.314 | 1.1001 | 1.0977 | 0.1713 | 46.57 | 9.83E-01 | 0.998 | 0.04931 | 1.201 |
| 6 | rs2181011* | 70896157 | C | T | 0.4013 | 0.4013 | 1.049 | 1.049 | 0.4215 | 0 | 9.96E-01 | 1 | 0.2445 | 1.096 |

CHR: Chromosome; BP: Base pair position; SNP: Single Nucleotide Polymorphism; A1: Reference allele; A2: Alternative allele; P: Fixed-effects p-value; P(R): Random-effects p-value; OR: Fixed-effects Odds Ratio; OR(R): Random-effects Odds Ratio; Q: p-value for heterogeneity of OR; I: effect size for heterogeneity of OR; The last six columns show the p and OR values obtained in each analyzed sample.

* SNPs selected by two-locus association analyses in the NXC-GWAS sample.

**According to UCSC genome browser (NCBI36/hg18) and dbSNP build 130.
