# Supplementary material for: A Colorectal Cancer Susceptibility New Variant at 4q26 in the Spanish Population Identified by Genome-Wide Association Analysis
Source: PLoS One. 2014 Jun 30;9(6):e101178. doi: 10.1371/journal.pone.0101178 (PMC4076321; doi:10.1371/journal.pone.0101178)
Supplement: Table S4 — SNPs included in the stage II and global meta-analysis results. (DOC) [file pone.0101178.s007.doc]

**Table S4.** SNPs included in the stage II and global meta-analysis results.

|  |  |  |  |  |  |  |  |  |  |  | NXC-GWAS | | NXC-VAL | | Epicolon-GWAS | |
| --- | --- | --- | --- | --- | --- | --- | --- | --- | --- | --- | --- | --- | --- | --- | --- | --- |
| CHR | SNP | BP** | A1 | A2 | P | P(R) | OR | OR(R) | Q | I | p | OR | P | OR | P | OR |
| 15 | rs7171889 | 92377186 | C | A | 0.00003416 | 0.1004 | 1.4247 | 1.414 | 0.0023 | 83.56 | 8.531E-08 | 2.143 | 0.392 | 1.141 | 0.3345 | 1.151 |
| 9 | rs1930551* | 104380162 | T | G | 0.000117 | 0.008854 | 1.4215 | 1.4381 | 0.1005 | 56.48 | 0.00008673 | 1.937 | 0.113 | 1.278 | 0.1561 | 1.237 |
| 9 | rs10990158 | 104335927 | T | A | 0.0001186 | 0.0109 | 1.41 | 1.4245 | 0.0895 | 58.57 | 0.00007396 | 1.908 | 0.0921 | 1.288 | 0.2139 | 1.204 |
| 7 | rs2041001* | 107870335 | G | A | 0.0001538 | 0.002704 | 1.4628 | 1.467 | 0.1989 | 38.08 | 0.0002797 | 1.9 | 0.2026 | 1.243 | 0.07649 | 1.356 |
| 10 | rs941853 | 116189165 | A | G | 0.0001967 | 0.009045 | 0.7928 | 0.7897 | 0.125 | 51.92 | 0.0002035 | 0.6542 | 0.3117 | 0.892 | 0.06749 | 0.8359 |
| 7 | rs4722778 | 28278588 | G | C | 0.000253 | 0.02526 | 0.8273 | 0.8238 | 0.0617 | 64.11 | 0.00009238 | 0.693 | 0.4458 | 0.934 | 0.07482 | 0.8573 |
| 8 | rs7838116 | 40048312 | A | G | 0.0002829 | 0.04108 | 0.7481 | 0.7359 | 0.0302 | 71.42 | 0.00002378 | 0.5351 | 0.2209 | 0.844 | 0.257 | 0.864 |
| 4 | rs1023890* | 118920894 | A | G | 0.0003355 | 0.1969 | 1.1765 | 1.1926 | 0.0001 | 88.92 | 0.0001274 | 1.374 | 0.0001 | 1.355 | 0.2359 | 0.9156 |
| 12 | rs10506984* | 89217396 | G | C | 0.0003479 | 0.0003479 | 0.8334 | 0.8334 | 0.6274 | 0 | 0.1489 | 0.8748 | 0.0866 | 0.857 | 0.003138 | 0.7832 |
| 4 | rs3987* | 118978503 | C | T | 0.0003981 | 0.2205 | 1.1742 | 1.1896 | 0.0001 | 89.72 | 0.0001256 | 1.373 | 8E-05 | 1.363 | 0.1743 | 0.9034 |
| 5 | rs12519633 | 121967053 | T | C | 0.0004171 | 0.04334 | 1.3066 | 1.3223 | 0.0363 | 69.83 | 0.00007004 | 1.737 | 0.5775 | 1.073 | 0.07012 | 1.261 |
| 9 | rs7039568 | 104361604 | T | C | 0.0004172 | 0.03073 | 1.3782 | 1.4017 | 0.0532 | 65.92 | 0.00006898 | 1.959 | 0.1808 | 1.229 | 0.2732 | 1.177 |
| 9 | rs16921774* | 104336206 | T | C | 0.0004792 | 0.02554 | 1.3677 | 1.382 | 0.0743 | 61.54 | 0.0001148 | 1.872 | 0.1824 | 1.228 | 0.285 | 1.172 |
| 4 | rs1459528 | 118969796 | G | A | 0.0004835 | 0.1605 | 1.1709 | 1.1838 | 0.0009 | 85.81 | 0.0001956 | 1.358 | 0.0006 | 1.309 | 0.3916 | 0.9382 |
| 12 | rs1477102 | 95526343 | G | T | 0.0005265 | 0.1021 | 0.8542 | 0.8459 | 0.0064 | 80.19 | 0.00001384 | 0.6978 | 0.0695 | 0.866 | 0.9376 | 0.9942 |
| 4 | rs2169059 | 118926638 | A | C | 0.0005562 | 0.1944 | 1.169 | 1.1829 | 0.0003 | 87.75 | 0.0003896 | 1.338 | 0.0001 | 1.35 | 0.2666 | 0.9206 |
| 9 | rs10990136 | 104298657 | T | C | 0.000831 | 0.03933 | 1.3584 | 1.3762 | 0.0581 | 64.85 | 0.0001182 | 1.902 | 0.1935 | 1.227 | 0.3793 | 1.142 |
| 5 | rs2120913* | 100096374 | A | G | 0.0008529 | 0.0008529 | 0.8586 | 0.8586 | 0.8767 | 0 | 0.09599 | 0.8707 | 0.0991 | 0.877 | 0.01514 | 0.8328 |
| 5 | rs588367 | 16709570 | A | G | 0.0008551 | 0.1177 | 0.853 | 0.8427 | 0.0053 | 80.9 | 0.00002846 | 0.6933 | 0.0422 | 0.845 | 0.8655 | 1.013 |
| 22 | rs6009047 | 45683534 | T | C | 0.000932 | 0.09341 | 0.8541 | 0.8459 | 0.0127 | 77.08 | 0.00001896 | 0.6873 | 0.3167 | 0.921 | 0.4926 | 0.9479 |
| 9 | rs7024470 | 104361506 | G | A | 0.0009419 | 0.03074 | 1.3491 | 1.3686 | 0.0777 | 60.87 | 0.0002208 | 1.863 | 0.1679 | 1.237 | 0.3561 | 1.146 |
| 4 | rs1870481 | 118882468 | T | C | 0.0009771 | 0.2491 | 1.1608 | 1.1763 | 0.0001 | 89.65 | 0.0002399 | 1.351 | 0.0001 | 1.352 | 0.1361 | 0.895 |
| 8 | rs13273088 | 70656528 | G | A | 0.001053 | 0.06495 | 1.201 | 1.2081 | 0.0351 | 70.15 | 0.0001194 | 1.471 | 0.1075 | 1.168 | 0.7186 | 1.034 |
| 4 | rs12503362 | 14452104 | T | C | 0.001066 | 0.03635 | 1.3138 | 1.32 | 0.0799 | 60.42 | 0.0001886 | 1.733 | 0.2874 | 1.165 | 0.3224 | 1.15 |
| 1 | rs550437 | 104121003 | G | C | 0.001197 | 0.08731 | 0.8556 | 0.8516 | 0.0227 | 73.58 | 0.0002376 | 0.722 | 0.9202 | 1.008 | 0.03192 | 0.844 |
| 20 | rs1998087 | 14764382 | A | C | 0.001404 | 0.1171 | 0.824 | 0.8165 | 0.011 | 77.81 | 0.0001501 | 0.6473 | 0.7623 | 1.032 | 0.0268 | 0.8071 |
| 1 | rs2646249 | 104110655 | C | A | 0.001485 | 0.09302 | 0.8585 | 0.8552 | 0.0236 | 73.32 | 0.0002582 | 0.7257 | 0.9047 | 1.01 | 0.03882 | 0.8493 |
| 5 | rs428263 | 16711495 | T | C | 0.001651 | 0.1318 | 0.861 | 0.8521 | 0.0069 | 79.88 | 0.00007656 | 0.7102 | 0.0413 | 0.844 | 0.7614 | 1.024 |
| 5 | rs876095 | 16709803 | T | C | 0.001706 | 0.1282 | 0.8616 | 0.8527 | 0.0079 | 79.33 | 0.00008807 | 0.7124 | 0.0422 | 0.845 | 0.7792 | 1.022 |
| 13 | rs9540846 | 66110941 | A | T | 0.001838 | 0.0532 | 0.7756 | 0.7658 | 0.0581 | 64.86 | 0.0002071 | 0.5726 | 0.2595 | 0.854 | 0.4247 | 0.8992 |
| 11 | rs2403583 | 20334676 | C | A | 0.00185 | 0.1934 | 1.192 | 1.2048 | 0.0016 | 84.45 | 0.00001454 | 1.551 | 0.0593 | 1.202 | 0.5369 | 0.9435 |
| 13 | rs1374482* | 57054311 | T | C | 0.001925 | 0.04653 | 1.1812 | 1.1882 | 0.0747 | 61.45 | 0.0003086 | 1.426 | 0.3524 | 1.089 | 0.3184 | 1.093 |
| 1 | rs4649259 | 232007684 | G | A | 0.001992 | 0.1336 | 1.1639 | 1.1692 | 0.0112 | 77.76 | 0.00002519 | 1.446 | 0.6691 | 1.037 | 0.4087 | 1.07 |
| 9 | rs935466* | 87308057 | T | C | 0.002003 | 0.002003 | 1.1519 | 1.1519 | 0.8818 | 0 | 0.07247 | 1.162 | 0.1679 | 1.116 | 0.03102 | 1.177 |
| 20 | rs1810636 | 2602925 | T | G | 0.002306 | 0.1735 | 0.8677 | 0.8629 | 0.0046 | 81.43 | 0.0001105 | 0.7173 | 0.521 | 1.053 | 0.02929 | 0.8468 |
| 1 | rs3014578 | 239698430 | C | T | 0.002438 | 0.2093 | 1.3166 | 1.3218 | 0.0025 | 83.32 | 0.000004004 | 2.055 | 0.4284 | 1.132 | 0.9755 | 0.9952 |
| 22 | rs5769234 | 45683168 | C | G | 0.002451 | 0.1189 | 0.8659 | 0.8584 | 0.0147 | 76.31 | 0.00004251 | 0.6997 | 0.4919 | 0.945 | 0.4997 | 0.9487 |
| 4 | rs3857116 | 24421504 | T | C | 0.002546 | 0.02184 | 0.8065 | 0.804 | 0.1694 | 43.68 | 0.002201 | 0.6704 | 0.6207 | 0.941 | 0.07605 | 0.8128 |
| 1 | rs16859609 | 232000522 | G | A | 0.003006 | 0.09756 | 1.1586 | 1.1626 | 0.0349 | 70.19 | 0.000149 | 1.399 | 0.6493 | 1.04 | 0.325 | 1.085 |
| 13 | rs4941455* | 42799471 | A | G | 0.003149 | 0.01012 | 0.8371 | 0.8347 | 0.2576 | 26.28 | 0.007338 | 0.7441 | 0.048 | 0.81 | 0.5408 | 0.9423 |
| 3 | rs3774230 | 181004332 | C | T | 0.003156 | 0.08074 | 0.8649 | 0.8597 | 0.0455 | 67.64 | 0.0002167 | 0.7182 | 0.3712 | 0.926 | 0.4952 | 0.9464 |
| 20 | rs10485515* | 14797853 | T | C | 0.003902 | 0.1517 | 0.8379 | 0.8287 | 0.0109 | 77.88 | 0.0001397 | 0.6434 | 0.7516 | 1.034 | 0.08724 | 0.8461 |
| 1 | rs9660543* | 227349555 | T | C | 0.004445 | 0.1194 | 0.8412 | 0.8319 | 0.0238 | 73.26 | 0.0001376 | 0.6488 | 0.7669 | 0.969 | 0.2983 | 0.9044 |
| 15 | rs7183242 | 64883712 | G | A | 0.004615 | 0.123 | 0.8644 | 0.8575 | 0.024 | 73.17 | 0.0003744 | 0.7092 | 0.8754 | 1.014 | 0.09053 | 0.8679 |
| 19 | rs8111948* | 33517335 | G | A | 0.004683 | 0.07585 | 1.1372 | 1.1413 | 0.0689 | 62.61 | 0.0004995 | 1.331 | 0.5892 | 1.044 | 0.323 | 1.077 |
| 2 | rs707025* | 154896769 | A | C | 0.004756 | 0.2222 | 1.1389 | 1.144 | 0.0033 | 82.48 | 0.00004406 | 1.404 | 0.53 | 0.951 | 0.1238 | 1.125 |
| 22 | rs242896 | 32765815 | G | C | 0.00485 | 0.2433 | 1.136 | 1.1463 | 0.0013 | 84.98 | 0.000004196 | 1.459 | 0.8509 | 1.015 | 0.7661 | 1.022 |
| 1 | rs6429174 | 238239018 | G | T | 0.004877 | 0.3144 | 1.1439 | 1.1429 | 0.0004 | 87.03 | 0.00006161 | 1.406 | 0.1491 | 0.885 | 0.02161 | 1.199 |
| 4 | rs7664129* | 159189132 | C | A | 0.005053 | 0.1202 | 1.2058 | 1.2128 | 0.0318 | 71 | 0.001473 | 1.471 | 0.0315 | 1.272 | 0.7228 | 0.9605 |
| 11 | rs11218350* | 120957861 | T | A | 0.005644 | 0.1218 | 1.1596 | 1.1652 | 0.0333 | 70.6 | 0.003216 | 1.329 | 0.0177 | 1.243 | 0.6784 | 0.9633 |
| 9 | rs6474838* | 14454254 | G | C | 0.00582 | 0.09449 | 1.1501 | 1.1577 | 0.0512 | 66.35 | 0.0005109 | 1.378 | 0.228 | 1.112 | 0.7881 | 1.023 |
| 12 | rs931586 | 53360703 | G | C | 0.005827 | 0.1274 | 0.8672 | 0.858 | 0.0234 | 73.38 | 0.0002579 | 0.707 | 0.1765 | 0.884 | 0.988 | 0.9987 |
| 13 | rs354789 | 57024535 | T | G | 0.005873 | 0.1376 | 1.1702 | 1.1807 | 0.0217 | 73.9 | 0.0002163 | 1.471 | 0.2008 | 1.132 | 0.9847 | 0.9982 |
| 4 | rs10446758 | 149667035 | A | G | 0.006483 | 0.3773 | 0.8833 | 0.8649 | 0 | 92.27 | 1.725E-08 | 0.6225 | 0.5159 | 0.95 | 0.2564 | 1.088 |
| 4 | rs2881373 | 159312083 | T | C | 0.006939 | 0.3176 | 1.1742 | 1.1852 | 0.0003 | 87.74 | 0.0001711 | 1.5 | 0.0076 | 1.307 | 0.1142 | 0.8528 |
| 6 | rs221752* | 165779431 | T | A | 0.007083 | 0.1158 | 0.8718 | 0.8631 | 0.0348 | 70.22 | 0.0008902 | 0.7319 | 0.0859 | 0.858 | 0.8966 | 1.011 |
| 7 | rs3807660* | 77656088 | C | T | 0.007423 | 0.08645 | 1.1284 | 1.1341 | 0.0715 | 62.09 | 0.003054 | 1.274 | 0.0549 | 1.163 | 0.9287 | 0.9934 |
| 15 | rs4842907 | 84239094 | A | C | 0.007468 | 0.1557 | 0.8329 | 0.8168 | 0.0135 | 76.75 | 0.00008734 | 0.6014 | 0.7147 | 0.958 | 0.4948 | 0.9277 |
| 9 | rs1323341* | 14443010 | A | G | 0.007826 | 0.0346 | 1.1575 | 1.1607 | 0.194 | 39.03 | 0.003543 | 1.336 | 0.2095 | 1.128 | 0.5868 | 1.051 |
| 20 | rs16986484 | 24276308 | C | T | 0.008185 | 0.3817 | 1.225 | 1.2064 | 0.0004 | 87.17 | 0.000002891 | 1.817 | 0.3877 | 0.886 | 0.5414 | 1.083 |
| 5 | rs279108* | 100110254 | C | T | 0.008521 | 0.008521 | 0.8867 | 0.8867 | 0.8595 | 0 | 0.2211 | 0.9036 | 0.2051 | 0.904 | 0.04225 | 0.8579 |
| 7 | rs5014691 | 14817686 | A | G | 0.008891 | 0.0995 | 1.1442 | 1.1513 | 0.064 | 63.61 | 0.0006974 | 1.376 | 0.425 | 1.074 | 0.6092 | 1.044 |
| 10 | rs11016976* | 131540391 | C | T | 0.009495 | 0.1759 | 1.1561 | 1.1624 | 0.0192 | 74.69 | 0.0003899 | 1.423 | 0.1573 | 1.147 | 0.7282 | 0.9678 |
| 12 | rs3858655* | 95404373 | T | G | 0.01063 | 0.2137 | 1.1743 | 1.1795 | 0.0118 | 77.5 | 0.003458 | 1.397 | 0.0133 | 1.298 | 0.3737 | 0.9089 |
| 13 | rs1283145 | 100234116 | C | T | 0.01126 | 0.1487 | 0.8087 | 0.7907 | 0.0249 | 72.92 | 0.0002325 | 0.5567 | 0.6754 | 0.939 | 0.5282 | 0.9219 |
| 3 | rs4594610* | 989666 | T | C | 0.0117 | 0.1655 | 0.7968 | 0.7765 | 0.0174 | 75.32 | 0.000165 | 0.5252 | 0.864 | 0.974 | 0.4248 | 0.8894 |
| 8 | rs12546220* | 70536282 | A | G | 0.01248 | 0.01656 | 1.1411 | 1.1415 | 0.3356 | 8.42 | 0.01021 | 1.28 | 0.5292 | 1.059 | 0.2301 | 1.111 |
| 1 | rs1359414 | 111963158 | C | T | 0.01248 | 0.2471 | 1.1387 | 1.149 | 0.0049 | 81.17 | 0.00005165 | 1.461 | 0.4821 | 1.066 | 0.8156 | 0.9802 |
| 9 | rs11999298* | 36884151 | T | C | 0.01414 | 0.01414 | 1.2557 | 1.2557 | 0.3812 | 0 | 0.04712 | 1.4 | 0.0476 | 1.368 | 0.7165 | 1.058 |
| 2 | rs559113 | 169411175 | A | T | 0.01419 | 0.2953 | 0.894 | 0.8827 | 0.0011 | 85.26 | 0.00001056 | 0.6908 | 0.6849 | 0.968 | 0.7761 | 1.021 |
| 2 | rs2008776* | 118997264 | G | A | 0.01461 | 0.2304 | 1.1166 | 1.122 | 0.0111 | 77.78 | 0.0002464 | 1.351 | 0.6365 | 0.964 | 0.2467 | 1.09 |
| 15 | rs12595616* | 89364517 | C | T | 0.01486 | 0.01486 | 1.1191 | 1.1191 | 0.9896 | 0 | 0.1965 | 1.114 | 0.1829 | 1.113 | 0.1147 | 1.129 |
| 18 | rs4939756* | 43977260 | G | C | 0.01526 | 0.1325 | 1.1536 | 1.1559 | 0.0691 | 62.58 | 0.001287 | 1.398 | 0.9371 | 1.008 | 0.336 | 1.1 |
| 16 | rs4887855 | 74989899 | T | C | 0.01576 | 0.2232 | 0.817 | 0.71 | 0 | 90.49 | 8.273E-08 | 0.3562 | 0.6159 | 0.934 | 0.9352 | 1.01 |
| 5 | rs5023585* | 109381500 | A | G | 0.01603 | 0.09689 | 1.167 | 1.17 | 0.1137 | 54 | 0.0028 | 1.402 | 0.8903 | 1.015 | 0.263 | 1.133 |
| 5 | rs7734355 | 102775829 | C | T | 0.01633 | 0.324 | 1.2716 | 1.291 | 0.0013 | 84.89 | 0.00001128 | 2.194 | 0.6023 | 0.909 | 0.5964 | 1.087 |
| 11 | rs2957761* | 24098510 | T | A | 0.01771 | 0.06593 | 0.8756 | 0.8717 | 0.1706 | 43.45 | 0.004475 | 0.7424 | 0.6175 | 0.954 | 0.3526 | 0.9179 |
| 13 | rs9600236* | 73603200 | T | C | 0.01856 | 0.08679 | 0.8582 | 0.8593 | 0.1566 | 46.06 | 0.8077 | 1.028 | 0.0225 | 0.768 | 0.04506 | 0.8067 |
| 8 | rs12545053 | 65236159 | C | T | 0.01885 | 0.218 | 0.896 | 0.8884 | 0.015 | 76.2 | 0.0002245 | 0.7295 | 0.5581 | 0.953 | 0.9953 | 1 |
| 3 | rs9873216* | 64094238 | G | A | 0.0198 | 0.1603 | 0.9002 | 0.8963 | 0.0511 | 66.38 | 0.0009381 | 0.7628 | 0.83 | 0.983 | 0.5233 | 0.9536 |
| 10 | rs17091953 | 116253478 | G | A | 0.02008 | 0.3694 | 1.1415 | 1.1548 | 0.0004 | 87.38 | 0.00000566 | 1.588 | 0.6221 | 1.05 | 0.4339 | 0.9284 |
| 18 | rs4477825 | 63210306 | C | G | 0.02061 | 0.2685 | 1.1102 | 1.1175 | 0.0073 | 79.7 | 0.0001112 | 1.373 | 0.8416 | 0.984 | 0.6169 | 1.038 |
| 2 | rs7578749 | 148038283 | A | G | 0.02254 | 0.3184 | 0.8852 | 0.8712 | 0.0013 | 84.95 | 0.000025 | 0.6614 | 0.4994 | 0.939 | 0.5327 | 1.056 |
| 7 | rs17160621 | 138268222 | T | C | 0.02435 | 0.2873 | 1.1829 | 1.184 | 0.011 | 77.84 | 0.0001641 | 1.628 | 0.9178 | 0.986 | 0.7881 | 1.034 |
| 12 | rs17375557* | 95442660 | A | G | 0.02452 | 0.3203 | 1.1503 | 1.157 | 0.0039 | 81.95 | 0.003337 | 1.393 | 0.016 | 1.287 | 0.1803 | 0.8676 |
| 12 | rs4842316 | 78498696 | G | A | 0.02589 | 0.2839 | 1.1085 | 1.1159 | 0.0076 | 79.49 | 0.0001433 | 1.376 | 0.7834 | 0.978 | 0.6218 | 1.038 |
| 12 | rs1922397* | 80507780 | T | C | 0.02639 | 0.3086 | 0.8529 | 0.8145 | 0.0004 | 87.12 | 0.00002054 | 0.5506 | 0.1991 | 0.854 | 0.2995 | 1.124 |
| 3 | rs7641152* | 194502461 | T | C | 0.0266 | 0.0266 | 0.9017 | 0.9017 | 0.5648 | 0 | 0.045 | 0.8428 | 0.5774 | 0.956 | 0.1877 | 0.904 |
| 18 | rs1372622* | 2619368 | G | A | 0.02676 | 0.02676 | 1.1311 | 1.1311 | 0.4893 | 0 | 0.02722 | 1.247 | 0.516 | 1.065 | 0.3063 | 1.099 |
| 3 | rs17021431* | 84427280 | T | G | 0.02724 | 0.2773 | 1.1649 | 1.164 | 0.0169 | 75.49 | 0.0003299 | 1.536 | 0.8204 | 0.972 | 0.6408 | 1.055 |
| 19 | rs17239559 | 59398234 | C | T | 0.02833 | 0.3125 | 0.904 | 0.8945 | 0.0032 | 82.55 | 0.0002306 | 0.7346 | 0.182 | 0.899 | 0.329 | 1.077 |
| 20 | rs214760* | 2223976 | C | T | 0.02886 | 0.02886 | 1.1373 | 1.1373 | 0.3958 | 0 | 0.04421 | 1.231 | 0.8613 | 1.018 | 0.1086 | 1.174 |
| 7 | rs1029621 | 40832234 | A | G | 0.02962 | 0.2702 | 1.1191 | 1.1306 | 0.01 | 78.31 | 0.0002065 | 1.42 | 0.6411 | 1.043 | 0.8508 | 0.9842 |
| 5 | rs4146606* | 34539764 | G | A | 0.02997 | 0.1471 | 0.9064 | 0.9059 | 0.1042 | 55.77 | 0.01468 | 0.8188 | 0.6758 | 1.034 | 0.07771 | 0.8764 |
| 13 | rs9568005* | 47594325 | T | C | 0.03014 | 0.05636 | 1.1267 | 1.1279 | 0.2692 | 23.8 | 0.03716 | 1.229 | 0.0835 | 1.178 | 0.9932 | 1.001 |
| 1 | rs1252101* | 235170243 | T | C | 0.0303 | 0.2692 | 0.9035 | 0.895 | 0.0104 | 78.12 | 0.001507 | 0.763 | 0.0861 | 0.869 | 0.3622 | 1.073 |
| 7 | rs17717216* | 28448949 | C | T | 0.03084 | 0.2643 | 0.8976 | 0.89 | 0.0132 | 76.88 | 0.000378 | 0.7197 | 0.6963 | 1.034 | 0.445 | 0.9396 |
| 21 | rs7280997* | 36580379 | T | C | 0.0314 | 0.2268 | 0.9064 | 0.8998 | 0.0261 | 72.57 | 0.000931 | 0.7591 | 0.3495 | 0.928 | 0.7384 | 1.025 |
| 12 | rs931882* | 59868562 | G | A | 0.03162 | 0.03162 | 1.107 | 1.107 | 0.4236 | 0 | 0.03424 | 1.197 | 0.1881 | 1.115 | 0.7125 | 1.029 |
| 7 | rs6943487 | 12711855 | C | G | 0.03393 | 0.3235 | 0.8968 | 0.8858 | 0.0034 | 82.42 | 0.00008139 | 0.6914 | 0.6446 | 0.959 | 0.6378 | 1.04 |
| 1 | rs10493335* | 63354702 | A | G | 0.0344 | 0.2084 | 0.9058 | 0.8994 | 0.0395 | 69.07 | 0.001087 | 0.7552 | 0.5932 | 0.958 | 0.9582 | 0.996 |
| 10 | rs17091955 | 116253976 | T | C | 0.03652 | 0.3804 | 1.1275 | 1.1403 | 0.0011 | 85.25 | 0.00002827 | 1.54 | 0.7458 | 1.033 | 0.4993 | 0.9375 |
| 18 | rs9950038* | 24262559 | C | A | 0.03702 | 0.03702 | 1.0989 | 1.0989 | 0.3967 | 0 | 0.02703 | 1.2 | 0.7031 | 1.03 | 0.2769 | 1.084 |
| 3 | rs4687889* | 119020129 | G | A | 0.03889 | 0.2223 | 0.911 | 0.9064 | 0.0421 | 68.43 | 0.001215 | 0.7668 | 0.9181 | 0.992 | 0.7037 | 0.9721 |
| 13 | rs914480* | 73830626 | A | G | 0.04043 | 0.1762 | 1.0987 | 1.1037 | 0.0807 | 60.27 | 0.01855 | 1.217 | 0.0644 | 1.159 | 0.6068 | 0.9617 |
| 3 | rs421653* | 32486654 | T | G | 0.04117 | 0.3282 | 1.0983 | 1.1045 | 0.0075 | 79.55 | 0.0002099 | 1.359 | 0.7109 | 0.971 | 0.7327 | 1.026 |
| 1 | rs4656349 | 160316448 | G | A | 0.0492 | 0.4032 | 1.0961 | 1.1078 | 0.001 | 85.46 | 0.0002444 | 1.363 | 0.1513 | 1.123 | 0.1433 | 0.893 |
| 8 | rs10104759* | 35423940 | G | T | 0.05173 | 0.05173 | 0.9122 | 0.9122 | 0.4715 | 0 | 0.03687 | 0.8351 | 0.4568 | 0.941 | 0.5353 | 0.9529 |
| 7 | rs1735090* | 152626909 | A | G | 0.05197 | 0.2877 | 1.1311 | 1.139 | 0.0241 | 73.15 | 0.002747 | 1.401 | 0.1938 | 1.155 | 0.4302 | 0.92 |
| 15 | rs17738626* | 60613489 | T | C | 0.05465 | 0.1753 | 1.1647 | 1.1589 | 0.1538 | 46.58 | 0.9153 | 0.9845 | 0.5036 | 1.094 | 0.007968 | 1.419 |
| 1 | rs10157868* | 96049209 | A | G | 0.05731 | 0.3441 | 1.101 | 1.1118 | 0.0076 | 79.52 | 0.0008479 | 1.358 | 0.2467 | 1.107 | 0.3256 | 0.9212 |
| 5 | rs2416248 | 110206705 | C | T | 0.05983 | 0.4261 | 1.1038 | 1.1102 | 0.0019 | 84.01 | 0.0001892 | 1.423 | 0.2285 | 0.896 | 0.3942 | 1.077 |
| 5 | rs6888588 | 110225865 | G | A | 0.06054 | 0.4263 | 1.1035 | 1.1089 | 0.0022 | 83.66 | 0.0002496 | 1.412 | 0.2139 | 0.893 | 0.3477 | 1.085 |
| 6 | rs3892789* | 111403162 | G | A | 0.06084 | 0.1673 | 1.1191 | 1.1197 | 0.156 | 46.18 | 0.01083 | 1.31 | 0.8927 | 0.986 | 0.3946 | 1.089 |
| 11 | rs538645 | 118216279 | G | A | 0.06229 | 0.3862 | 1.1058 | 1.1145 | 0.0047 | 81.34 | 0.0001778 | 1.441 | 0.6394 | 0.957 | 0.9094 | 1.01 |
| 2 | rs6730095 | 77151964 | A | C | 0.06976 | 0.4175 | 1.0935 | 1.1006 | 0.0032 | 82.58 | 0.0001318 | 1.399 | 0.9485 | 0.994 | 0.6366 | 0.9623 |
| 5 | rs16879116 | 7847503 | G | A | 0.07127 | 0.4925 | 0.9185 | 0.9053 | 0.0001 | 89.41 | 0.00001086 | 0.6855 | 0.5443 | 0.952 | 0.1143 | 1.131 |
| 17 | rs2173202* | 36488761 | T | C | 0.07146 | 0.2949 | 1.086 | 1.0897 | 0.0407 | 68.76 | 0.002886 | 1.28 | 0.6196 | 0.961 | 0.4678 | 1.057 |
| 11 | rs11020505* | 93117781 | A | C | 0.07308 | 0.07308 | 0.9205 | 0.9205 | 0.9007 | 0 | 0.4021 | 0.9319 | 0.4296 | 0.939 | 0.1477 | 0.8956 |
| 6 | rs1499571* | 119871783 | T | C | 0.07445 | 0.07445 | 0.9208 | 0.9208 | 0.7346 | 0 | 0.1638 | 0.8891 | 0.2004 | 0.902 | 0.6389 | 0.9649 |
| 14 | rs7161259* | 91214828 | T | G | 0.07759 | 0.07759 | 0.9225 | 0.9225 | 0.4578 | 0 | 0.05751 | 0.8543 | 0.3131 | 0.923 | 0.8194 | 0.9829 |
| 2 | rs16831315* | 135541544 | G | A | 0.08056 | 0.323 | 1.1201 | 1.1242 | 0.0359 | 69.95 | 0.005087 | 1.378 | 0.2671 | 1.133 | 0.4169 | 0.9143 |
| 6 | rs7774757* | 155417681 | C | A | 0.08216 | 0.1845 | 1.0828 | 1.0858 | 0.1601 | 45.42 | 0.01345 | 1.227 | 0.4479 | 1.062 | 0.9182 | 0.9922 |
| 1 | rs880385* | 55592687 | G | T | 0.08402 | 0.1689 | 1.081 | 1.0822 | 0.1987 | 38.11 | 0.02169 | 1.206 | 0.8367 | 0.984 | 0.3359 | 1.074 |
| 6 | rs7759835* | 9529407 | T | C | 0.08692 | 0.1098 | 0.9133 | 0.9131 | 0.3164 | 13.1 | 0.9341 | 0.9922 | 0.0307 | 0.815 | 0.4535 | 0.9375 |
| 9 | rs6478690 | 126797131 | C | T | 0.08829 | 0.4366 | 1.081 | 1.0925 | 0.0021 | 83.83 | 0.0001707 | 1.367 | 0.6734 | 1.034 | 0.3236 | 0.9284 |
| 1 | rs4659986* | 238866166 | C | T | 0.09127 | 0.09127 | 1.0872 | 1.0872 | 0.5608 | 0 | 0.06657 | 1.177 | 0.5851 | 1.048 | 0.553 | 1.05 |
| 5 | rs13354207 | 110179690 | C | G | 0.09152 | 0.4725 | 1.0947 | 1.1035 | 0.0015 | 84.67 | 0.0001083 | 1.454 | 0.3331 | 0.914 | 0.8552 | 1.016 |
| 8 | rs10954969* | 34724264 | G | C | 0.09189 | 0.3838 | 1.1082 | 1.1117 | 0.0191 | 74.73 | 0.138 | 1.183 | 0.0073 | 1.315 | 0.2364 | 0.8861 |
| 21 | rs2824723* | 18595079 | C | T | 0.09555 | 0.2066 | 0.917 | 0.9167 | 0.1744 | 42.74 | 0.05483 | 0.8329 | 0.5939 | 1.05 | 0.1295 | 0.879 |
| 8 | rs1457461 | 135167438 | C | T | 0.09719 | 0.4676 | 1.1014 | 1.1137 | 0.0015 | 84.55 | 0.0002393 | 1.469 | 0.4656 | 1.076 | 0.1855 | 0.8793 |
| 7 | rs6463813* | 8476768 | A | G | 0.1032 | 0.1032 | 1.0769 | 1.0769 | 0.8724 | 0 | 0.2445 | 1.101 | 0.2638 | 1.092 | 0.5681 | 1.044 |
| 1 | rs17666678 | 215844513 | T | C | 0.1034 | 0.5073 | 1.0951 | 1.1088 | 0.0004 | 87.13 | 0.00007944 | 1.482 | 0.5287 | 1.064 | 0.1274 | 0.8692 |
| 20 | rs1201987* | 58736287 | A | G | 0.1053 | 0.1053 | 0.923 | 0.923 | 0.5585 | 0 | 0.4852 | 0.9392 | 0.806 | 0.979 | 0.07166 | 0.8638 |
| 14 | rs7140637* | 49892268 | C | G | 0.1062 | 0.1451 | 1.0813 | 1.0814 | 0.2916 | 18.86 | 0.05636 | 1.179 | 0.7914 | 0.978 | 0.2449 | 1.099 |
| 4 | rs11727321* | 43344913 | T | G | 0.1073 | 0.138 | 0.8539 | 0.8539 | 0.3083 | 15.01 | 0.1428 | 0.7671 | 0.7522 | 1.055 | 0.0988 | 0.7673 |
| 10 | rs2804869* | 33856365 | A | G | 0.1111 | 0.1111 | 1.0763 | 1.0763 | 0.8358 | 0 | 0.1802 | 1.118 | 0.375 | 1.074 | 0.5629 | 1.045 |
| 7 | rs2215595* | 28424667 | A | G | 0.1115 | 0.3345 | 0.9241 | 0.9163 | 0.0364 | 69.83 | 0.002658 | 0.757 | 0.7602 | 1.026 | 0.7899 | 0.9787 |
| 15 | rs1870301* | 93412015 | T | C | 0.1159 | 0.3831 | 1.1028 | 1.1197 | 0.0135 | 76.77 | 0.01058 | 1.344 | 0.0849 | 1.204 | 0.2023 | 0.8793 |
| 3 | rs9289008* | 116302696 | G | A | 0.1168 | 0.3369 | 1.0817 | 1.0854 | 0.055 | 65.52 | 0.01677 | 1.236 | 0.1885 | 1.122 | 0.367 | 0.9268 |
| 2 | rs17334527* | 77117777 | C | A | 0.1193 | 0.3474 | 1.0995 | 1.0999 | 0.0633 | 63.76 | 0.005023 | 1.347 | 0.7711 | 0.969 | 0.8433 | 1.02 |
| 16 | rs2055846* | 61733578 | T | C | 0.1211 | 0.1211 | 0.9273 | 0.9273 | 0.3684 | 0 | 0.1253 | 0.8737 | 0.795 | 1.022 | 0.1588 | 0.8927 |
| 3 | rs794184 | 4426994 | C | T | 0.1283 | 0.5017 | 0.9309 | 0.9217 | 0.0013 | 84.91 | 0.000119 | 0.7185 | 0.3722 | 1.075 | 0.9232 | 1.008 |
| 2 | rs17499330* | 118231401 | T | C | 0.1292 | 0.1292 | 1.1017 | 1.1017 | 0.3752 | 0 | 0.1526 | 1.178 | 0.7868 | 0.971 | 0.1412 | 1.169 |
| 6 | rs2743937* | 29915902 | C | T | 0.1332 | 0.2772 | 0.9258 | 0.9213 | 0.1169 | 53.41 | 0.02949 | 0.8149 | 0.2315 | 0.899 | 0.5362 | 1.054 |
| 3 | rs2979378* | 126779879 | G | T | 0.1336 | 0.1883 | 1.0703 | 1.069 | 0.286 | 20.12 | 0.9523 | 0.9951 | 0.6851 | 1.032 | 0.03256 | 1.173 |
| 12 | rs1521386* | 77418817 | G | A | 0.1359 | 0.5774 | 1.0901 | 1.0895 | 0.0009 | 85.83 | 0.000119 | 1.475 | 0.2026 | 0.876 | 0.9942 | 1.001 |
| 12 | rs2710901 | 77433629 | G | A | 0.1456 | 0.5887 | 1.0881 | 1.0879 | 0.0008 | 86.08 | 0.00009768 | 1.483 | 0.2419 | 0.884 | 0.8441 | 0.9814 |
| 11 | rs10488779* | 79494759 | C | T | 0.1525 | 0.1525 | 0.9274 | 0.9274 | 0.4344 | 0 | 0.08368 | 0.8472 | 0.9481 | 1.006 | 0.3953 | 0.9291 |
| 3 | rs3774108* | 10898193 | C | A | 0.1587 | 0.4381 | 1.0795 | 1.0806 | 0.034 | 70.43 | 0.00458 | 1.314 | 0.4487 | 0.929 | 0.7005 | 1.035 |
| 6 | rs6908632* | 17505095 | C | T | 0.1617 | 0.3398 | 1.0714 | 1.071 | 0.1201 | 52.82 | 0.1437 | 1.139 | 0.3845 | 0.928 | 0.06938 | 1.161 |
| 14 | rs10141804* | 70318983 | T | C | 0.162 | 0.3925 | 1.0712 | 1.0776 | 0.0428 | 68.27 | 0.03925 | 1.2 | 0.0975 | 1.153 | 0.256 | 0.9118 |
| 4 | rs17293620* | 184539543 | T | G | 0.1631 | 0.2619 | 0.9136 | 0.9073 | 0.1691 | 43.73 | 0.02463 | 0.761 | 0.5237 | 0.93 | 0.7969 | 1.027 |
| 3 | rs13314127* | 189952658 | T | C | 0.1635 | 0.1635 | 0.9328 | 0.9328 | 0.5739 | 0 | 0.1065 | 0.8644 | 0.5306 | 0.947 | 0.8158 | 0.9809 |
| 8 | rs10957988* | 81983149 | T | C | 0.1662 | 0.1662 | 0.9372 | 0.9372 | 0.8376 | 0 | 0.367 | 0.9259 | 0.2522 | 0.911 | 0.7024 | 0.971 |
| 3 | rs11718847* | 43026662 | A | T | 0.1686 | 0.1686 | 1.1021 | 1.1021 | 0.3968 | 0 | 0.3834 | 1.115 | 0.8356 | 0.975 | 0.08671 | 1.23 |
| 3 | rs6445058* | 173498694 | A | C | 0.1742 | 0.2133 | 0.9405 | 0.941 | 0.3096 | 14.72 | 0.4604 | 0.9414 | 0.7458 | 1.026 | 0.05994 | 0.8692 |
| 6 | rs6934412* | 85119973 | T | C | 0.175 | 0.175 | 0.9351 | 0.9351 | 0.4044 | 0 | 0.1229 | 0.8702 | 0.2674 | 0.909 | 0.8319 | 1.017 |
| 2 | rs4973006* | 228455120 | C | A | 0.1764 | 0.4832 | 0.9406 | 0.9366 | 0.0145 | 76.38 | 0.005049 | 0.7923 | 0.2054 | 1.105 | 0.3607 | 0.9342 |
| 12 | rs2731402* | 59992544 | C | T | 0.1796 | 0.1796 | 0.9327 | 0.9327 | 0.5288 | 0 | 0.8877 | 1.013 | 0.3867 | 0.924 | 0.1287 | 0.8784 |
| 8 | rs4373539* | 5063717 | C | T | 0.1837 | 0.2597 | 1.0728 | 1.0756 | 0.2252 | 32.93 | 0.03152 | 1.233 | 0.7227 | 1.033 | 0.9386 | 0.9933 |
| 14 | rs8012146* | 62215852 | A | C | 0.1867 | 0.4733 | 0.9339 | 0.9227 | 0.0095 | 78.53 | 0.001365 | 0.7328 | 0.3696 | 1.082 | 0.8128 | 0.9801 |
| 3 | rs4373099 | 54254022 | T | C | 0.1896 | 0.5774 | 1.0632 | 1.0761 | 0.0004 | 87.36 | 0.0001069 | 1.387 | 0.8021 | 1.021 | 0.111 | 0.8845 |
| 6 | rs1015340* | 144077050 | C | T | 0.191 | 0.191 | 1.0608 | 1.0608 | 0.602 | 0 | 0.8643 | 1.014 | 0.6558 | 1.036 | 0.114 | 1.125 |
| 4 | rs1961460* | 82393522 | T | C | 0.1931 | 0.4551 | 0.938 | 0.9371 | 0.0443 | 67.91 | 0.04495 | 0.835 | 0.2106 | 1.113 | 0.1264 | 0.8837 |
| 4 | rs1383421* | 80154502 | G | T | 0.1934 | 0.1934 | 0.8877 | 0.8877 | 0.3753 | 0 | 0.6405 | 0.9254 | 0.063 | 0.734 | 0.9683 | 0.9942 |
| 3 | rs10804515* | 116184747 | T | C | 0.1961 | 0.4561 | 1.0691 | 1.0725 | 0.0369 | 69.69 | 0.06256 | 1.186 | 0.078 | 1.171 | 0.1914 | 0.892 |
| 7 | rs10263677* | 40578775 | G | A | 0.2003 | 0.5167 | 1.0969 | 1.105 | 0.0107 | 77.97 | 0.001442 | 1.501 | 0.9909 | 0.999 | 0.4087 | 0.9057 |
| 13 | rs1023102* | 72723017 | C | T | 0.2054 | 0.2054 | 1.059 | 1.059 | 0.5552 | 0 | 0.1478 | 1.126 | 0.9543 | 0.996 | 0.4032 | 1.064 |
| 16 | rs12933414* | 9401468 | G | A | 0.2144 | 0.4113 | 0.9453 | 0.9401 | 0.0645 | 63.53 | 0.02541 | 0.8316 | 0.2947 | 0.921 | 0.33 | 1.075 |
| 4 | rs17553672 | 24448662 | G | T | 0.2154 | 0.2154 | 1.0667 | 1.0667 | 0.8281 | 0 | 0.2479 | 1.115 | 0.5038 | 1.063 | 0.728 | 1.031 |
| 1 | rs4653300* | 37796641 | A | G | 0.216 | 0.216 | 1.0575 | 1.0575 | 0.4497 | 0 | 0.9206 | 1.008 | 0.8256 | 1.017 | 0.08004 | 1.14 |
| 6 | rs1015887* | 144077451 | A | G | 0.2207 | 0.2207 | 1.0573 | 1.0573 | 0.8009 | 0 | 0.7138 | 1.031 | 0.6652 | 1.035 | 0.2036 | 1.1 |
| 8 | rs2588223* | 17431383 | T | A | 0.2211 | 0.55 | 1.057 | 1.0627 | 0.0066 | 80.11 | 0.001295 | 1.302 | 0.2712 | 0.917 | 0.8915 | 1.01 |
| 10 | rs7087228* | 8304160 | T | C | 0.2317 | 0.4617 | 0.9432 | 0.9362 | 0.0353 | 70.09 | 0.004908 | 0.7755 | 0.6421 | 1.04 | 0.9274 | 1.007 |
| 6 | rs2800708* | 127479310 | G | A | 0.2419 | 0.2837 | 0.9485 | 0.9485 | 0.3037 | 16.09 | 0.1532 | 0.8892 | 0.5634 | 1.046 | 0.2392 | 0.9161 |
| 3 | rs17233898* | 5807331 | T | C | 0.242 | 0.4698 | 0.9304 | 0.92 | 0.0311 | 71.18 | 0.005999 | 0.7271 | 0.4085 | 1.091 | 0.7392 | 0.9669 |
| 17 | rs10852877* | 6230343 | T | C | 0.2446 | 0.3704 | 1.074 | 1.0758 | 0.1712 | 43.35 | 0.03121 | 1.266 | 0.6859 | 0.958 | 0.7452 | 1.034 |
| 2 | rs1227921* | 162400446 | C | G | 0.2462 | 0.2827 | 1.0565 | 1.0564 | 0.3131 | 13.88 | 0.1607 | 1.126 | 0.5657 | 0.954 | 0.2411 | 1.097 |
| 6 | rs9464886* | 16495782 | G | A | 0.2492 | 0.2492 | 1.0675 | 1.0675 | 0.4527 | 0 | 0.2065 | 1.137 | 0.269 | 1.114 | 0.749 | 0.9699 |
| 16 | rs4426363* | 78595960 | A | G | 0.2496 | 0.5212 | 1.0623 | 1.0717 | 0.0149 | 76.22 | 0.003307 | 1.322 | 0.7861 | 1.025 | 0.3069 | 0.9157 |
| 9 | rs7045215* | 104739847 | G | C | 0.2505 | 0.2636 | 1.0586 | 1.0588 | 0.3458 | 5.82 | 0.06769 | 1.176 | 0.9265 | 0.992 | 0.7664 | 1.025 |
| 10 | rs10794730* | 1237022 | T | C | 0.2515 | 0.348 | 1.0588 | 1.0622 | 0.1905 | 39.68 | 0.09389 | 1.164 | 0.2503 | 1.106 | 0.4805 | 0.944 |
| 5 | rs10038955* | 155681482 | G | T | 0.2526 | 0.2526 | 1.057 | 1.057 | 0.4312 | 0 | 0.09108 | 1.159 | 0.9684 | 0.997 | 0.6911 | 1.032 |
| 15 | rs17191351* | 58418368 | T | C | 0.2534 | 0.5223 | 1.0798 | 1.0783 | 0.0465 | 67.4 | 0.007429 | 1.36 | 0.8102 | 0.971 | 0.6294 | 0.9472 |
| 9 | rs1329393* | 97358747 | T | C | 0.2574 | 0.4372 | 0.9342 | 0.9302 | 0.0926 | 57.98 | 0.0362 | 0.7887 | 0.3534 | 1.101 | 0.3675 | 0.9166 |
| 3 | rs6769328* | 79455526 | C | T | 0.2585 | 0.2585 | 0.9479 | 0.9479 | 0.8938 | 0 | 0.8173 | 0.9803 | 0.4467 | 0.939 | 0.3513 | 0.9304 |
| 12 | rs2114862* | 105120036 | G | C | 0.2588 | 0.2588 | 0.9376 | 0.9376 | 0.7056 | 0 | 0.2076 | 0.877 | 0.8977 | 0.988 | 0.5449 | 0.9442 |
| 21 | rs189900* | 23322128 | G | A | 0.2599 | 0.4469 | 0.9503 | 0.9532 | 0.1447 | 48.28 | 0.3749 | 1.075 | 0.436 | 0.94 | 0.05282 | 0.8653 |
| 1 | rs2205848* | 167942917 | C | T | 0.2612 | 0.2612 | 1.0583 | 1.0583 | 0.4729 | 0 | 0.9724 | 1.003 | 0.101 | 1.154 | 0.7977 | 1.022 |
| 8 | rs4737291* | 70924348 | C | T | 0.2658 | 0.2658 | 1.0588 | 1.0588 | 0.4475 | 0 | 0.09565 | 1.168 | 0.9325 | 1.008 | 0.8148 | 1.02 |
| 1 | rs10800533* | 160683253 | A | G | 0.2735 | 0.2735 | 0.9352 | 0.9352 | 0.6432 | 0 | 0.2413 | 0.8766 | 0.9231 | 1.01 | 0.4032 | 0.9196 |
| 6 | rs9322458* | 154635053 | A | T | 0.2759 | 0.4614 | 1.0699 | 1.0779 | 0.0681 | 62.78 | 0.01857 | 1.303 | 0.5729 | 1.063 | 0.3904 | 0.9162 |
| 11 | rs10501659* | 87513536 | T | C | 0.281 | 0.4811 | 1.0536 | 1.0605 | 0.0525 | 66.06 | 0.01675 | 1.234 | 0.5295 | 1.055 | 0.3276 | 0.9251 |
| 15 | rs1989658* | 23376708 | G | T | 0.2948 | 0.4367 | 1.051 | 1.0531 | 0.1402 | 49.1 | 0.02971 | 1.204 | 0.6066 | 0.959 | 0.8083 | 1.019 |
| 13 | rs9564316* | 33884770 | A | T | 0.2998 | 0.2998 | 0.9428 | 0.9428 | 0.5894 | 0 | 0.1543 | 0.8614 | 0.7852 | 0.973 | 0.8591 | 0.9838 |
| 4 | rs7657701 | 182529026 | C | T | 0.3076 | 0.5688 | 0.9217 | 0.8824 | 0.0006 | 86.52 | 0.0001247 | 0.5491 | 0.2378 | 1.174 | 0.7501 | 1.041 |
| 11 | rs1474844* | 32266552 | G | A | 0.3077 | 0.3077 | 0.9517 | 0.9517 | 0.4802 | 0 | 0.1214 | 0.8737 | 0.7435 | 0.973 | 0.9643 | 1.004 |
| 8 | rs2975696* | 10150116 | A | C | 0.3087 | 0.6189 | 0.9509 | 0.9398 | 0.0018 | 84.21 | 0.0005182 | 0.7283 | 0.1965 | 1.116 | 0.867 | 1.014 |
| 7 | rs10953515* | 106080918 | T | C | 0.3136 | 0.3136 | 1.05 | 1.05 | 0.5218 | 0 | 0.8323 | 0.9814 | 0.1543 | 1.126 | 0.6259 | 1.04 |
| 7 | rs17133918* | 50679766 | T | C | 0.3194 | 0.5721 | 0.9537 | 0.959 | 0.0899 | 58.49 | 0.5055 | 1.06 | 0.9266 | 1.008 | 0.02057 | 0.835 |
| 5 | rs10064352* | 75512971 | A | G | 0.3208 | 0.519 | 1.0466 | 1.0524 | 0.0509 | 66.41 | 0.02729 | 1.201 | 0.4064 | 1.069 | 0.2436 | 0.9155 |
| 8 | rs4292660* | 99865472 | G | A | 0.3322 | 0.3322 | 0.9556 | 0.9556 | 0.9345 | 0 | 0.4076 | 0.9322 | 0.7335 | 0.973 | 0.6001 | 0.9604 |
| 2 | rs11892271* | 52237202 | A | G | 0.3326 | 0.5438 | 1.0819 | 1.0981 | 0.0283 | 71.95 | 0.005327 | 1.521 | 0.6016 | 0.928 | 0.7236 | 0.9552 |
| 6 | rs4709680* | 163520791 | C | T | 0.338 | 0.5426 | 0.9511 | 0.9546 | 0.1195 | 52.94 | 0.9831 | 1.002 | 0.538 | 1.058 | 0.02862 | 0.828 |
| 2 | rs565394* | 21259094 | G | A | 0.3423 | 0.4407 | 0.958 | 0.9553 | 0.1799 | 41.71 | 0.07889 | 0.8659 | 0.4256 | 0.939 | 0.4327 | 1.06 |
| 10 | rs6602686* | 13904269 | A | G | 0.3474 | 0.3515 | 1.0446 | 1.0448 | 0.36 | 2.13 | 0.09864 | 1.149 | 0.7142 | 1.03 | 0.7726 | 0.9781 |
| 10 | rs12354899* | 86977616 | C | G | 0.3504 | 0.4891 | 1.0461 | 1.0482 | 0.1381 | 49.5 | 0.4914 | 1.062 | 0.0583 | 1.171 | 0.3742 | 0.9313 |
| 2 | rs10932541* | 215099110 | T | C | 0.3588 | 0.75 | 1.0501 | 1.0387 | 0.0069 | 79.92 | 0.09758 | 0.8479 | 0.0046 | 1.288 | 0.8466 | 1.017 |
| 15 | rs8038473* | 96097480 | G | A | 0.3608 | 0.4279 | 1.044 | 1.0457 | 0.24 | 29.92 | 0.1182 | 1.142 | 0.3959 | 1.072 | 0.4654 | 0.9444 |
| 9 | rs7856164* | 110574103 | G | T | 0.3612 | 0.7387 | 1.0714 | 1.0558 | 0.0099 | 78.33 | 0.1706 | 0.8242 | 0.9013 | 0.984 | 0.004126 | 1.432 |
| 9 | rs10759949* | 119632012 | C | A | 0.3752 | 0.4452 | 1.0408 | 1.0396 | 0.281 | 21.23 | 0.6221 | 0.9605 | 0.8616 | 1.014 | 0.08162 | 1.139 |
| 3 | rs4234541 | 17157701 | C | T | 0.38 | 0.7386 | 1.0405 | 1.0465 | 0.0001 | 88.94 | 0.0002345 | 1.351 | 0.0214 | 0.834 | 0.7893 | 1.02 |
| 15 | rs4146472* | 62087994 | G | A | 0.382 | 0.382 | 0.9484 | 0.9484 | 0.6954 | 0 | 0.942 | 1.008 | 0.7246 | 0.963 | 0.2431 | 0.8914 |
| 9 | rs10123421* | 76903099 | C | T | 0.3851 | 0.6138 | 0.9538 | 0.9451 | 0.0147 | 76.29 | 0.05995 | 0.8304 | 0.1121 | 0.86 | 0.07684 | 1.172 |
| 12 | rs782103* | 57122898 | T | C | 0.3886 | 0.5418 | 1.0435 | 1.0465 | 0.1029 | 56.03 | 0.02459 | 1.219 | 0.8608 | 0.985 | 0.6288 | 0.9612 |
| 9 | rs10817664* | 116472734 | A | C | 0.3946 | 0.3946 | 1.0446 | 1.0446 | 0.8281 | 0 | 0.3558 | 1.088 | 0.945 | 1.006 | 0.6213 | 1.044 |
| 6 | rs2181011* | 70896157 | C | T | 0.3987 | 0.3987 | 1.039 | 1.039 | 0.6967 | 0 | 0.9961 | 1 | 0.2445 | 1.096 | 0.7713 | 1.022 |
| 9 | rs10818226* | 120523994 | G | C | 0.3995 | 0.5426 | 1.0462 | 1.049 | 0.1176 | 53.28 | 0.02901 | 1.234 | 0.6774 | 0.961 | 0.8191 | 0.9802 |
| 13 | rs4408437* | 57133947 | C | T | 0.4003 | 0.6943 | 1.0512 | 1.0564 | 0.004 | 81.91 | 0.003573 | 1.364 | 0.0755 | 0.833 | 0.6799 | 1.042 |
| 2 | rs10173668* | 192344408 | T | G | 0.4065 | 0.5623 | 1.0396 | 1.0363 | 0.1778 | 42.1 | 0.4958 | 0.9432 | 0.9464 | 1.005 | 0.05552 | 1.159 |
| 6 | rs7762948* | 34094069 | A | G | 0.4098 | 0.4098 | 1.0779 | 1.0779 | 0.7502 | 0 | 0.2824 | 1.189 | 0.9683 | 1.007 | 0.7476 | 1.049 |
| 1 | rs7518650* | 215538497 | G | A | 0.414 | 0.6423 | 0.9392 | 0.9196 | 0.0041 | 81.77 | 0.001128 | 0.6291 | 0.477 | 1.099 | 0.4106 | 1.108 |
| 16 | rs1836872* | 62621225 | A | G | 0.4216 | 0.4427 | 1.063 | 1.0633 | 0.3311 | 9.53 | 0.09288 | 1.252 | 0.9453 | 0.991 | 0.852 | 0.9762 |
| 4 | rs12509776* | 116608823 | T | C | 0.4397 | 0.4411 | 0.9591 | 0.9591 | 0.3648 | 0.84 | 0.216 | 0.8853 | 0.4076 | 0.925 | 0.5303 | 1.058 |
| 2 | rs6738950* | 227755364 | T | C | 0.4468 | 0.6722 | 1.0518 | 1.0463 | 0.0745 | 61.5 | 0.2535 | 0.8729 | 0.8035 | 1.029 | 0.03566 | 1.263 |
| 12 | rs6538271* | 90335642 | G | A | 0.448 | 0.448 | 1.0584 | 1.0584 | 0.6834 | 0 | 0.9454 | 1.009 | 0.2491 | 1.162 | 0.9135 | 1.013 |
| 12 | rs11116324* | 83254244 | T | C | 0.4483 | 0.7989 | 1.0383 | 1.0276 | 0.01 | 78.27 | 0.03789 | 0.8271 | 0.2788 | 1.096 | 0.03737 | 1.186 |
| 5 | rs11745313* | 147552456 | G | A | 0.4484 | 0.6545 | 0.9659 | 0.963 | 0.0345 | 70.3 | 0.02726 | 0.8311 | 0.153 | 1.121 | 0.5353 | 0.9544 |
| 3 | rs1348213* | 77398339 | A | G | 0.4539 | 0.6041 | 0.9658 | 0.9626 | 0.0824 | 59.95 | 0.02751 | 0.8298 | 0.4018 | 1.07 | 0.9719 | 0.9973 |
| 7 | rs1468244* | 11431856 | T | C | 0.4584 | 0.6604 | 0.9658 | 0.9699 | 0.1114 | 54.44 | 0.1787 | 1.122 | 0.2812 | 0.915 | 0.1599 | 0.8978 |
| 8 | rs752036* | 101695417 | A | G | 0.4665 | 0.5302 | 0.9668 | 0.9668 | 0.2611 | 25.53 | 0.2853 | 0.9123 | 0.3633 | 1.076 | 0.2658 | 0.9193 |
| 5 | rs4380636* | 93203859 | A | G | 0.4723 | 0.8253 | 1.0695 | 1.0388 | 0.0351 | 70.15 | 0.06894 | 0.7155 | 0.3669 | 1.15 | 0.07629 | 1.306 |
| 18 | rs1941065* | 10012266 | T | A | 0.479 | 0.7804 | 1.0439 | 1.0431 | 0.002 | 83.95 | 0.002109 | 1.385 | 0.0585 | 0.816 | 0.9649 | 1.004 |
| 4 | rs7656584* | 137628031 | A | G | 0.4799 | 0.4799 | 0.962 | 0.962 | 0.7953 | 0 | 0.6114 | 0.9509 | 0.4047 | 0.922 | 0.9466 | 1.006 |
| 13 | rs1885952* | 18548695 | C | T | 0.4817 | 0.5707 | 0.9629 | 0.9633 | 0.2218 | 33.59 | 0.4336 | 1.08 | 0.0949 | 0.853 | 0.7515 | 0.9728 |
| 3 | rs17746541* | 60354293 | C | A | 0.4896 | 0.4896 | 1.0326 | 1.0326 | 0.7418 | 0 | 0.8776 | 1.013 | 0.304 | 1.086 | 0.9827 | 1.002 |
| 2 | rs3770542* | 216555396 | A | T | 0.5003 | 0.5003 | 0.9581 | 0.9581 | 0.6359 | 0 | 0.4091 | 0.9082 | 0.4511 | 0.92 | 0.7422 | 1.035 |
| 3 | rs12695382* | 120430861 | G | A | 0.5057 | 0.6651 | 1.0483 | 1.0482 | 0.0949 | 57.53 | 0.0372 | 1.293 | 0.362 | 0.892 | 0.9857 | 0.9979 |
| 5 | rs1428558* | 73887534 | T | C | 0.5065 | 0.6297 | 1.0308 | 1.0362 | 0.0747 | 61.46 | 0.07121 | 1.162 | 0.4149 | 1.067 | 0.1906 | 0.9061 |
| 3 | rs4419372* | 61580831 | C | T | 0.5094 | 0.5915 | 0.9642 | 0.959 | 0.1382 | 49.48 | 0.04184 | 0.8104 | 0.6937 | 1.038 | 0.7424 | 1.03 |
| 5 | rs10516147* | 178027090 | G | A | 0.5166 | 0.5166 | 0.9646 | 0.9646 | 0.5769 | 0 | 0.3106 | 0.9018 | 0.6724 | 1.042 | 0.5769 | 0.9505 |
| 6 | rs9368394* | 22132981 | T | C | 0.5264 | 0.6085 | 0.9416 | 0.9128 | 0.0317 | 71.03 | 0.009067 | 0.6144 | 0.6137 | 1.086 | 0.553 | 1.092 |
| 10 | rs2474568* | 38423153 | T | A | 0.5266 | 0.6436 | 0.9715 | 0.9739 | 0.2092 | 36.08 | 0.401 | 1.072 | 0.8962 | 0.99 | 0.09369 | 0.8818 |
| 9 | rs11795150* | 16095961 | C | A | 0.5286 | 0.6995 | 0.9713 | 0.9632 | 0.0122 | 77.31 | 0.004625 | 0.787 | 0.5143 | 1.054 | 0.3814 | 1.069 |
| 12 | rs2710922 | 77423482 | G | A | 0.5298 | 0.7748 | 1.0484 | 1.0631 | 0.0003 | 87.5 | 0.0003613 | 1.599 | 0.7279 | 0.952 | 0.05387 | 0.7927 |
| 4 | rs12645979* | 11477195 | G | A | 0.5413 | 0.6127 | 1.0281 | 1.0291 | 0.2124 | 35.46 | 0.8617 | 1.014 | 0.09 | 1.143 | 0.448 | 0.9444 |
| 8 | rs16938580* | 74249204 | A | G | 0.5569 | 0.5569 | 1.0375 | 1.0375 | 0.7059 | 0 | 0.4714 | 1.084 | 0.7268 | 0.962 | 0.5236 | 1.068 |
| 5 | rs1295212* | 154965952 | T | C | 0.5628 | 0.5628 | 1.027 | 1.027 | 0.5624 | 0 | 0.6793 | 0.966 | 0.2652 | 1.093 | 0.7826 | 1.021 |
| 8 | rs1845040* | 19612672 | G | A | 0.5645 | 0.7008 | 0.9739 | 0.9683 | 0.0361 | 69.9 | 0.01344 | 0.8134 | 0.5174 | 1.053 | 0.4987 | 1.052 |
| 10 | rs1665571* | 80081635 | G | A | 0.5755 | 0.6066 | 1.03 | 1.032 | 0.2621 | 25.33 | 0.1661 | 1.143 | 0.5665 | 1.054 | 0.3879 | 0.9279 |
| 18 | rs12961718* | 65238984 | G | A | 0.5832 | 0.5832 | 1.0267 | 1.0267 | 0.6585 | 0 | 0.5857 | 1.049 | 0.4094 | 1.071 | 0.6988 | 0.9697 |
| 6 | rs4707795 | 94111589 | T | C | 0.5915 | 0.7113 | 1.0339 | 1.0359 | 0.0956 | 57.41 | 0.4046 | 1.097 | 0.1235 | 1.178 | 0.1639 | 0.8639 |
| 3 | rs9846736* | 31586782 | A | G | 0.6019 | 0.7659 | 0.9726 | 0.962 | 0.0026 | 83.15 | 0.002 | 0.7414 | 0.6922 | 1.037 | 0.1149 | 1.15 |
| 1 | rs4650704* | 173385546 | G | A | 0.602 | 0.7384 | 1.0308 | 1.0381 | 0.025 | 72.89 | 0.02173 | 1.269 | 0.7814 | 1.029 | 0.1272 | 0.8632 |
| 2 | rs4663128* | 234881472 | C | A | 0.6041 | 0.6041 | 0.9759 | 0.9759 | 0.7328 | 0 | 0.4262 | 0.9341 | 0.668 | 0.965 | 0.7878 | 1.021 |
| 18 | rs4377227* | 20987826 | C | G | 0.6137 | 0.6137 | 0.9775 | 0.9775 | 0.5408 | 0 | 0.8477 | 1.016 | 0.8514 | 1.015 | 0.2349 | 0.9153 |
| 9 | rs10758941* | 8126797 | C | T | 0.6161 | 0.6161 | 0.9768 | 0.9768 | 0.5711 | 0 | 0.567 | 0.9526 | 0.3788 | 0.93 | 0.6018 | 1.041 |
| 2 | rs10497616* | 183986209 | T | C | 0.6236 | 0.763 | 0.9725 | 0.9573 | 0.0016 | 84.49 | 0.001014 | 0.7076 | 0.2029 | 1.131 | 0.3826 | 1.086 |
| 12 | rs4931122* | 28992287 | A | G | 0.6344 | 0.7588 | 0.9785 | 0.9704 | 0.0101 | 78.25 | 0.03886 | 0.8425 | 0.3165 | 0.924 | 0.04154 | 1.166 |
| 8 | rs6988293* | 121303479 | A | G | 0.6354 | 0.7942 | 1.0216 | 1.0328 | 0.0006 | 86.67 | 0.0005764 | 1.326 | 0.502 | 0.949 | 0.08696 | 0.8804 |
| 2 | rs10514631* | 19767453 | C | T | 0.6473 | 0.9136 | 0.973 | 0.9848 | 0.0041 | 81.83 | 0.1484 | 1.172 | 0.3826 | 1.094 | 0.003746 | 0.751 |
| 7 | rs9639329* | 19366356 | C | T | 0.6537 | 0.6537 | 0.9789 | 0.9789 | 0.3952 | 0 | 0.193 | 0.8929 | 0.5582 | 1.05 | 0.8981 | 0.99 |
| 1 | rs4951701* | 209232629 | C | G | 0.6618 | 0.7351 | 0.9797 | 0.9753 | 0.0851 | 59.42 | 0.2613 | 0.9088 | 0.2043 | 0.901 | 0.1326 | 1.123 |
| 10 | rs2784773* | 81909780 | A | G | 0.6682 | 0.6682 | 0.9802 | 0.9802 | 0.5771 | 0 | 0.2869 | 0.9138 | 0.9128 | 0.991 | 0.7088 | 1.029 |
| 6 | rs2085672* | 153903143 | G | C | 0.6714 | 0.6776 | 1.0206 | 1.0208 | 0.3457 | 5.86 | 0.1522 | 1.133 | 0.6328 | 0.96 | 0.8776 | 0.9879 |
| 18 | rs9959583* | 20993778 | T | C | 0.6748 | 0.6748 | 0.9811 | 0.9811 | 0.7988 | 0 | 0.8449 | 1.016 | 0.9104 | 0.991 | 0.4479 | 0.9448 |
| 7 | rs193803* | 105394028 | A | G | 0.6776 | 0.7713 | 1.0226 | 1.0251 | 0.0804 | 60.33 | 0.9834 | 0.998 | 0.0493 | 1.201 | 0.2453 | 0.9026 |
| 3 | rs9862718* | 54399741 | A | G | 0.6787 | 0.7922 | 1.0221 | 1.0314 | 0.0073 | 79.68 | 0.004323 | 1.315 | 0.2787 | 0.905 | 0.3899 | 0.9288 |
| 6 | rs1220445* | 91712833 | A | G | 0.7004 | 0.7004 | 0.9828 | 0.9828 | 0.8777 | 0 | 0.8693 | 0.9866 | 0.8972 | 1.01 | 0.5452 | 0.956 |
| 16 | rs1075905* | 9015599 | C | T | 0.7045 | 0.8698 | 1.024 | 1.0246 | 0.0037 | 82.13 | 0.004284 | 1.37 | 0.0809 | 0.821 | 0.6627 | 0.9561 |
| 2 | rs2139404* | 100608026 | A | G | 0.7105 | 0.7993 | 1.0169 | 1.0174 | 0.1048 | 55.67 | 0.5314 | 0.9499 | 0.0512 | 1.166 | 0.5039 | 0.9515 |
| 9 | rs10491590* | 728940 | T | C | 0.7166 | 0.7776 | 1.032 | 1.0382 | 0.0981 | 56.93 | 0.04622 | 1.368 | 0.4303 | 0.884 | 0.6419 | 0.9373 |
| 20 | rs730520* | 7635252 | A | G | 0.7195 | 0.8541 | 0.9839 | 0.9878 | 0.1117 | 54.37 | 0.1845 | 1.114 | 0.8811 | 0.988 | 0.09762 | 0.884 |
| 2 | rs17644183* | 168343798 | C | T | 0.7222 | 0.7667 | 1.0165 | 1.0203 | 0.1154 | 53.69 | 0.2802 | 1.094 | 0.2676 | 1.093 | 0.1508 | 0.8966 |
| 10 | rs10764825* | 130559927 | G | A | 0.733 | 0.8283 | 0.9841 | 0.9824 | 0.0477 | 67.14 | 0.4409 | 1.067 | 0.0261 | 0.833 | 0.4131 | 1.065 |
| 7 | rs2707469* | 120764122 | G | A | 0.7358 | 0.8313 | 1.0208 | 1.0237 | 0.039 | 69.18 | 0.02169 | 1.28 | 0.3919 | 0.911 | 0.4281 | 0.9237 |
| 16 | rs2306743* | 34649292 | C | G | 0.7395 | 0.7958 | 0.9758 | 0.9623 | 0.0174 | 75.3 | 0.01363 | 0.7172 | 0.1443 | 1.193 | 0.8325 | 1.027 |
| 12 | rs6581677* | 65056646 | G | C | 0.7396 | 0.7939 | 1.0153 | 1.0216 | 0.0415 | 68.57 | 0.03341 | 1.193 | 0.9896 | 1.001 | 0.1629 | 0.9003 |
| 10 | rs7898110* | 53562357 | C | A | 0.747 | 0.8154 | 1.0161 | 1.026 | 0.0075 | 79.54 | 0.007352 | 1.274 | 0.7787 | 0.976 | 0.104 | 0.8749 |
| 7 | rs7808652* | 88881333 | C | A | 0.7508 | 0.7508 | 1.0174 | 1.0174 | 0.3992 | 0 | 0.3894 | 0.9184 | 0.7915 | 1.025 | 0.2861 | 1.101 |
| 11 | rs1528641* | 14043034 | A | T | 0.7678 | 0.7678 | 0.9867 | 0.9867 | 0.7436 | 0 | 0.6342 | 1.04 | 0.6999 | 0.97 | 0.5802 | 0.9594 |
| 5 | rs6893150* | 80545692 | A | G | 0.7729 | 0.9248 | 0.9843 | 0.9917 | 0.0729 | 61.81 | 0.1084 | 1.172 | 0.7839 | 0.973 | 0.1012 | 0.8659 |
| 10 | rs10998835* | 70922049 | A | G | 0.7894 | 0.7894 | 1.0123 | 1.0123 | 0.835 | 0 | 0.5179 | 1.055 | 0.8956 | 0.99 | 0.9831 | 0.9984 |
| 7 | rs851681* | 147107591 | A | C | 0.7909 | 0.9063 | 0.9873 | 0.9898 | 0.0384 | 69.32 | 0.08325 | 1.163 | 0.0594 | 0.853 | 0.8133 | 0.9816 |
| 3 | rs7645545 | 54392362 | T | C | 0.7954 | 0.8711 | 1.0126 | 1.024 | 0.0001 | 89.05 | 0.0002081 | 1.382 | 0.1454 | 0.883 | 0.1149 | 0.8831 |
| 1 | rs10918985* | 167022584 | A | C | 0.8028 | 0.885 | 0.9884 | 0.9904 | 0.1314 | 50.72 | 0.1586 | 1.127 | 0.1539 | 0.89 | 0.7362 | 0.9743 |
| 5 | rs10040249* | 110625639 | T | C | 0.8152 | 0.8152 | 1.0133 | 1.0133 | 0.7118 | 0 | 0.6173 | 1.054 | 0.6346 | 1.048 | 0.6121 | 0.9543 |
| 3 | rs2292518* | 112928236 | C | T | 0.8176 | 0.8176 | 1.0109 | 1.0109 | 0.5134 | 0 | 0.3475 | 1.084 | 0.8136 | 1.019 | 0.4976 | 0.949 |
| 13 | rs9554679* | 99787063 | A | G | 0.8203 | 0.8203 | 0.9897 | 0.9897 | 0.7798 | 0 | 0.4798 | 0.9431 | 0.8275 | 1.017 | 0.9488 | 1.005 |
| 1 | rs1728232* | 113366167 | A | G | 0.821 | 0.8926 | 1.0105 | 1.008 | 0.1968 | 38.49 | 0.2718 | 0.9125 | 0.9395 | 0.994 | 0.1492 | 1.116 |
| 10 | rs9299582* | 51974286 | G | A | 0.8221 | 0.8221 | 1.0104 | 1.0104 | 0.7193 | 0 | 0.4457 | 1.065 | 0.9673 | 1.003 | 0.7162 | 0.9725 |
| 7 | rs1528502* | 84932177 | G | A | 0.8221 | 0.8221 | 1.0112 | 1.0112 | 0.7989 | 0 | 0.8497 | 0.9829 | 0.5016 | 1.06 | 0.9228 | 0.9921 |
| 5 | rs7731633* | 85586892 | T | C | 0.8273 | 0.9236 | 1.01 | 1.0067 | 0.0988 | 56.8 | 0.8347 | 0.9829 | 0.206 | 0.904 | 0.08118 | 1.14 |
| 14 | rs10498420* | 49483793 | C | T | 0.8281 | 0.8281 | 1.0098 | 1.0098 | 0.5352 | 0 | 0.3121 | 1.086 | 0.9704 | 0.997 | 0.5986 | 0.9616 |
| 10 | rs11002539* | 79841011 | C | T | 0.8354 | 0.8354 | 0.9849 | 0.9849 | 0.922 | 0 | 0.8982 | 1.017 | 0.6655 | 0.946 | 0.9604 | 0.994 |
| 13 | rs912044* | 38962446 | G | A | 0.8403 | 0.8403 | 1.0092 | 1.0092 | 0.5488 | 0 | 0.3426 | 1.082 | 0.9596 | 1.004 | 0.5634 | 0.9574 |
| 11 | rs1461371* | 26351264 | T | A | 0.8538 | 0.8538 | 1.0117 | 1.0117 | 0.8646 | 0 | 0.8487 | 1.022 | 0.6608 | 1.049 | 0.7613 | 0.9683 |
| 12 | rs2178683* | 80465589 | T | C | 0.8694 | 0.899 | 1.0117 | 1.0147 | 0.0731 | 61.77 | 0.8993 | 1.017 | 0.0842 | 1.233 | 0.1307 | 0.8372 |
| 2 | rs6706469* | 240059548 | T | A | 0.8815 | 0.8815 | 0.9902 | 0.9902 | 0.3738 | 0 | 0.9469 | 1.008 | 0.2479 | 0.876 | 0.4198 | 1.093 |
| 4 | rs17661170* | 171411895 | G | A | 0.8843 | 0.8822 | 0.9921 | 0.9895 | 0.1796 | 41.76 | 0.6342 | 1.047 | 0.106 | 0.854 | 0.4285 | 1.072 |
| 21 | rs2833822* | 32726508 | C | T | 0.8949 | 0.9535 | 0.994 | 0.9965 | 0.1763 | 42.37 | 0.1394 | 1.13 | 0.5281 | 0.951 | 0.338 | 0.9306 |
| 3 | rs1473348 | 54384035 | C | T | 0.896 | 0.8996 | 1.0064 | 1.019 | 0.0001 | 89.33 | 0.0002411 | 1.381 | 0.2006 | 0.896 | 0.05563 | 0.8588 |
| 2 | rs542972* | 239032770 | T | C | 0.9001 | 0.9153 | 0.994 | 0.9909 | 0.0396 | 69.02 | 0.0601 | 0.8485 | 0.0875 | 1.153 | 0.896 | 0.9897 |
| 22 | rs2255957 | 40571318 | A | G | 0.9106 | 0.9251 | 1.0075 | 0.9809 | 0.0001 | 89.51 | 0.0002662 | 0.6376 | 0.0661 | 1.228 | 0.1098 | 1.193 |
| 2 | rs11888564* | 173274379 | A | G | 0.9117 | 0.952 | 1.0069 | 0.9915 | 0.0051 | 81.09 | 0.007805 | 0.7376 | 0.1043 | 1.181 | 0.3356 | 1.106 |
| 8 | rs2369548* | 80044352 | C | T | 0.9194 | 0.9153 | 1.0046 | 1.0062 | 0.1921 | 39.37 | 0.8582 | 1.015 | 0.1867 | 1.109 | 0.2134 | 0.9113 |
| 9 | rs10973109* | 36851407 | G | A | 0.9241 | 0.9241 | 0.9947 | 0.9947 | 0.8647 | 0 | 0.6729 | 0.9573 | 0.9104 | 0.989 | 0.7449 | 1.031 |
| 1 | rs1874406* | 64547498 | T | C | 0.9298 | 0.9842 | 0.9952 | 0.9983 | 0.0882 | 58.82 | 0.07682 | 1.187 | 0.272 | 0.901 | 0.462 | 0.9362 |
| 1 | rs1764844* | 217309442 | C | T | 0.9308 | 0.9308 | 0.9959 | 0.9959 | 0.7583 | 0 | 0.6554 | 1.039 | 0.9536 | 1.005 | 0.5487 | 0.9546 |
| 7 | rs193841* | 105374089 | T | C | 0.935 | 0.9287 | 1.0045 | 1.0082 | 0.0644 | 63.53 | 0.7964 | 1.026 | 0.1012 | 1.167 | 0.09699 | 0.8608 |
| 6 | rs416101* | 95090722 | C | A | 0.9375 | 0.9331 | 0.9959 | 0.9952 | 0.3057 | 15.61 | 0.9488 | 1.006 | 0.2244 | 0.893 | 0.3431 | 1.086 |
| 7 | rs1100508* | 124132594 | G | C | 0.9379 | 0.9108 | 0.9965 | 0.9912 | 0.047 | 67.29 | 0.2335 | 0.907 | 0.3114 | 0.923 | 0.0551 | 1.154 |
| 13 | rs9542253* | 69752150 | T | A | 0.9579 | 0.9599 | 1.0025 | 1.0056 | 0.0036 | 82.2 | 0.01172 | 1.234 | 0.027 | 0.835 | 0.884 | 0.9889 |
| 7 | rs6967565* | 8096253 | A | G | 0.9748 | 0.981 | 0.9984 | 0.9987 | 0.3255 | 10.89 | 0.2416 | 1.115 | 0.3687 | 0.922 | 0.7854 | 0.9771 |
| 2 | rs4340489* | 14583293 | A | G | 0.9752 | 0.9617 | 1.0014 | 1.0034 | 0.088 | 58.86 | 0.09783 | 1.145 | 0.1459 | 0.892 | 0.9343 | 0.9939 |
| 14 | rs11624725* | 89783362 | T | G | 0.9762 | 0.9315 | 0.9986 | 0.9937 | 0.0816 | 60.1 | 0.2663 | 0.9116 | 0.3984 | 0.934 | 0.07996 | 1.142 |
| 18 | rs7240074* | 25336210 | T | C | 0.9951 | 0.9951 | 1.0003 | 1.0003 | 0.6584 | 0 | 0.8971 | 1.011 | 0.4779 | 0.946 | 0.5766 | 1.043 |
| 14 | rs1189041* | 55931553 | C | A | 0.9957 | 0.9957 | 0.9995 | 0.9995 | 0.4635 | 0 | 0.4296 | 1.129 | 0.3413 | 0.86 | 0.905 | 1.017 |
| 5 | rs17651115* | 75475886 | C | G | 0.9984 | 0.9998 | 1.0001 | 1 | 0.3619 | 1.61 | 0.264 | 0.9115 | 0.8792 | 1.012 | 0.3833 | 1.068 |

CHR: Chromosome; BP: Base pair position; SNP: Single Nucleotide Polymorphism; A1: Reference allele; A2: Alternative allele; P: Fixed-effects p-value; P(R): Random-effects p-value; OR: Fixed-effects Odds Ratio; OR(R): Random-effects Odds Ratio; Q: p-value for heterogeneity of OR; I: effect size for heterogeneity of OR; The last six columns show the p and OR values obtained in each analyzed sample.

* SNPs selected by two-locus association analyses in the NXC-GWAS sample.

**According to UCSC genome browser (NCBI36/hg18) and dbSNP build 130.
