# Supplementary material for: A Colorectal Cancer Susceptibility New Variant at 4q26 in the Spanish Population Identified by Genome-Wide Association Analysis
Source: PLoS One. 2014 Jun 30;9(6):e101178. doi: 10.1371/journal.pone.0101178 (PMC4076321; doi:10.1371/journal.pone.0101178)
Supplement: Table S5 — Details of the results obtained in each sample from those SNPs that showed the best results in the global meta-analysis. (DOC) [file pone.0101178.s008.doc]

**Table S5.** Details of the results obtained in each sample from those SNPs that showed the best results in the global meta-analysis.

|  |  |  |  |  | **NXC-GWAS** | |  |  | **NXC-Val** | |  |  | **Epicolon** | |  |  |
| --- | --- | --- | --- | --- | --- | --- | --- | --- | --- | --- | --- | --- | --- | --- | --- | --- |
| **CHR** | **Gene^†^** | **SNP** | **BP^‡^** | **A1** | **MAF A** | **MAF U** | **p** | **OR** | **MAF A** | **MAF U** | **p** | **OR** | **MAF A** | **MAF U** | **p** | **OR** |
| 9q31.1 | *LINC00587* | rs1930551* | 104380162 | T | 0,08021 | 0,04307 | 0.000086 | 1.93 | 0,073 | 0,058 | 0.113 | 1.27 | 0,073 | 0,060 | 0.156 | 1.23 |
| 9q31.1 | *LINC00587* | rs10990158 | 104335927 | T | 0,08577 | 0,04688 | 0.000073 | 1.90 | 0,078 | 0,061 | 0.092 | 1.28 | 0,073 | 0,062 | 0.213 | 1.20 |
| 7q31.1 | *NRCAM* | rs2041001* | 107870335 | G | 0,07218 | 0,03933 | 0.000279 | 1.90 | 0,059 | 0,048 | 0.202 | 1.24 | 0,058 | 0,043 | 0.076 | 1.35 |
| 10q25.3 | *ABLIM1* | rs941853 | 116189165 | A | 0,1357 | 0,1935 | 0.000203 | 0.65 | 0,135 | 0,150 | 0.311 | 0.89 | 0,160 | 0,186 | 0.067 | 0.83 |
| 7p15.1 | *LOC402644* | rs4722778 | 28278588 | G | 0,2357 | 0,308 | 0.000092 | 0.69 | 0,260 | 0,273 | 0.445 | 0.93 | 0,230 | 0,258 | 0.074 | 0.85 |
| 12q21.33 | *LINC00615* | rs10506984* | 89217396 | G | 0,2542 | 0,2804 | 0.148900 | 0.87 | 0,249 | 0,279 | 0.086 | 0.85 | 0,255 | 0,304 | 0.003 | 0.78 |
| 9q31.1 | *LINC00587* | rs7039568 | 104361604 | T | 0,08021 | 0,04261 | 0.000068 | 1.95 | 0,073 | 0,060 | 0.180 | 1.22 | 0,073 | 0,062 | 0.273 | 1.17 |
| 9q31.1 | *LINC00587* | rs16921774* | 104336206 | T | 0,08559 | 0,04762 | 0.000114 | 1.87 | 0,073 | 0,060 | 0.182 | 1.22 | 0,073 | 0,063 | 0.285 | 1.17 |
| 9q31.1 | *LINC00587* | rs10990136 | 104298657 | T | 0,08125 | 0,04443 | 0.000118 | 1.90 | 0,069 | 0,057 | 0.193 | 1.22 | 0,070 | 0,062 | 0.379 | 1.14 |
| 5q21.1 | *ST8SIA4* | rs2120913* | 100096374 | A | 0,4063 | 0,4401 | 0.095990 | 0.87 | 0,414 | 0,447 | 0.099 | 0.87 | 0,399 | 0,444 | 0.015 | 0.83 |
| 9q31.1 | *LINC00587* | rs7024470 | 104361506 | G | 0,08209 | 0,04581 | 0.000220 | 1.86 | 0,073 | 0,060 | 0.167 | 1.23 | 0,073 | 0,064 | 0.356 | 1.14 |

CHR: Chromosome; SNP: Single Nucleotide Polymorphism; BP: Base pair position; A1: Reference allele (minor allele). The last twelve columns show the minor allele frequency in cases (MAF A), the minor allele frecuency in controls (MAF U) and, the p and the Odds Ratio (OR) values obtained in each analyzed sample.

* SNPs selected by two-locus association analyses in the NXC-GWAS sample.

^†^The nearest gene or the gene where the SNP is located.

^‡^According to UCSC genome browser (NCBI36/hg18) and dbSNP build 130.
